# Multi-Responsive Molecular Encapsulation and Release Based on Hydrogen-Bonded Azo-Macrocycle

**DOI:** 10.3390/molecules28114437

**Published:** 2023-05-30

**Authors:** Jinyang Wu, Xuan Sun, Xianghui Li, Xiaowei Li, Wen Feng, Lihua Yuan

**Affiliations:** College of Chemistry, Key Laboratory of Radiation Physics and Technology of the Ministry of Education, Institute of Nuclear Science and Technology, Sichuan University, Chengdu 610064, China; jinyangwu1993@163.com (J.W.); sunxuanchem@163.com (X.S.); xianghui.li@nrtmedtech.com (X.L.); lixw@scu.edu.cn (X.L.); wfeng9510@scu.edu.cn (W.F.)

**Keywords:** hydrogen-bonded macrocycle, multi-responsive, host–guest chemistry

## Abstract

Research on stimuli-responsive host–guest systems is at the cutting edge of supramolecular chemistry, owing to their numerous potential applications such as catalysis, molecular machines, and drug delivery. Herein, we present a multi-responsive host–guest system comprising azo-macrocycle 1 and 4,4′-bipyridinium salt **G1** for pH-, photo-, and cation- responsiveness. Previously, we reported a novel hydrogen-bonded azo-macrocycle 1. The size of this host can be controlled through light-induced E↔Z photo-isomerization of the constituent azo-benzenes. The host is found in this work to be capable of forming stable complexes with bipyridinium/pyridinium salts, and implementing guest capture and release with **G1** under light in a controlled manner. The binding and release of the guest in the complexes can also be easily controlled reversibly by using acid and base. Moreover, the cation competition-induced dissociation of the complex **1a_2_**⊃**G1** is achieved. These findings are expected to be useful in regulating encapsulation for sophisticated supramolecular systems.

## 1. Introduction

Uncovering the mystery of biomolecules such as proteins for controlling the capture and release process has aroused extensive research interests [1,2]. However, the complexity of natural macrocycles renders chemists to design simple artificial systems for exploring the mechanism behind them [3]. In this regard, macrocycle-based host–guest systems are considered as one of the options that can fulfill this task more or less in functioning as encapsulation and release modules [4]. Multiple-responsive host–guest systems are particularly interesting in terms of their versatility in applications under various operative conditions with the aid of acid/base [5,6,7], light [8,9], and competitive guests [10,11]. Macrocyclic hosts, one of the components for such modular systems, have emerged in increasing numbers in recent years as recognition motifs for guest binding and for guest capture and release [12] owing to their easy synthesis and programmable, adjustable cavity size and binding affinity. Typical examples include cyclodextrin [13,14], crown ether [15,16,17], calixarene [18,19,20], pillararene [5,21,22,23,24], and other macrocycles [25,26], which are widely utilized for constructing multiple-responsive host–guest systems [27,28,29,30,31,32] to implement binding and releasing functions. 

There are mainly three types of host–guest-responsive systems used in the field of supramolecular chemistry according to external stimuli: (a) one relying on guest molecules that are switched between capture and release according to chemical actions (e.g., pH switching and substrate competition), (b) the second type accomplishing the capture and release process using light irradiation—a clean, easily manipulated approach—for controlling complexation, and (c) the third type modulating the host–guest binding ability using electrochemical methods to change the charge and electron distributions by implementing electron transfer reactions [33]. Most of the known light-switchable azo-benzene systems involve the use of light-responsive guests rather than light-responsive hosts. It has been challenging to carry out the uptake and release of guests based on macrocyclic hosts alone. So far, there have been only a few examples using photo-responsive macrocycles, cryptands, and box-like molecules for the encapsulation and release of guest molecules [34,35,36,37,38,39,40]. A study by Sessler et al. revealed an expanded “Texas-sized” molecular box (AzoTxSB) that incorporates photo-responsive azo-benzene bridging subunits and anion recognition motifs, which allows various anionic substrates to be bound and released by using different forms of the box. Controlled release was achieved under multiple responsive conditions including light, pH, and anion competition [36]. Pillararene-based azo-macrocycle from Huang’s group also exhibited excellent capture and release of guests by light-controlled irradiation [38]. Our previous work demonstrated the feasibility of the quantitatively controlled release of guests under light stimulus with hydrogen-bonded azo-macrocycles [40]. Specifically, incorporating azo-benzene subunits into the core structure of cyclo[6]aramide creates a novel dynamic hydrogen-bonded azo-macrocycle host, which could form complexes with various bipyridinium salts. The size of this host can be controlled through light-induced E↔Z photo-isomerization of the constitutional azo-benzene moieties in the ring.

Two-dimensional hydrogen-bonded aramide macrocycles with shape persistency have exhibited plenty of unique features for guest binding [41,42,43] compared to more flexible crown ether and three-dimensional macrocycles, such as cyclodextrin and pillararenes. In addition, such cyclic compounds present superiority over their corresponding non-macrocyclic counterparts [44]. H-bonded aramide macrocycles have been applied to applications in recognition [45], extraction [46,47], separation [48], transmembrane channels [49,50], catalysis [51,52], and liquid-crystal materials [53]. In fact, the amide oxygen atoms pointing inward in the cavity and the variation in the number of internal H-bonds endow this class of H-bonded macrocycles with binding sites of affinities of different degrees for accommodating organic cations, ion-pair species, and even neutral molecules [54]. For instance, they are able to complex cationic guests such as diakylammonium [55,56], diquat [57], tropylium [45], paraquat [40,58], ferrocenium [59], and pyridinium [60]. Interestingly, host–guest complexation with cyclo[6]aramide and *α*-amino esters was found to induce chiroptical amplification, showing the pronounced enantiomeric and structural differentiation of *α*-amino esters [61]. The superiority of H-bonded azo-macrocycles was revealed in their ability for forging higher-order rotaxanes [62]. Notably, the box-directed aggregation of multiple H-bonded azo-macrocycles enabled the easy generation of a shish-kebab-like ring-in-rings dimeric superstructure [63].

**Scheme 1:**
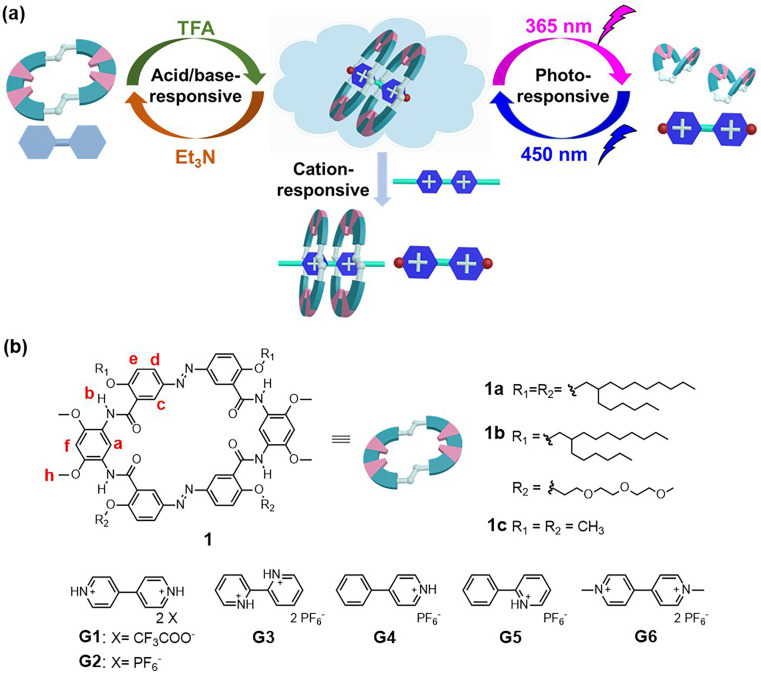
(**a**) Schematic representation of the multi-responsive encapsulation and/or release of 4,4′-bipyridinium salts by azo-macrocycle **1**, through photo-, acid/base-, and cation-competitive complexation-controlled process. (**b**) Chemical structures of azo-macrocycle **1** and guests **G1**–**G6**.

To be amenable to the changing environments, host–guest systems with multiple responsiveness are desirable in supramolecular chemistry. This provides an excess to the optimal use of several stimulus approaches for achieving desired functions [64,65,66]. However, multiple responsiveness remains unknown for these two-dimensional shape-persistent H-bonded aramide macrocycles. Bipyridinium/pyridinium salts and their derivatives (e.g., viologen) are popular guests among others in macrocycle chemistry. So far, the application of protonated bipyridine/pyridine salts as guests in host–guest chemistry is very limited [67]. The deprotonation of protonated bipyridine/pyridine salts often proceeds easily to provide bipyridines/pyridines, and vice versa, which could make the association and dissociation of the complexes based on the protonated bipyridine/pyridine salts easily chemically controlled by acid and base. In this study, we report a dual-responsive host–guest system based on H-bonded aramide azo-macrocycle **1** and protonated 4,4′-bipyridine **G1** and its analogues **G3**–**G5** (Scheme 1). The binding and release of the guests in the complexes can be easily controlled by light and acid/base for **G1**. In addition, the complexation is responsive to the presence of a competitive organic cation **G6**. Most multi-responsive host–guest systems reported hitherto rely on the use of hosts without light-responsive properties; instead, guests are photo-responsive. The dual-responsive host–guest system presented here is unique in the combined use of a host with photo-responsive property and a guest responsive to acid/base to enable switchable complexation.

## 2. Results and Discussion

### 2.1. Host–Guest Complexation

Hydrogen-bonded aramide macrocycle **1** used in the present work adopts a nearly planar conformation with six carbonyl oxygen atoms pointing inward as recognition sites. The cavity size measures 8.3 Å in diameter, which renders it suitable for enclosing such organic cations as pyridinium or bipyridinium (**G1**–**G5**) (4.1 Å in width) [68]. The host–guest complexation in solution was studied using ^1^H NMR, HRMS, and UV-vis spectroscopy. 

**Figure 1:**
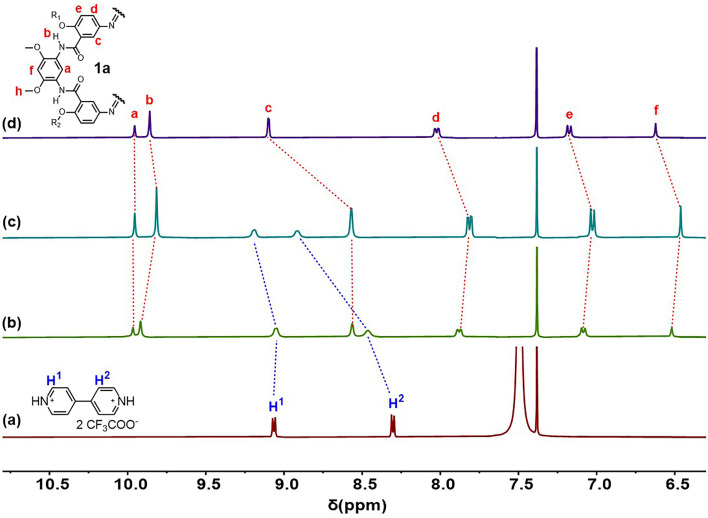
Partial ^1^H NMR spectra (400 MHz, CDCl_3_-CD_3_CN, 5:1, *v*/*v*, 298 K) of (**a**) **G1** (1.0 mM), (**b**) **1a** and **G1** (1.0 mM for each), (**c**) **1a** (2.0 mM) and **G1** (1.0 mM), and (**d**) **1a** (1.0 mM).

First, we set out to examine the complexation between host **1a** and guest **G1** in solution by ^1^H NMR spectroscopy. At room temperature, the ^1^H NMR spectra of a mixture of host **1a** and guest **G1** recorded in CDCl_3_-CD_3_CN show signals corresponding to the formation of a host–guest complex (Figure 1 and Figure S3). The signals of protons H^1^ and H^2^ in **G1** experience distinct downfield shifts, especially for the proton H^2^ with a shift as large as ca. 0.5 ppm at a 2:1 (H:G) molar ratio (Figure 1c) with respect to free guest (Figure 1a). Substantial upfield shifts for the aromatic protons *c* and *d* of the complex relative to the macrocycle (Figure 1c,d) were also observed. The signal evolution of these protons in the presence of 1 equiv. and 2 equiv. **1a** suggests the production of the complex **1a_2_**⊃**G1**. The Job plot experiment indicates the formation of a 2:1 complex (Figure S4), which is in accordance with the results from positive-ion electrospray ionization mass spectrometry (ESI-MS, Figure S5). UV-vis titration experiments in CHCl_3_-CH_3_CN (5:1, *v*/*v*) provided binding constants of *K*_1_ = 6.55 × 10^4^ M^−1^, *K*_2_ = 7.20 × 10^4^ M^−1^, and *K*_12_ = 4.72 × 10^9^ M^−2^ for **1a_2_**⊃**G1** (Figures S7 and S8). Notably, only a single set of signals from the bipyridinium ion was detected in the presence of 1.0 equiv. of **1a**, indicating a fast exchange on the NMR time scale for the complex formation (Figure 1b). When using **G2** with PF_6_^−^ as a different counterion to replace CF_3_COO^−^, a similar trend was observed (Figures S9–S13). The Job plot experiments provided a 2:1 stoichiometry for **1b_2_**⊃**G2** complexes in solution (Figures S11 and S12), which are all consistent with the results from the ESI-MS spectra (Figure S13). The binding constants of *K*_1_ = 5.63 × 10^4^ M^−1^, *K*_2_ = 8.24 × 10^3^ M^−1^, and *K*_12_ = 4.64 × 10^8^ M^−2^ (**G2**) were obtained in CHCl_3_-CH_3_CN (1:1, *v*/*v*) due to the low solubility in CHCl_3_-CH_3_CN (5:1, *v*/*v*) by fitting the experimental data from the UV-vis spectroscopy (Figures S14 and S15).

We then studied the complexation ability of **1a** with 2,2′-bipyridinium salt **G3**, a structural isomer of **G1**/**G2**. **G3** with **1a** also exhibited a fast exchange process on the NMR time scale (Figure S16). According to the results of the Job plot experiment (Figures S19 and S20), a host–guest complex with 2:1 stoichiometry was formed, which is in agreement with the HR-MS results (Figure S25). The association constants for **1a_2_**⊃**G3** were found to be *K*_1_ = 9.91 × 10^5^ M^−1^, *K*_2_ = 1.02 × 10^5^ M^−1^, and *K*_12_ = 1.01 × 10^11^ M^−2^ in CHCl_3_-CH_3_CN (5:1, *v*/*v*) (Figures S28 and S29). Since π–π stacking interactions play an important role along with multiple C-H···O hydrogen-bonding and/or N^+^···O ion–dipole interactions to form pseudorotaxanes [62], **G4**/**G5** with a single positive charge was selected to test if 2:1 stoichiometry could be retained under the same condition. **G4**, 4-phenylpyridinium salt, differs from **G5**, 2-phenylpyridinium salt, only in the position of nitrogen atoms. In the ^1^H NMR spectra, significant chemical shifts of **1a** and **G4**/**G5** could be observed, indicating the formation of host–guest complexes (Figures S17 and S18). The Job plot experiments only disclose the presence of 1:1 complexes between **1a** and **G4**/**G5** in solution (Figures S21–S24). HR-MS studies provided further evidence for the formation of the 1:1 complexes between **1a** and **G4**/**G5** (Figures S26 and S27), but no peaks relating to 2:1 species were observed. The binding constants for the 1:1 complex between **1a** and guest **G4**/**G5** were calculated to be *K_a_* = 5.50 × 10^3^ M^−1^ (**G4**) and *K_a_* = 4.02 × 10^4^ M^−1^ (**G5**) in CHCl_3_-CH_3_CN (5:1, *v*/*v*), respectively (Figures S30–S33).

The complexation of **G1** and **1a** was probed by two-dimensional nuclear overhauser effect spectroscopy (2D NOESY) in CDCl_3_-CD_3_CN. The sizable cross-peaks, (*c*, H^1^) and (*c*, H^2^), are apparently a consequence of the interactions through space between the macrocycle and guest **G1** (Figure S6). The additional NOE correlation signals could also be discerned between internal aromatic protons *a* of **1a** and protons H^1^/H^2^ in each pyridinium unit of **G1**. Since the two faces of the H-bonded aramide macrocycle are diastereotopic, NOEs were observed with protons on only one of its faces. The NOESY spectrum does not reveal any correlations associated with the interaction of side-chain protons of the host and internal aromatic protons. The through-space contact observed implies that the guest was trapped in the cavity of the macrocycle to form a **1a_2_**⊃**G1** pseudo[3]rotaxane. The results from these two cases remain coherent with the complexation process, in which the guest tends to reside in the cavity of macrocyclic host **1a**. 

### 2.2. Photo-Switchable Complexation

Following the study on the complexation between **1a** and protonated bipyridines/phenylpyridines, we carried out ^1^H NMR experiments to probe the controlled guest release and encapsulation by different stimuli. Among the reported stimuli, the use of light for manipulating guest binding and release is recognized as the most convenient means owing to its clean and benign process, fast response, and ease of spatio-temporal control [8]. H-bonded azo-macrocycle **1** used in this work features a molecular structure with two azo-benzene units incorporated into the macrocyclic backbone. Our previous work demonstrated that **1** is able to isomerize reversibly between E and Z configurations upon light irradiation [40]. The persistent shape due to the inherent nature of molecular rigidity and adaptable flexibility provides the macrocycle with an ability to quantitatively capture and release cationic guests by photo-irradiation. Protonated 4,4′-bipyridine **G1** was selected in this work for the following capture and release experiments.

Before exploring switchable complexation with light, we first examined photo-isomerization behavior under UV/BL light using ^1^H NMR spectroscopy (Figures S34 and S35). In the mixed solvent system of CDCl_3_ and CD_3_CN (5:1, *v*/*v*), azo-macrocycle **1a** exhibited pronounced light-induced E→Z isomerization. At the photo-stationary state, three isomers were identified as E,E-**1a** (10%), E,Z-**1a** (25%), and Z,Z-**1a** (65%), and the relative percentages were calculated by integrating the area of different stereoisomers (Figure S34). This result is consistent with prior observations in a more polar solvent system (CDCl_3_-CD_3_CN, 1:1, *v*/*v*) [40]. The light-induced isomerization process was finalized within 25 min as indicated by UV-vis (UV, 365 nm). A reverse process from Z,Z-**1a** to E,E-**1a** can be completed in less than 10 min when the sample solution is irradiated with blue light (BL, 450 nm) (Figure S35), indicating the high photo-sensitivity for such an azo-macrocycle. It is unlikely to convert all E,E-**1a** to Z,Z-**1a** under the experimental conditions, probably due to the rigidity of the macrocyclic backbone. According to the DFT calculations (B3LYP(PCM, chloroform)/6-31G (d,p)), the size of the macrocyclic cavity decreases substantially upon azo-benzene photo-isomerization (Figures S55–S57), which lays the foundation for the exclusion of guests through a reduction in cavity size, thereby ensuring the controlled binding and release of bipyridinium salts by using UV and BL irradiation.

**Figure 2:**
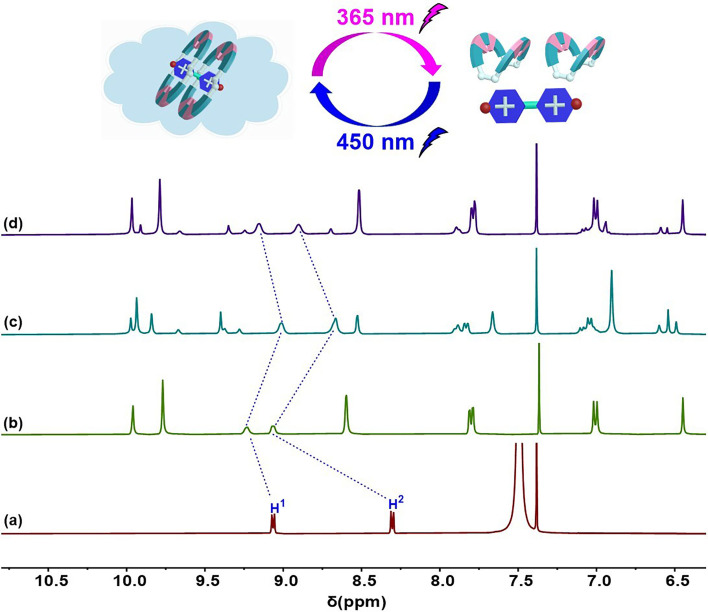
Partial ^1^H NMR spectra (400 MHz, CDCl_3_-CD_3_CN, 5:1, *v*/*v*, 298 K) of (**a**) **G1**, (**b**) **1a**:**G1** = 2:1 before UV irradiation, (**c**) **1a**:**G1** = 2:1 after UV irradiation, (**d**) under blue light irradiation of (**c**).

Indeed, when a mixture of **1a** and **G1** (**1a_2_**⊃**G1** complex) was exposed to photo-irradiation, the signals from protons H^1^ and H^2^ of E,Z-**1a** and Z,Z-**1a** appeared upfield (Figure 2b,c and Figure S36). The changes in the chemical shifts of signals from aromatic protons of **1a** were also observed. Specifically, the signal from the proton H^1^ experienced a pronounced upfield shift (Δδ = −0.23 ppm) relative to the proton resonance of the **1a_2_**⊃**G1** complex before UV irradiation, and the signal from the other proton H^2^ also shifted upfield to a great extent (Δδ = −0.39 ppm), strongly suggesting that the guest was released (Figure 2c). Reversely, when the sample solution was irradiated with BL, the guest **G1** was bound to the macrocycle again. This is indicated by the same signals reappearing at the same position, pointing to the regeneration of the E,E-**1a_2_**⊃**G1** complex. In fact, the signals from protons H^1^ and H^2^ had a pronounced downfield shift (Δδ = +0.14 ppm and +0.23 ppm, respectively) under blue light irradiation with respect to the same protons in solution after irradiation. It should be noted that not all the signals of protons H^1^ and H^2^ returned to the original resonances because the conversion from Z,Z-**1a** to E,E-**1a** was incomplete, which is normal in photo-isomerization for most azo-based compounds [69]. On the basis of these experimental observations, we conclude that neither E,Z-**1a** nor Z,Z-**1a** has an appreciable affinity for **G1** via complexation within the cavity. Upon the exposure of the **1a_2_**⊃**G1** complex to UV light irradiation, the E→Z transformation of the macrocycle occurred, leading to the reduced size of the cavity and, thus, the release of **G1**. Exposure of the irradiated mixture to light at 450 nm reset the system back to the guest-trapped E,E-**1a_2_**⊃**G1**-enriched state. 

**Figure 3:**
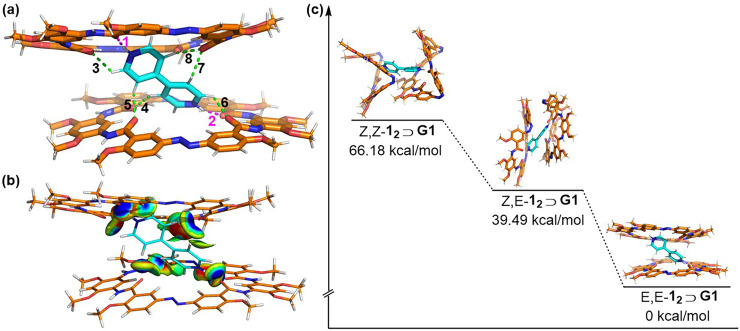
(**a**) Optimized structure at the DFT/B3LYP(PCM, chloroform)/6-31G (d,p) level of complex E,E-**1c_2_**⸧**G1**. (H—white, O—red, N—blue, C—orange and cyan). All side chains are replaced by methyl groups for simplicity, and the counterion CF_3_COO^−^ is omitted for clarity. The dashed magenta lines indicate ^+^N-H···O hydrogen bonds 1 and 2, where 1 = 1.7 Å (159.1°) and 2 = 1.7 Å (165.6°). The dashed green lines indicate C-H···O hydrogen bonds 3-8, where 3 = 2.3 Å (122.6°), 4 = 2.3 Å (141.7°), 5 = 2.1 Å (147.0°), 6 = 2.2 Å (131.8°), 7 = 2.4 Å (139.7°), and 8 = 2.2 Å (160.5°). (**b**) Visual study of weak interaction (IGM analyses), revealing the multiple hydrogen bond interactions between **1c** and **G1**. (**c**) Structure, relative energies of E,E-**1c_2_**⸧**G1**, E,Z-**1c_2_**⸧**G1,** and Z,Z-**1c_2_**⸧**G1**. The energy is measured in kcal/mol and compared to the lower energy conformation of E,E-**1c_2_**⸧**G1**, which is set to 0 kcal/mol.

With a view to gaining further understanding of the detected binding properties, DFT-technique-based computational simulations were performed for the host–guest complex system comprising azo-benzene macrocycle **1c** and protonated 4,4′-bipyridine at the B3LYP(PCM, chloroform)/6-31(d,p) level (Figure 3 and Figures S55–S57). The results of the binding energies disclosed that each guest molecule resides in the macrocyclic cavity through multiple hydrogen-bonding interactions (Figure 3a). For example, in the case of **G1**, there exist six C-H∙∙∙O hydrogen bonds and two ^+^N-H∙∙∙O hydrogen bonds in the complex (Figure 3a). An independent gradient model (IGM) analysis (Figure 3b, Figures S58 and S59) revealed the multiple H-bond interactions between **1c** and **G1**. DFT calculations led to the conclusion that the lowest energy for the molecular configuration of Z,Z-**1c_2_**⊃**G1** is 26.69 kcal/mol higher in energy than that of E,Z-**1c_2_**⊃**G1**, which lies 39.49 kcal/mol above E,E-**1c_2_**⊃**G1** (Figure 3c). These findings are consistent with the notion that E,E-**1a_2_**⊃**G1** is more stable than E,Z-**1a_2_**⊃**G1** or Z,Z-**1a_2_**⊃**G1**.

The results above demonstrate the reversibility of the present complexation system working under UV and BL light irradiation to a greater extent. Interestingly, the release and encapsulation of guests from the complexes **1a_2_**⊃**G2**, **1a_2_**⊃**G3**, **1a**⊃**G4**, and **1a**⊃**G5** were not observed under photo-responsive conditions (Figures S37–S40). We hypothesized that the proton transfers from **G2**–**G5** to the azo-benzene groups of **1a** may occur, thereby blocking photo-isomerization. Protonation experiments were carried out to provide an important piece of evidence for the claim that protonated azo groups are unable to isomerize. Indeed, macrocycle **1a** failed to isomerize in the presence of TFA (Figure S45). We noticed that guests **G2**–**G5** were in a state of dynamic equilibrium. When mixing bipyridinium (or pyridinium) salt (**G2**–**G5**) with bipyridine (or pyridine), only a signal set of signals (Figures S41–S44) appear, indicative of the occurrence of proton transfer. Therefore, the observation for the failure in guest capture and uptake with **G2**–**G5** under light irradiation is attributable to the rigidification of the molecular backbone through the protonation of azo groups in the macrocycle.

### 2.3. Acid/Base-Switchable Complexation

Switchable complexation in the presence of acid/base is usually enabled by the preferential binding of one guest over another. Bipyridine and pyridine are known to be easily protonated, providing bipyridinium and pyridinium salts in solution, and a reverse process is induced by a base such as Et_3_N. The H-bonded azo-macrocycle **1a** is capable of binding positively charged **G1**–**G5** and has no or very low affinity for neutral bipyridine/pyridine. This constitutes the basis for the chemically controlled association and dissociation of the complexes by acid and base.

**Figure 4:**
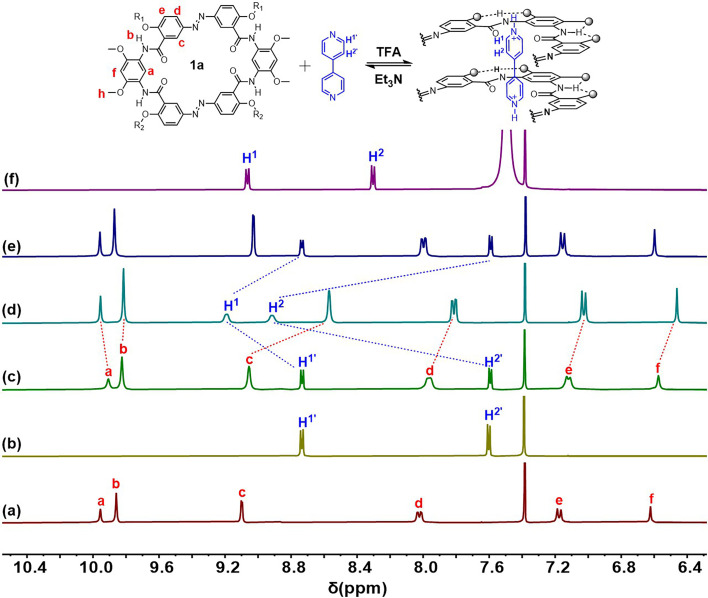
Partial ^1^H NMR spectra (400 MHz, CDCl_3_-CD_3_CN, 5:1, *v*/*v*, 298 K) of (**a**) free **1a**, (**b**) free 4,4′-bipyridine, (**c**) 4,4′-bipyridine and **1a** (2.0 equiv.), (**d**) the solution of (**c**) in the presence of 4.0 equiv. of TFA, (**e**) the solution of (**d**) after adding 6.0 equiv. of Et_3_N, and (**f**) free **G1**. [**1a**]_0_ = 2.0 mM.

To this end, we first tested the interaction between **1a** and 4,4′-bipyridine. The ^1^H NMR spectrum shows slight changes (~0.02 ppm) for the 2:1 mixture of host and guest in CDCl_3_-CD_3_CN (5:1, *v*/*v*) (Figure 4a–c and Figure S46). We attempted to determine the stoichiometry and the binding constant between **1a** and 4,4′-bipyridine. The Job plot experiment indicates the formation of a 2:1 complex (Figures S47 and S48); however, the binding constant fitting based on the data from the ^1^H NMR titration experiments failed (Figure S49), indicating that the binding affinity between **1a** and 4,4′-bipyridine is too low to be determined [70]. Therefore, the effect of neutral bipyridine on the binding event is negligible. When 4.0 eq. of trifluoroacetic acid (TFA) was added to the solution, the aromatic ArH resonances (*c*, *d*, *e*, and *f*) for **1a** experienced an upfield shift by Δδ = −0.49, −0.16, −0.18, and −0.11 ppm, respectively (Figure 4c,d), while protons H^1^ and H^2^ of guest **G1** exhibited a downshift of Δδ = +0.12 and +0.91 ppm, respectively (Figure 4c,f). Comparing the spectrum (Figure 4c) of the mixture with that of the complex E,E**-1a_2_**⊃**G1** (Figure 1c) indicates that a stable complex **1a_2_**⊃**G1** was formed. As excess triethylamine (Et_3_N, 6 eq.) is added to the solution above, deprotonation occurs on **G1**, and as a consequence, the complex **1a_2_**⊃**G1** dissociates (Figure 4e). This is clearly indicated by the reappearance of the resonance from 4′4-bipyridine at the original position. A similar change in signals is observed with **1a**. These results demonstrate that the capture and release of **1a_2_**⊃**G1** could be induced by adding in sequence TFA and Et_3_N. The complexes **1a_2_**⊃**G2**, **1a_2_**⊃**G3**, **1a**⊃**G4**, and **1a**⊃**G5** can be regulated in a similar fashion (Figures S50–S53).

### 2.4. Competitive Ion Complexation

Tuning host–guest affinity may provide a useful alternative for the control of complicated switchable supramolecular systems. H-bonded azo-macrocycle **1a** has a strong binding ability toward N,N-dialkyl-4,4′-bipyridinium salts. This promoted us to further examine the cation competition with a host–guest system comprising **1a** and **G2** (PF_6_^−^ as a counter ion) in the presence of **G6** as a competitive ion to release protonated 4,4′-bipyridine **G2** (Figure S54).

**Figure 5:**
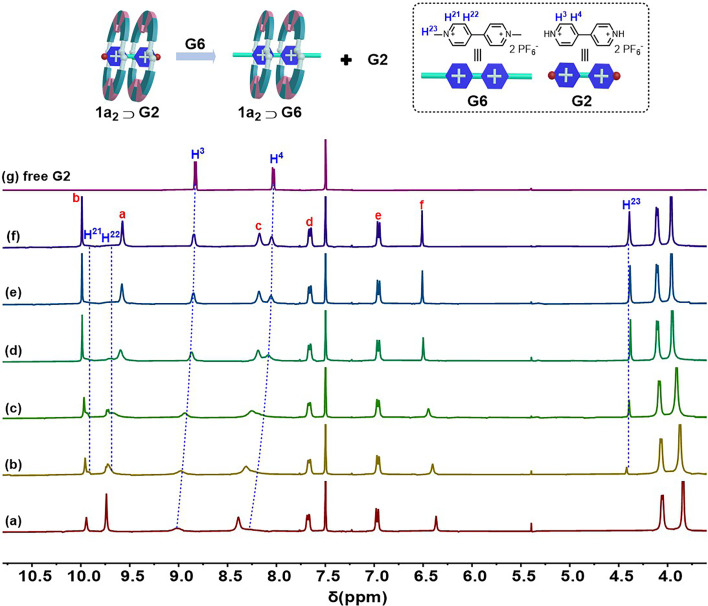
Partial ^1^H NMR spectra (400 MHz, CDCl_3_-CD_3_CN, 1:1, *v*/*v* 298 K) of (**a**) 2.0 mM **1a** and 1.0 mM **G2**, and containing the following concentrations of **G6** (**b**) 0.2 mM, (**c**) 0.4 mM, (**d**) 0.8 mM, (**e**) 1.0 mM, (**f**) 1.2 mM, (**g**) free **G2**.

Firstly, when macrocycle **1a** (2.0 mM) was mixed with guest **G2** (1.0 mM) in CDCl_3_-CD_3_CN (1:1, *v*/*v*), a stable **1a_2_**⊃**G2** complex was formed (Figure 5a). The ^1^H NMR spectra show signals of protons H^3^ and H^4^ of **G2**, which shift upfield by Δδ = −0.16 and −0.2 ppm, respectively. Accompanied by the change in chemical shifts is the appearance of signals corresponding to the formation of the complex **1a_2_**⊃**G6** (Figure 5 and Figure S54). The protons H^21^ and H^22^ show board signals because of the presence of rotational isomerization in this system [71]. Beyond 1.0 mM of **G6**, signals from protons H^3^ and H^4^ of guest **G2** return by shifting upfield to the position where free **G2** resonates (Figure 5e,g). These results indicate that the complex **1a_2_**⊃**G6** is formed upon the dissociation of the complex **1a_2_**⊃**G2**, thereby accomplishing the release of guest **G2** through a cation competition process. Unfortunately, this process is irreversible due to the much stronger binding affinity of paraquat **G6** over **G2** [40].

## 3. Materials and Methods

### 3.1. Materials and Reagents

4,4′-Bipyridine, 2,2′-bipyridine, 4-phenylpyridine, 2-phenylpyridine, CDCl_3_, and HPLC CD_3_CN were purchased from Energy Chemical. CD_3_CN was purchased from Cambridge Isotope Laboratories (CIL). HPLC CHCl_3_ and CH_3_CN were purchased from Chron Chemicals (Chengdu, China). All reagents purchased from commercial suppliers were used without further purification.

### 3.2. Experimental Methods

NMR Analytical NMR spectra were recorded on Bruker AVANCE AV II-400/600 MHz at a temperature of 298 K (^1^H: 400 MHz; 2D: 600 MHz). Chemical shifts were reported in δ values in ppm using tetramethylsilane (TMS) or residual solvent as internal standard, and coupling constants (J) are denoted in Hz. Multiplicities are denoted as follows: s—singlet, d—doublet, t—triplet, dd—double doublet, and m—multiplet. High-resolution mass (HRMS) data were collected by WATERS Q-TOF Premier. UV-vis spectra were measured by SHIMADZU UV-2450 (Kyoto, Japan).

### 3.3. Synthesis of Azo-Macrocycles **1** (**1a** and **1b**) and Guests **G1**–**G6**

Azo-macrocycles **1** (**1a** and **1b**) and guests **G1**–**G6** were synthesized according to previous references [40,67,72,73] (Schemes S1 and S2, Figures S1 and S2).

### 3.4. DFT Calculations

DFT calculations for the geometrical optimizations were performed in Gaussian 09 program package [74]. All substituents (R_1_ and R_2_) on the periphery of azo-macrocycle **1** were replaced by methyl groups to provide compound **1c** for simplicity. The counterion CF_3_COO^−^ of guest **G1** was also omitted for clarity.

### 3.5. Visualization of Noncovalent Interactions

Independent gradient model (IGM) analysis is an approach [75] based on promolecular density (an electron density model prior to molecule formation) to identify and isolate intermolecular interactions. DFT optimize structures are used as input files. Strong polar attractions, van der Waals contacts, and repulsive forces are visualized as an isosurface with blue, green, and red colors, respectively. The binding surface was calculated by Multiwfn 3.8 program [76] and visualized using PyMOL [77].

## 4. Conclusions

In summary, we demonstrated a photo-responsive and acid/base-controllable host–guest system based on hydrogen-bonded azo-macrocycle **1** and protonated 4,4′-bipyridine. The complexation and dissociation of the complexes were explored under UV/BL irradiation and acid/base conditions. Switchable complexation was corroborated and rationalized by using the results from ^1^H-NMR, HR-MS, binding constant, and titration experiments. Moreover, control can also be achieved using cation competition. The multi-responsive host–guest systems in this work may serve as a potential platform to construct multi-component and complicated molecular machines. Correlational research is underway in our laboratory. 

## Data Availability

Not applicable.

## Supplementary Materials

The following supporting information can be downloaded at: https://www.mdpi.com/article/10.3390/molecules28114437/s1, Scheme S1: Synthetic routes of **1a** and **1b**, Scheme S2: Synthetic routes of guests **G1**–**G6**, Figure S1: ^1^H NMR spectrum of **1a**, Figure S2: ^1^H NMR spectrum of **1b**, Figure S3: Stacked ^1^H NMR spectra of **1a_2_**⊃**G1**, Figure S4: Job plot for the stoichiometry of **1a** and **G1**, Figure S5: HR-MS spectrum of **1a_2_**⊃**G1**, Figure S6: 2D NOESY spectrum of **1a_2_**⊃**G1**, Figures S7 and S8: Stacked UV-vis spectra of binding constant experiment and corresponding fitting curves of **1a** and **G1**, Figure S9: Stacked ^1^H NMR spectra of **1a_2_**⊃**G3, Figure S10**: 2D NOESY spectrum of **1a_2_**⊃**G3**, Figures S11 and S12: Job plot for the stoichiometry of **1a** and **G3**, Figure S13: HR-MS spectrum of **1a_2_**⊃**G3**, Figures S14 and S15: Stacked UV-vis spectra of binding constant experiment and corresponding fitting curves of **1a** and **G3**, Figures S16–S18: Stacked ^1^H NMR spectra of **1a_2_**⊃**G3**, **1a_2_**⊃**G4**, and **1a_2_**⊃**G5**, Figures S19–S24: Job plot for the stoichiometry of **1a** and **G3**–**G5**, Figures S25–S27: HR-MS spectra of **1a_2_**⊃**G3**, **1a_2_**⊃**G4**, and **1a_2_**⊃**G5**, Figures S28–S33: Stacked UV-vis spectra of binding constant experiment and corresponding fitting curves of **1a** and **G3**–**G5**, Figures S34 and S35: ^1^H NMR spectra of **1a** photo-irradiation, Figures S36–S40: ^1^H NMR spectra of photo-switchable complexation, Figures S41–S44: ^1^H NMR spectra of proton transfer between bipyridine/phenylpyridine and bipyridinium/phenylpyridinium, Figure S45: ^1^H NMR spectra of protonated **1a** photo-irradiation, Figures S46–S53: ^1^H NMR spectra of acid/base-switchable complexation, Figure S54: ^1^H NMR spectra of competitive ion complexation, Figures S55–S57: DFT simulation of E,E-**1c_2_**⊃**G1**, E,Z-**1c_2_**⊃**G1**, and Z,Z-**1c_2_**⊃**G1**, Figures S58 and S59: Visualization of noncovalent interactions in complex **1c_2_**⊃**G1**.

## Sample Availability

Samples of compounds **1a**, **1b** and **G1**–**G6** are available from the authors.
