# Supplementary material for: Multi-Responsive Molecular Encapsulation and Release Based on Hydrogen-Bonded Azo-Macrocycle
Source: Molecules. 2023 May 30;28(11):4437. doi: 10.3390/molecules28114437 (PMC10254748; doi:10.3390/molecules28114437)
Supplement: Supplementary file 1 [file molecules-28-04437-s001.zip › molecules-2372016-supplementary.pdf]

Supporting Information

Multi-Responsive Molecular Encapsulation and Release Based on Hydrogen-Bonded Azo-macrocycle

Jinyang Wu, Xuan Sun, Xianghui Li, Xiaowei Li, Wen Feng and Lihua Yuan *

College of Chemistry, Key Laboratory of Radiation Physics and Technology of Ministry of Education, Institute of Nuclear Science and Technology, Sichuan University, Chengdu 610064, China; Jinyangwu1993@163.com (J.W.); Sunxuanchem@163.com (X.S.); xianghui.li@nrtmedtech.com (X.L.); lixw@scu.edu.cn (X.L.); wfeng9510@scu.edu.cn (W.F.)

** Correspondence: lhyuan@scu.edu.cn (L.Y.)*

Table of Contents

Synthesis and characterization 3

Host-guest chemistry of 1a and G1 6

^{1}H NMR spectra for **1a** and **G1** interactions 6

Job plot for determination of stoichiometry 7

HRMS spectrum of **G1⊂1a**$_2$ 7

2D NOESY spectrum for **G1⊂1a**$_2$ 8

UV-vis titration experiments 8

Host-guest chemistry of 1a and G2 10

^{1}H NMR spectra for **1a** and **G2** interactions 10

2D NOESY spectrum for **G2⊂1a**$_2$ 11

Job plot for determination of stoichiometry 12

HRMS spectrum of **G2⊂1a**$_2$ 13

UV-vis titration experiments 13

^{1}H NMR spectra of host-guest complexes 14

Job plot for determination of stoichiometry 16

HRMS spectra of complexes 19

UV-vis titration experiments 20

Photo-responsiveness of host 1a 23

Photo-responsive binding and release of the guests in G1⊂1a$_2$ 24

Photo-responsive of host-gust complex G2⊂1a$_2$**, G3⊂1a**$_2$**, G4⊂1a, and G5⊂1a** 24

Acid/base responsive of host-guest complexes 29

Cation-responsive release of the guest in G2⊂1a$_2$ 34

DFT calculation studies 34

References 49

Synthesis and characterization

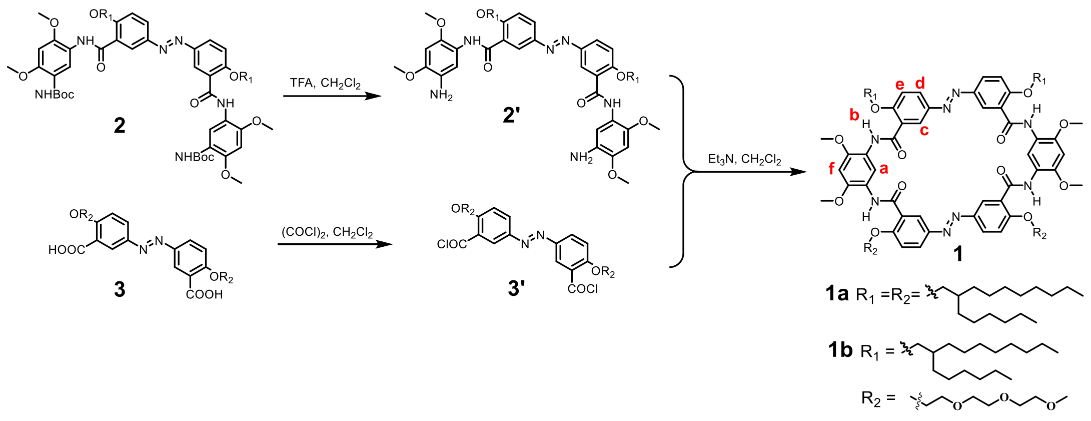

Scheme S1 Synthetic route of **1a** and **1b.**

1a and **1b** were prepared according to literature procedures. [1] Because of the poor solubility of macrocycle **1a** in $CDCl_3$-CD_3CN (1:1, v/v), **1b** bearing two triglycol side chains was prepared. In Job plot experiments, **1b** was used to study the H-G interactions.

1a: yellow solid powder (yield: 64.3 %). ^{1}H NMR (400 MHz, $CDCl_3$, 298 K): δ 9.98 (s, 2H), 9.75 (s, 4H), 9.24 (d, J = 2.6 Hz, 4H), 7.99 (dd, J = 8.7, 2.7 Hz, 4H), 7.09 (d, J = 8.8 Hz, 4H), 6.53 (s, 2H), 4.15 (d, J = 6.3 Hz, 8H), 3.91 (s, 12H), 2.12 (p, J = 6.2 Hz, 4H), 1.63 – 1.15 (m, 108H), 0.85 (tt, J = 6.8, 3.3 Hz, 24H).

1b: yellow solid powder (yield: 67.0 %). ^{1}H NMR (400 MHz, $CDCl_3$, 298 K) δ 9.97 (s, 2H), 9.79 (d, J = 8.3 Hz, 4H), 9.23 (dd, J = 6.2, 2.6 Hz, 4H), 7.99 (dt, J = 8.7, 2.5 Hz, 4H), 7.10 (dd, J = 8.9, 5.8 Hz, 4H), 6.53 (s, 2H), 4.47 (t, J = 5.2 Hz, 4H), 4.15 (d, J = 6.2 Hz, 4H), 4.07 (t, J = 5.3 Hz, 4H), 3.93 (d, J = 16.3 Hz, 12H), 3.75 (dd, J = 5.8, 3.7 Hz, 4H), 3.67 – 3.55 (m, 8H), 3.51 – 3.44 (m, 4H), 3.33 (s, 4H), 1.58 (s, 26H), 1.25 (d, J = 9.4 Hz, 3H), 0.86 (td, J = 7.0, 6.6, 2.1 Hz, 10H), 0.07 (s, 15H).

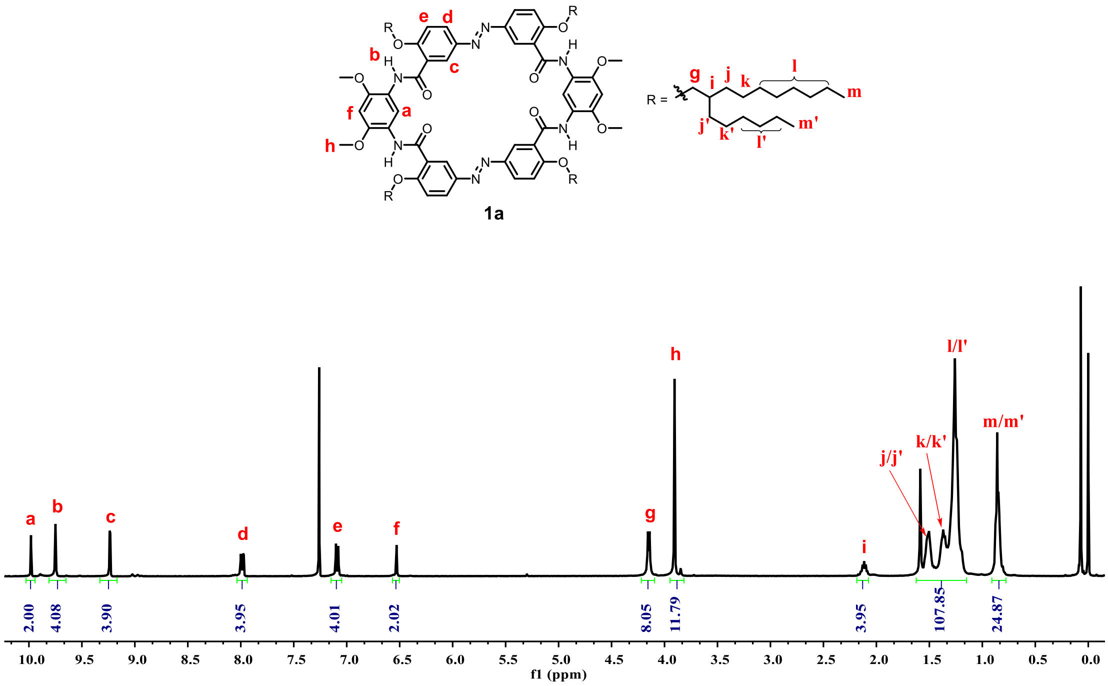

Figure S1 ^{1}H NMR spectrum (400 MHz, $CDCl_3$, 298 K) of **1a**.

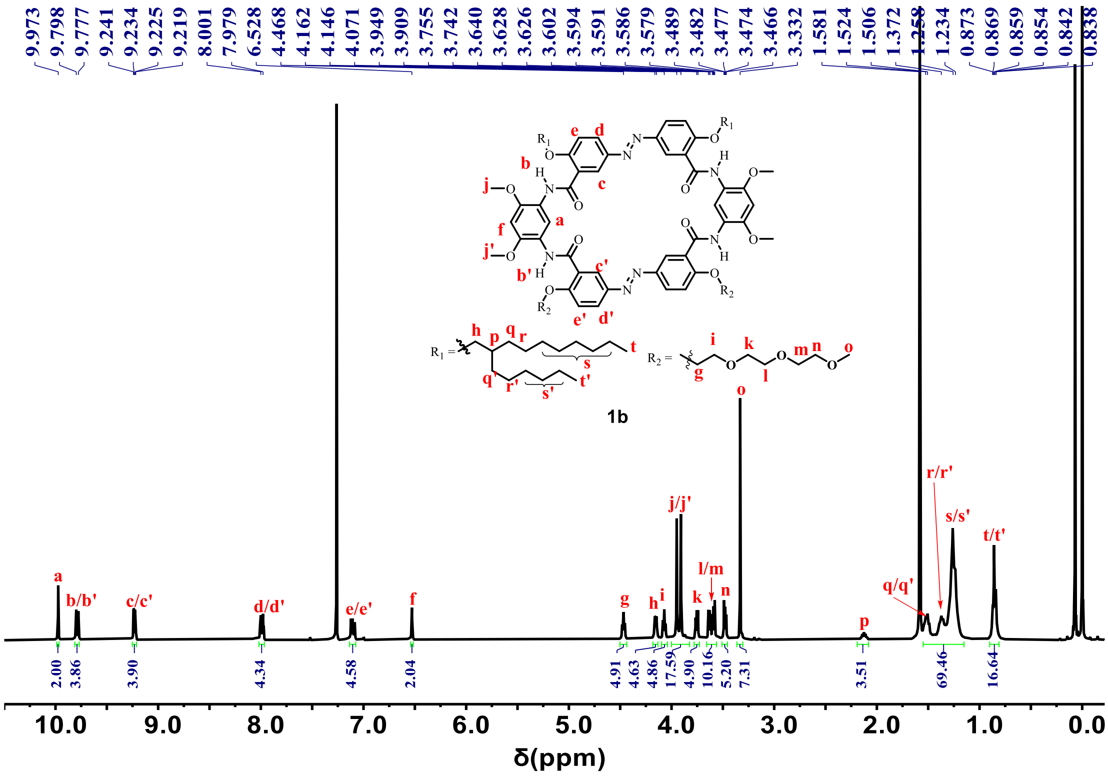

Figure S2 ^{1}H NMR spectrum (400 MHz, $CDCl_3$, 298 K) of **1b**.

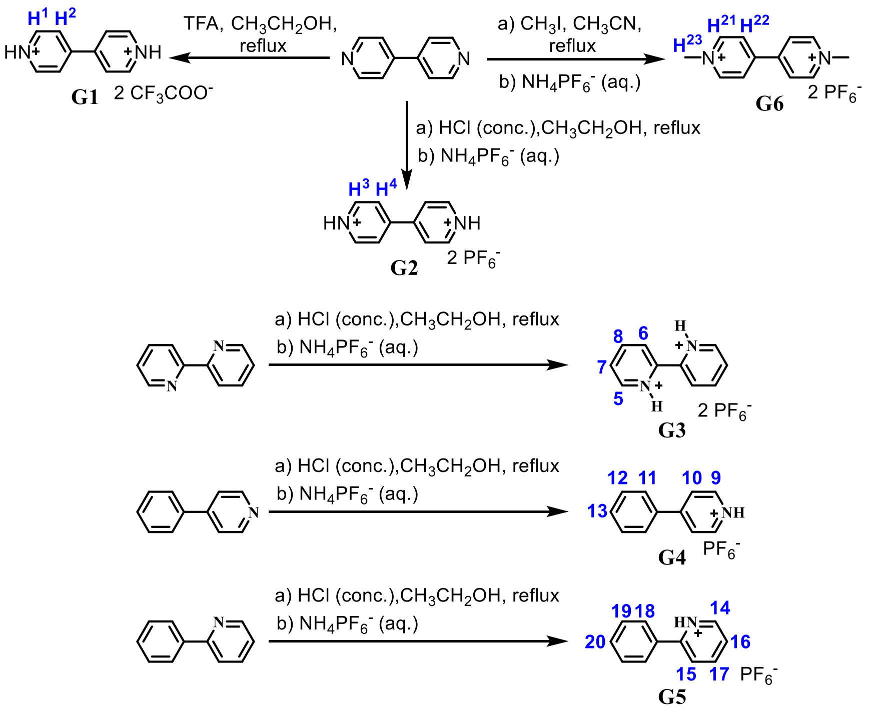

Scheme S2 Synthetic routes of guests **G1-G6.**

Guest **G1** [2]

To a solution of 4,4'-bipyridine (235 mg, 1.50 mmol) in CH_3CH_2OH (5 mL), TFA (25 mL) was added. The mixture was stirred for overnight at 80 ℃. The reaction mixture was added ice CH_3CH_2OH (100 mL) under ice bath after being cooled to room temperature. After filtration, the precipitate was collected and washed with ice CH_3CH_2OH (10 mL×3). After being dried in vacuum for 24 h, guest **G1** (476 mg, 83.0%) was obtained as a white powder. ^1H NMR (400 MHz, CD_3CN, 298 K) δ 8.97 – 8.88 (m, 4H, H^1), 8.16 – 8.09 (m, 4H, H^2).

Guest **G2** [3]

To a solution of 4,4'-bipyridine (235 mg, 1.50 mmol) in CH_3CH_2OH (5 mL), HCl (conc.) (25 mL) was added. The mixture was stirred for overnight at 80 ℃. The reaction mixture was added ice CH_3CH_2OH (100 mL) under ice bath after being cooled to room temperature. The mixture was filtered and washed with ice CH_3CH_2OH (10 mL×3). The solid was dissolved in water. The saturated aqueous solution of NH_4PF_6 was slowly added to the solution above and the mixture was stirred for 0.5 hr. After filtration, the precipitate was collected and washed with H_2O. After being dried in vacuum for 24 h, guest **G2** (430 mg, 48.3%) was obtained as a white powder. ^1H NMR (400 MHz, CD_3COCD_3, 298 K) 9.13 – 9.06 (m, 4H, H^3), 8.38 – 8.32 (m, 4H, H^4).

G3-G5 were prepared according to similar procedures of **G2**.

G3: white solid powder (yield: 51.6 %). [3] ^1H NMR (400 MHz, CD_3CN, 298 K) δ 8.85 (ddd, J = 5.3, 1.6, 0.9 Hz, 2H, *5*), 8.51 (dt, J = 8.1, 1.1 Hz, 2H, *6*), 8.44 (td, J = 7.8, 1.6 Hz, 2H, *7*), 7.91 (ddd, J = 7.6, 5.3, 1.3 Hz, 2H, *8*).

G4: white solid powder (yield: 54.3 %). ^{1}H NMR (400 MHz, $CDCl_3$-CD_3CN, 5:1, v/v, 298 K) δ 8.71 (d, J = 5.7 Hz, 2H, *9*), 7.94 – 7.87 (m, 2H, *10*), 7.79 – 7.73 (m, 2H, *11*), 7.60 – 7.54 (m, 3H, *12/13*).

G5: white solid powder (yield: 56.8 %). ^{1}H NMR (400 MHz, $CDCl_3$-CD_3CN, 5:1, v/v, 298 K) δ 8.78 (ddd, J = 6.0, 1.7, 0.8 Hz, 1H, *14*), 8.62 (td, J = 8.0, 1.6 Hz, 1H, *15*), 8.25 (dt, J = 8.3, 1.0 Hz, 1H, *16*), 8.01 (ddd, J = 7.5, 6.0, 1.3 Hz, 1H, *17*), 7.90 – 7.85 (m, 2H, *18*), 7.75 – 7.65 (m, 3H, *19/20*).

Guest **G6** [4]

G6 was prepared according to reference procedures. To a solution of 4,4'-bipyridine (400 mg, 2.60 mmol) in CH_3CN (15 mL), CH_3I (3 mL) was added. The mixture was stirred overnight at 80 ℃. The reaction mixture was filtered after being cooled to room temperature. The solid was then washed with CH_3CN and dissolved in water. The saturated aqueous solution of NH_4PF_6 was slowly added to the solution above and the mixture was stirred for 0.5 hr. After filtration, the precipitate was collected and washed with H_2O. After being dried in vacuum for 24 h, **G6** (1.02 g, 91.0 %) was obtained as a white powder. ^{1}H NMR (400 MHz, CD_3CN, 298 K) δ 8.86 – 8.79 (m, 4H, *H^{21}*), 8.35 (d, J = 6.3 Hz, 4H, *H^{22}*), 4.38 (s, 6H, *H^{23}*).

Host-guest chemistry of 1a and G1

^{1}H NMR spectra for 1a and G1 interactions

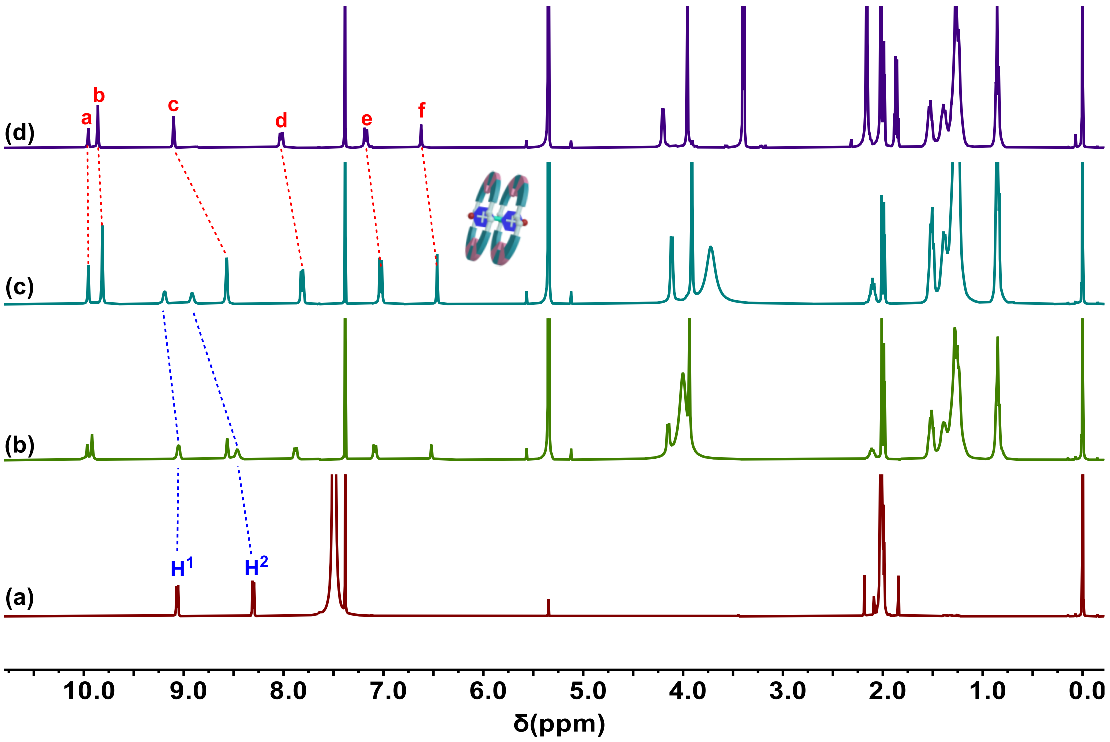

Figure S3 ^{1}H NMR spectra (400 MHz, $CDCl_3$-CD_3CN, 5:1, v/v, 298 K) of (a) **G1** (1.0 mM), (b) **1a** and **G1** (1.0 mM for each), (c) **1a** (2.0 mM) and **G1** (1.0 mM) and (d) **1a** (1.0 mM).

Job plot for determination of stoichiometry

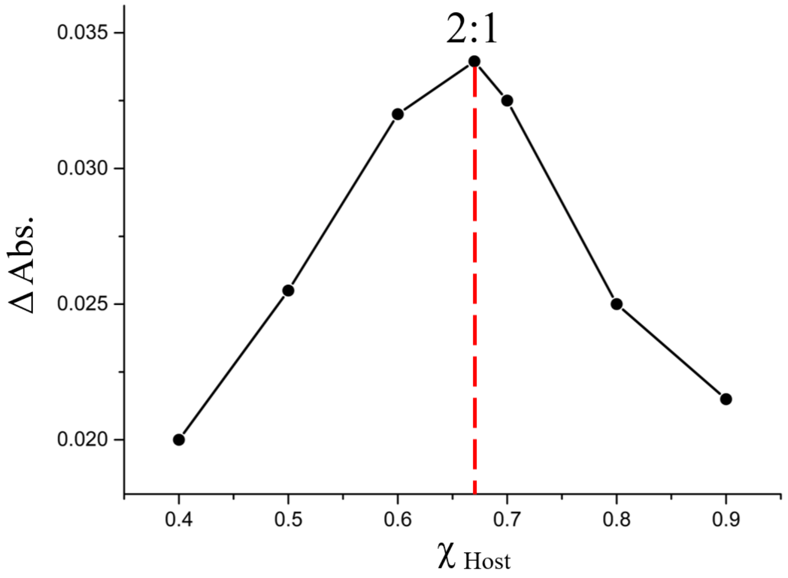

Figure S4 Job plot for determination of stoichiometry of **1a** and **G1** based on the absorbance at 400 nm in $CHCl_3$-CH_3CN (5:1, v/v, 298 K). [**1a**] + [**G1**] = 50 μM.

HRMS spectrum of G1⊂1a₂

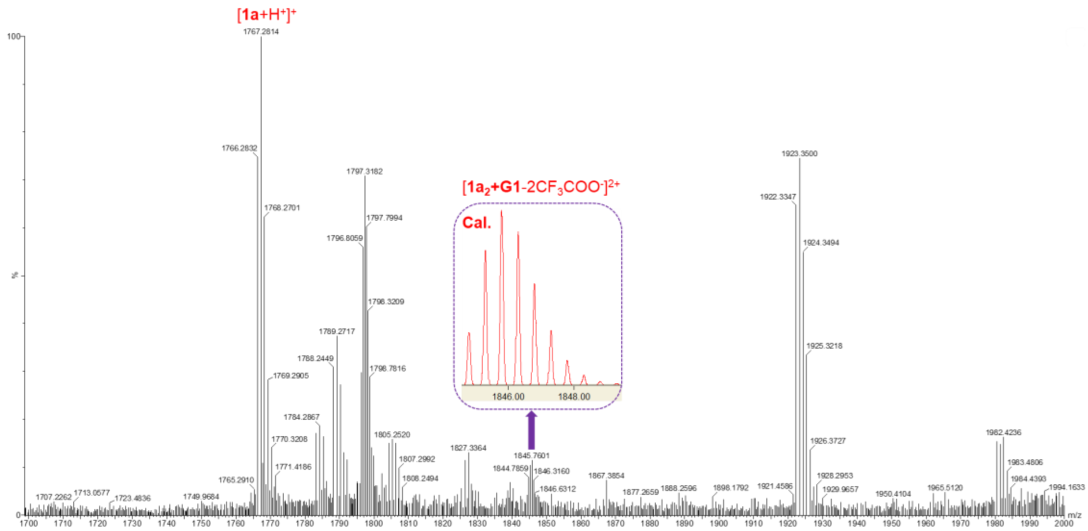

Figure S5 Partial HRMS spectrum of **G1**⊂**1a₂** complex in $CHCl_3$-CH_3CN (5:1, v/v). HRMS (ESI) calculated for [**1a₂**+**G1**-2CF_3COO^-]$^{2+}$, m/z, 1845.2919, found 1845.3367.

2D NOESY spectrum for G1⊂1a₂

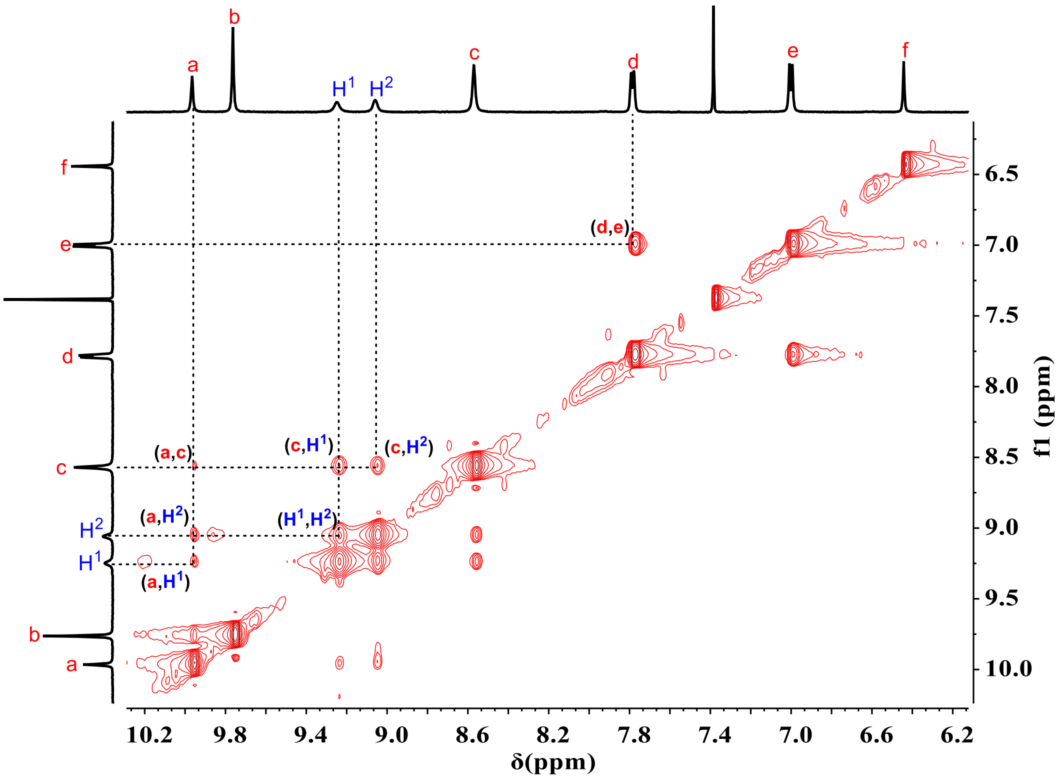

Figure S6 Expanded 2D-NOESY spectrum of **G1⊂1a**$_2$ ([**1a**] = 10 mM, [**G1**] = 5 mM, **1a**:**G1** = 2:1) (600 MHz, $CDCl_3$-CD_3CN, 5:1, v/v, 298 K, mixing time=0.4 s).

UV-vis titration experiments

To determine the binding constant (K_a) of macrocycle **1a** and guest **G1**, UV-vis titration experiments were performed in $CHCl_3$-CH_3CN (5:1, v/v, 298 K) at a constant concentration of **1a** (50 μM) and varying concentration of **G1**. For the titration, at least 20 data points were collected. Binding constant was calculated by a global fitting analysis according to a 2:1 binding model using the website *(http://supramolecular.org/)*.

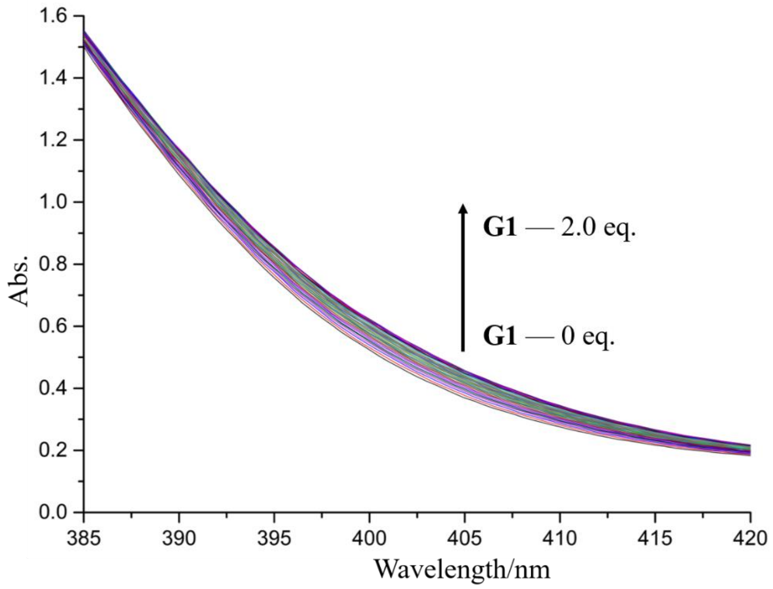

Figure S7 Stacked UV-vis spectra of **1a** (50 μM) titrated with **G1** from 0 equiv. to 2.0 equiv. in $CHCl_3$-CH_3CN (5:1, v/v, 298 K).

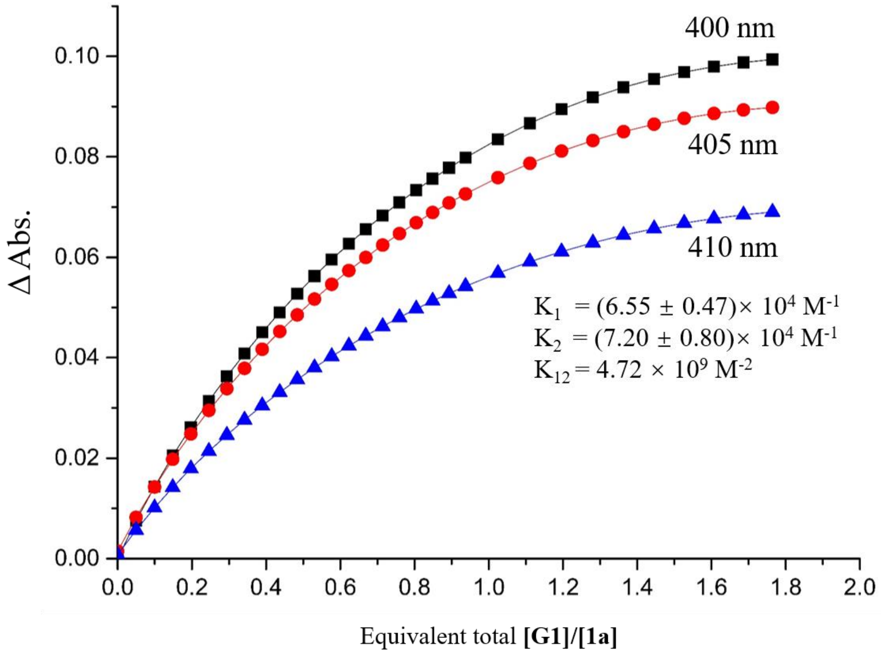

Figure S8 Curve fitting of the binding constant of **G1⊂1a₂** in $CHCl_3$-CH_3CN (5:1, v/v, 298 K). The reported binding constant is the average value based on fitting of the absorbance at 400 nm, 405 nm, and 410 nm.

Host-guest chemistry of 1a and G2

^{1}H NMR spectra for 1a and G2 interactions

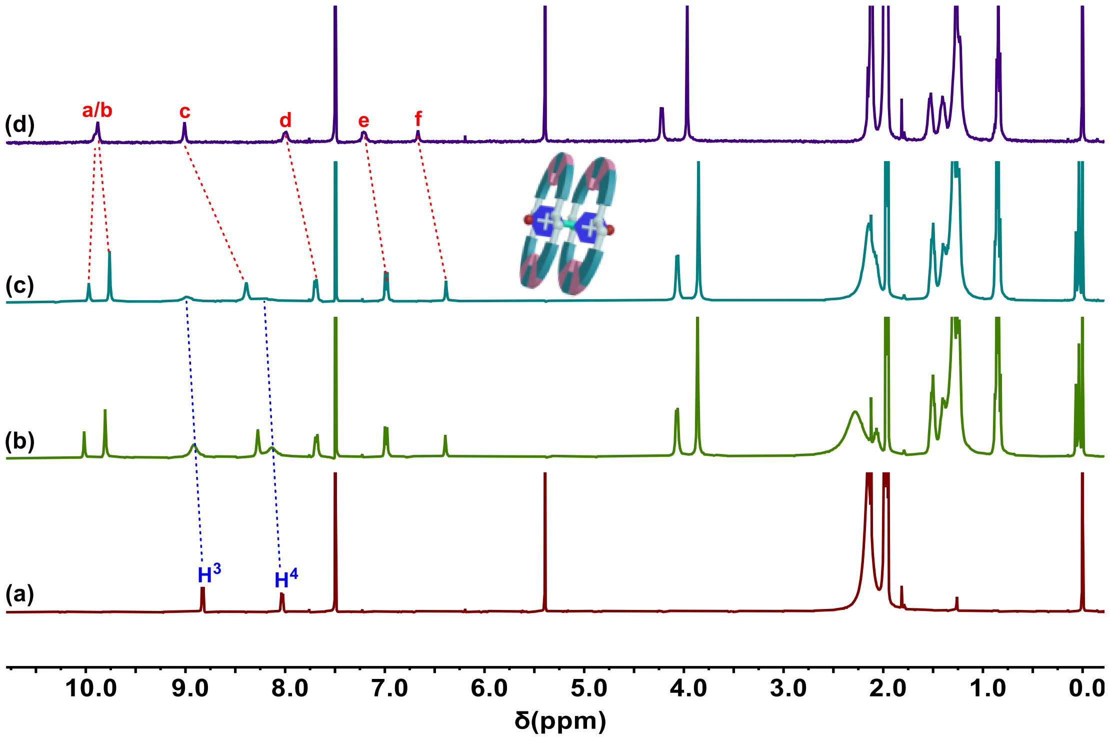

Figure S9 ^{1}H NMR spectra (400 MHz, $CDCl_3$-CD_3CN, 1:1, v/v, 298 K) of (a) **G2** (1.0 mM), (b) **1a** and **G2** (1.0 mM for each), (c) **1a** (2.0 mM) and **G2** (1.0 mM) and (d) **1a** (1.0 mM).

2D NOESY spectrum for G2⊂1a$_2$

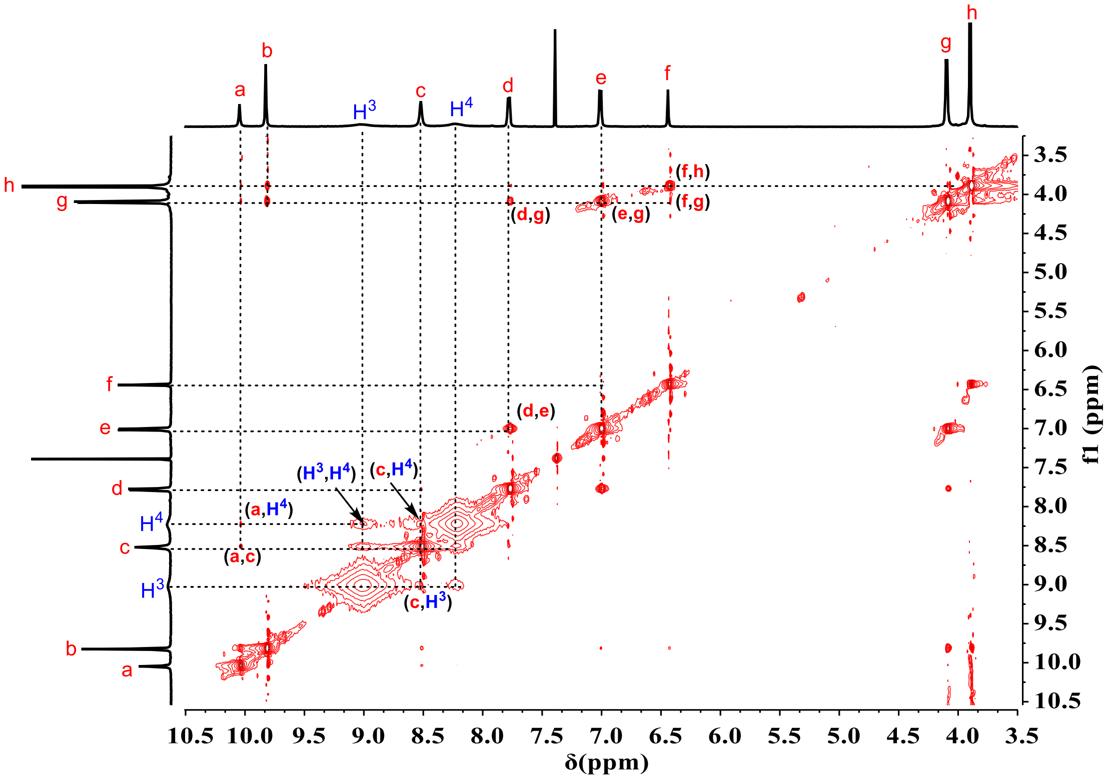

Figure S10 Expanded 2D-NOESY spectrum of **G2⊂1a$_2$** ([**1a**] = 10 mM, [**G2**] = 5 mM, **1a:G2** = 2:1) (600 MHz, $CDCl_3$-CD_3CN, 1:1, v/v, 298 K, mixing time = 0.4 s).

Job plot for determination of stoichiometry

Because of the poor solubility of host **1a** in $CHCl_3$-CD_3CN (1:1, v/v), host **1b** was used for Job plot.

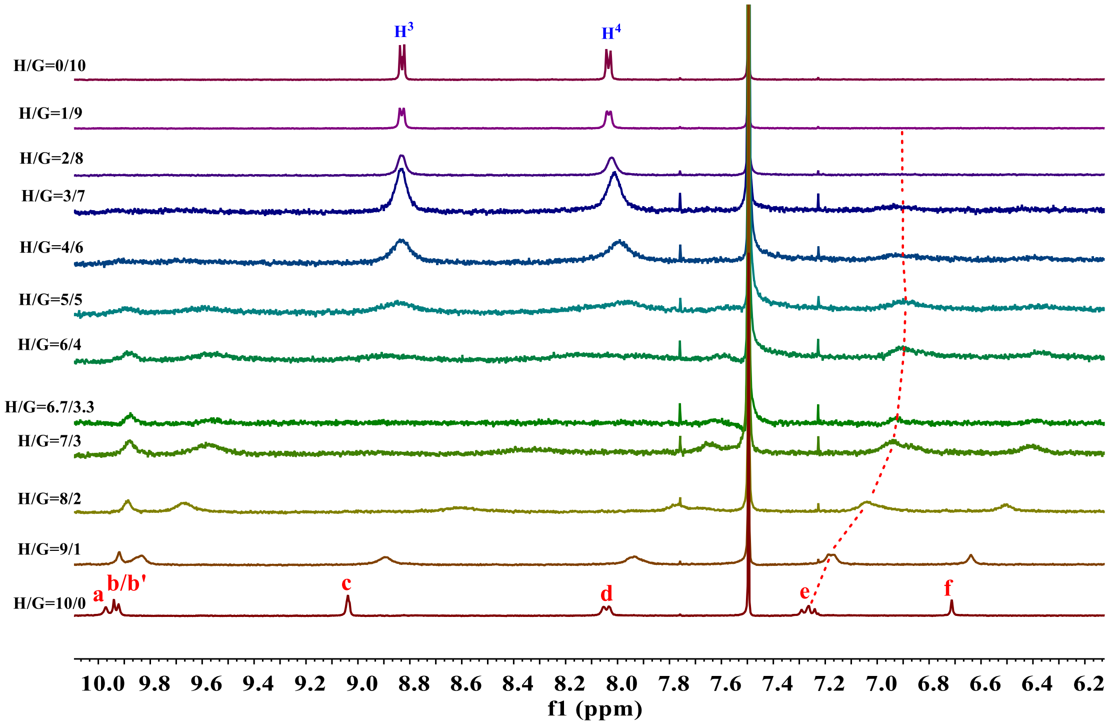

Figure S11 Partial stacked ^{1}H-NMR spectra ($CDCl_3$-CD_3CN, 1:1, v/v, 400 MHz, 298 K) utilized in Job plot between **1b** and **G2**. [**1b**]+[**G2**]=1 mM.

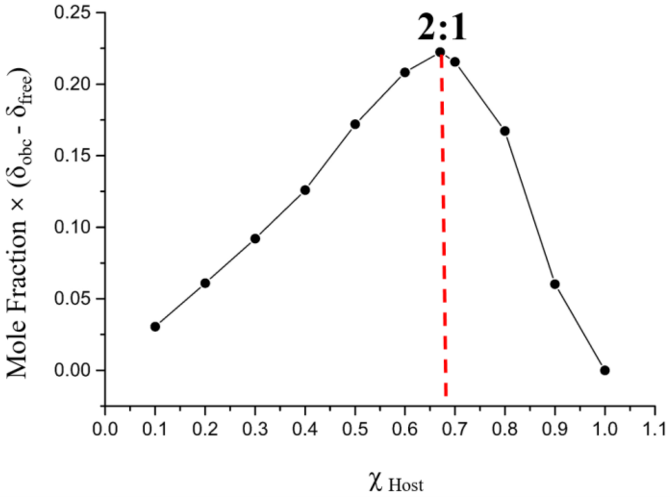

Figure S12 Job plot showing a peak maximum was reached around 0.67 corresponding to the formation of a 2:1 host-guest complex between **1b** and **G2**. The plot was constructed from data shown in **Figure S11**.

HRMS spectrum of G2⊂$1a_2$

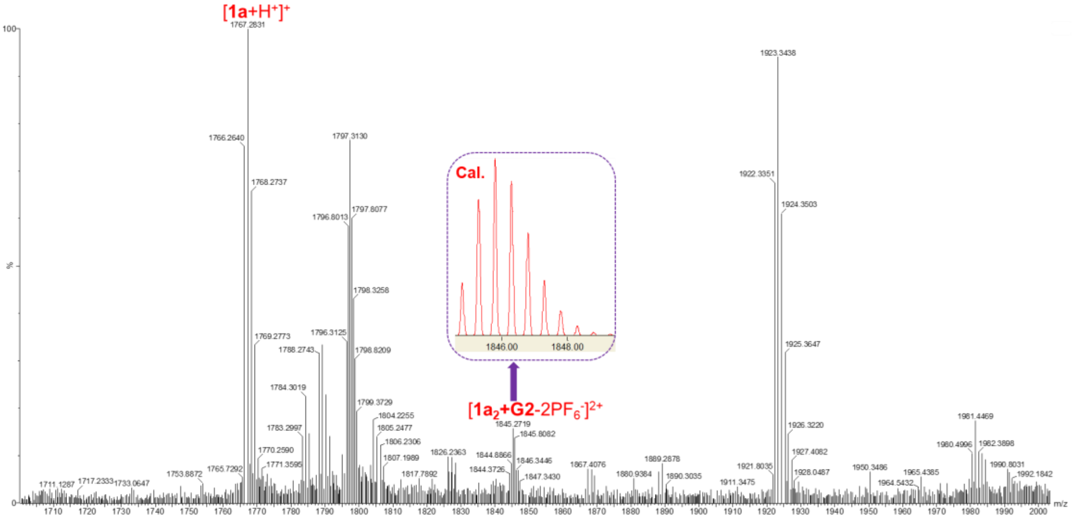

Figure S13 Partial HRMS spectrum of **G2**⊂**1a**$_2$ complex in $CHCl_3$-CH_3CN (1:1, v/v). HRMS (ESI) calculated for [**1a**$_2$+**G2**-2PF_6^-]$^{2+}$, m/z, 1845.2919, found 1845.2719.

UV-vis titration experiments

To determine the binding constant (K_a) of macrocycle **1a** and guest **G2**, UV-vis titration experiments were performed in $CHCl_3$-CH_3CN (1:1, v/v, 298 K) at a constant concentration of **1a** (50 μM) and varying concentration of **G2**. For the titration, at least 20 data points were collected. Binding constant was calculated by a global fitting analysis according to a 2:1 binding model using the website *(http://supramolecular.org/)*.

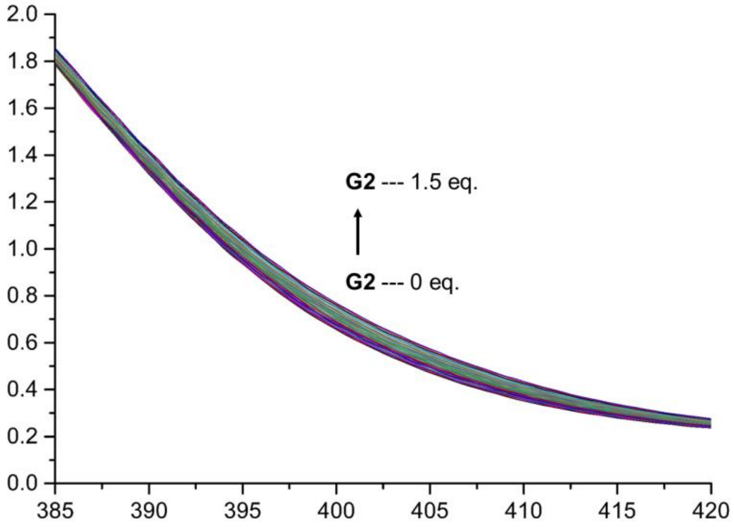

Figure S14 Stacked UV-vis spectra of **1a** (50 μM) titrated with **G2** from 0 equiv. to 1.5 equiv. in $CHCl_3$-CH_3CN (1:1, v/v 298 K).

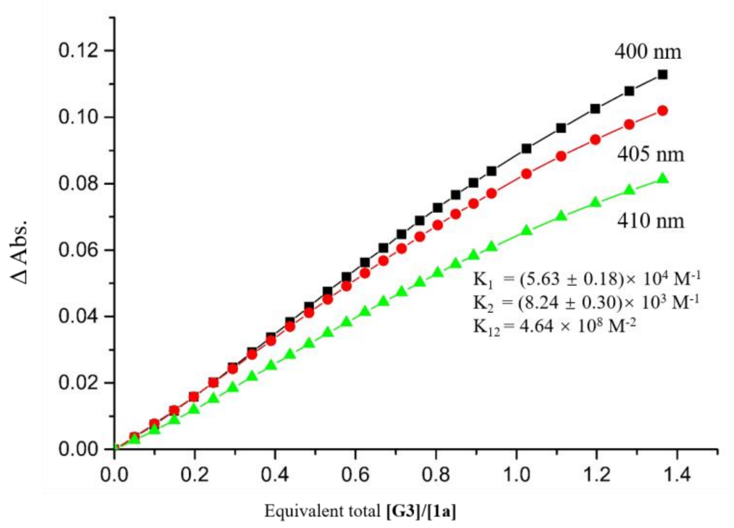

Figure S15 Curve fitting of the binding constant of **G2⊂1a₂** in $CHCl_3$-CH_3CN (1:1, v/v, 298 K). The reported binding constant is the average value based on fitting of the absorbance at 400 nm, 405 nm and 410 nm.

^{1}H NMR spectra of host-guest complexes

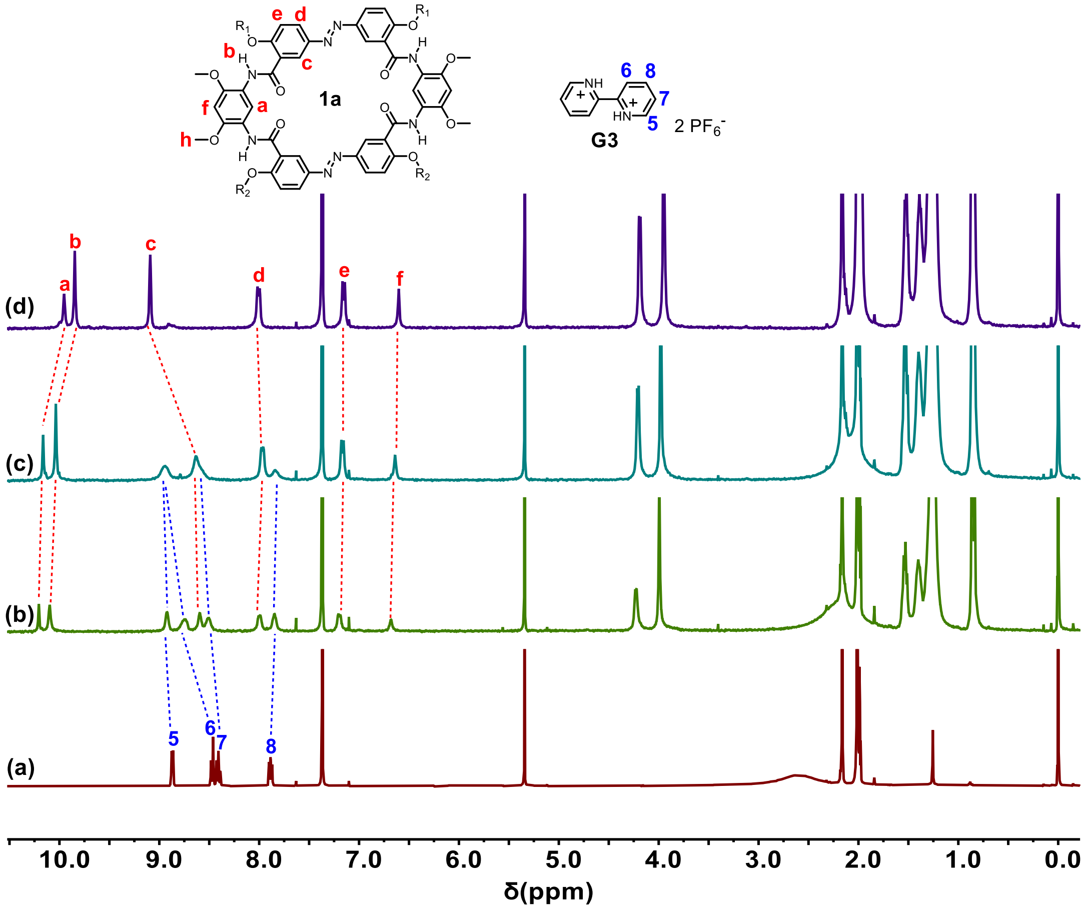

Figure S16 ^{1}H NMR spectra ($CDCl_3$-CD_3CN, 5:1, v/v, 400 MHz, 298 K) of (a) free **G3** (1.0 mM), (b) **1a** (1 mM) and **G3** (1 mM), (c) **1a** (2 mM) and **G3** (1 mM), and (d) free **1a** (1.0 mM).

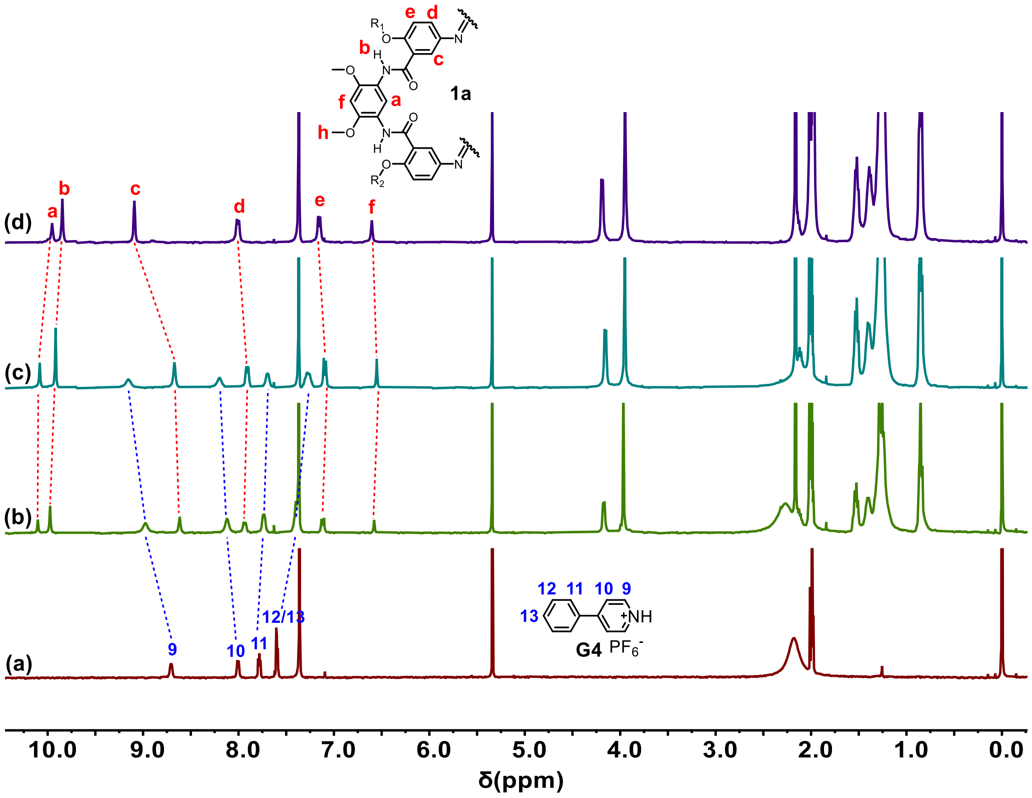

Figure S17 ^{1}H NMR spectra (CDCl3-CD3CN, 5:1, v/v, 400 MHz, 298 K) of (a) free **G4** (2.0 mM), (b) **1a** (1 mM) and **G4** (2 mM), (c) **1a** (2 mM) and **G4** (2 mM), and (d) free **1a** (2.0 mM).

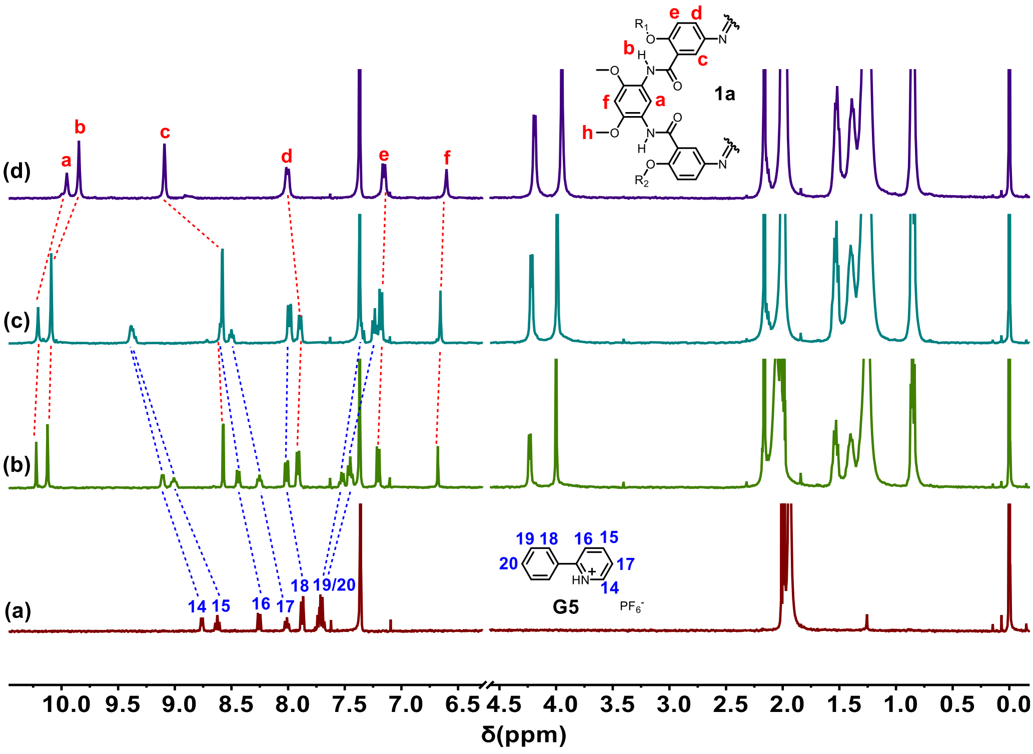

Figure S18 ^{1}H NMR spectra (CDCl3-CD3CN, 5:1, v/v, 400 MHz, 298 K) of (a) free **G5** (2.0 mM), (b) **1a** (1 mM) and **G5** (2 mM), (c) **1a** (2 mM) and **G5** (2 mM), and (d) free **1a** (2.0 mM).

Job plot for determination of stoichiometry

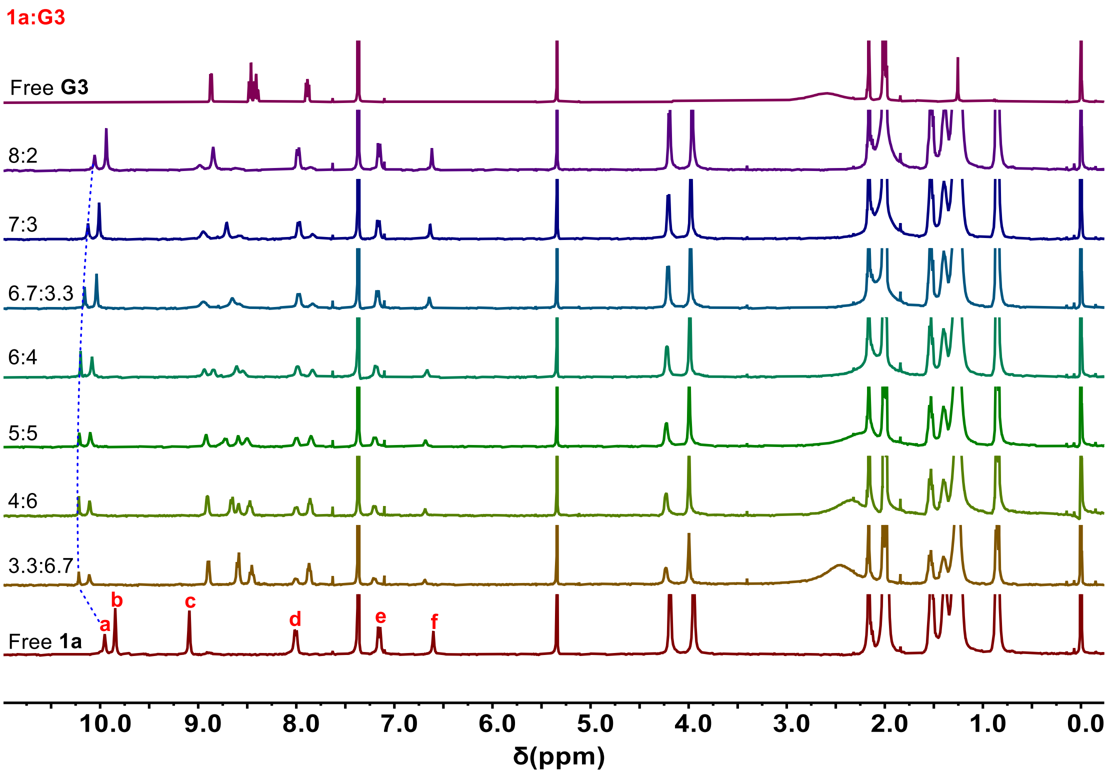

Figure S19 Stack ^{1}H NMR spectra ($CDCl_3$-CD_3CN, 5:1, v/v, 400 MHz, 298 K) utilized in the job plot between **1a** and **G3**. [**1a**] + [**G3**] = 2.0 mM.

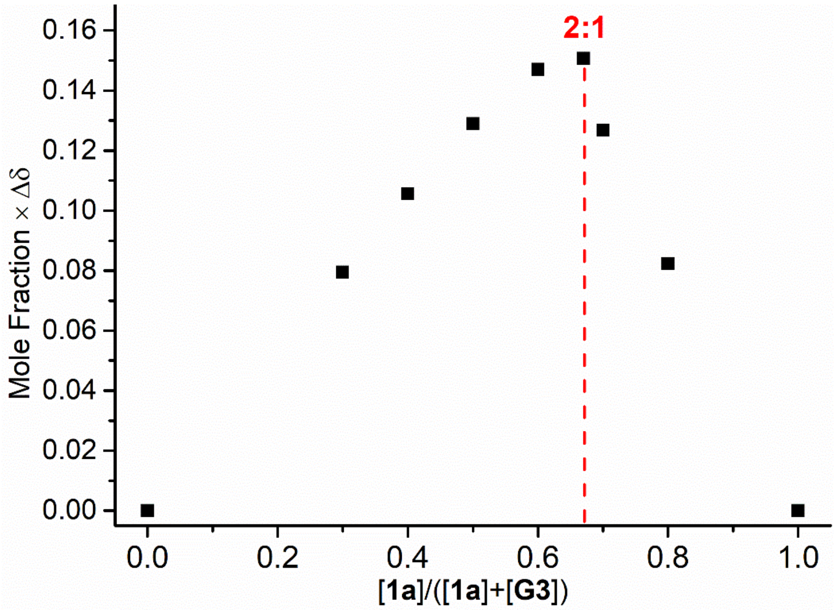

Figure S20 Job plot showing a peak maximum was reached around 0.67 corresponding to the formation of a 2:1 host-guest complex between **1a** and **G3**. The plot was constructed from data shown in **Figure S19**.

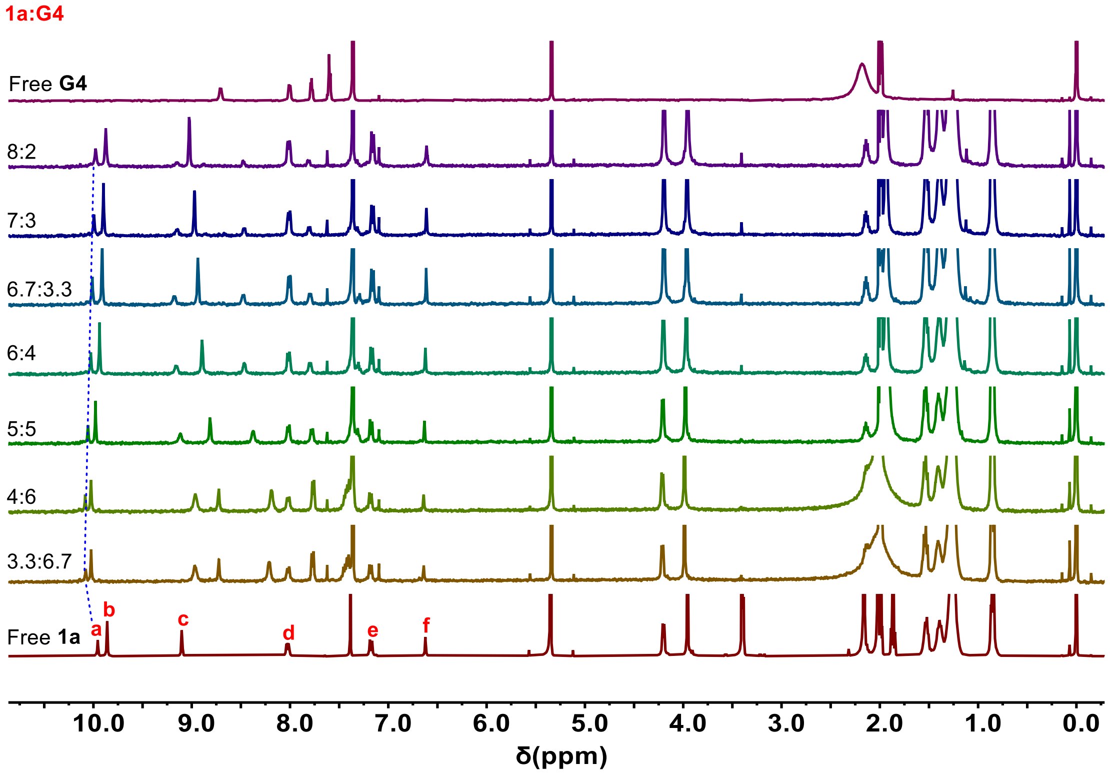

Figure S21 Stack ^{1}H NMR spectra ($CDCl_3$-CD_3CN, 5:1, v/v, 400 MHz, 298 K) utilized in the job plot between **1a** and **G4**. [**1a**] + [**G4**] = 2.0 mM.

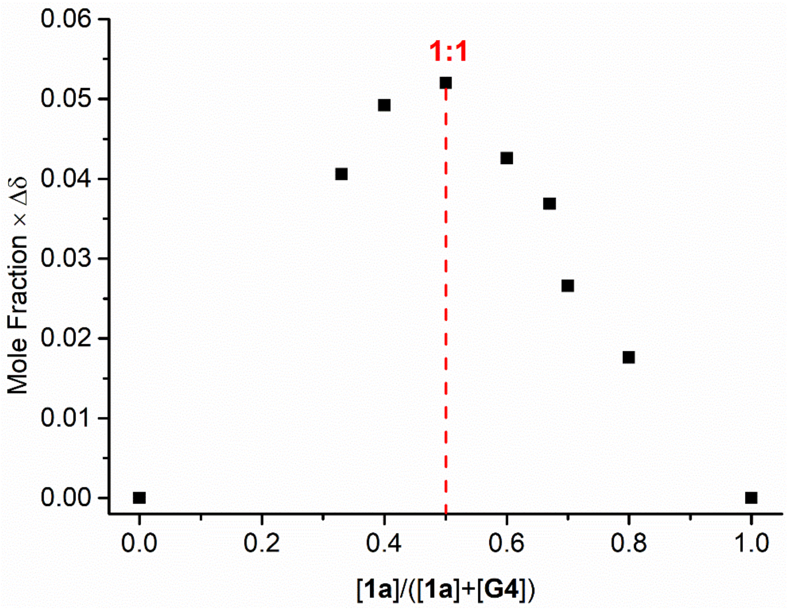

Figure S22 Job plot showing a peak maximum was reached around 0.5 corresponding to the formation of a 1:1 host-guest complex between **1a** and **G4**. The plot was constructed from data shown in **Figure S21**.

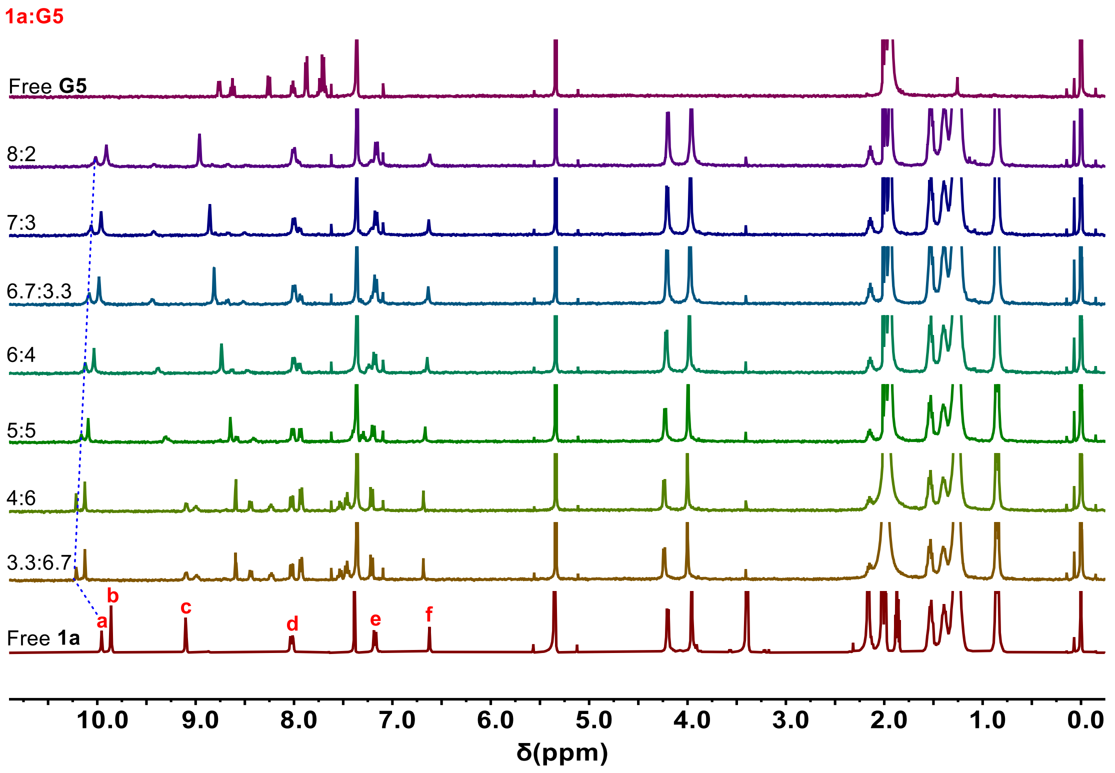

Figure S23 Stack ^{1}H NMR spectra ($CDCl_3$-CD_3CN, 5:1, v/v, 400 MHz, 298 K) utilized in the job plot between **1a** and **G5**. [**1a**] + [**G5**] = 2.0 mM.

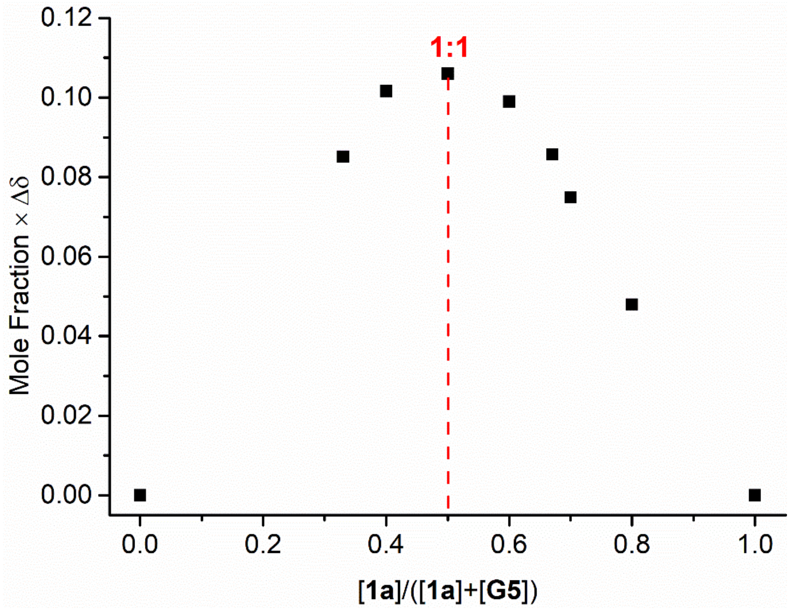

Figure S24 Job plot showing a peak maximum was reached around 0.5 corresponding to the formation of a 1:1 host-guest complex between **1a** and **G5**. The plot was constructed from data shown in **Figure S23**.

HRMS spectra of complexes

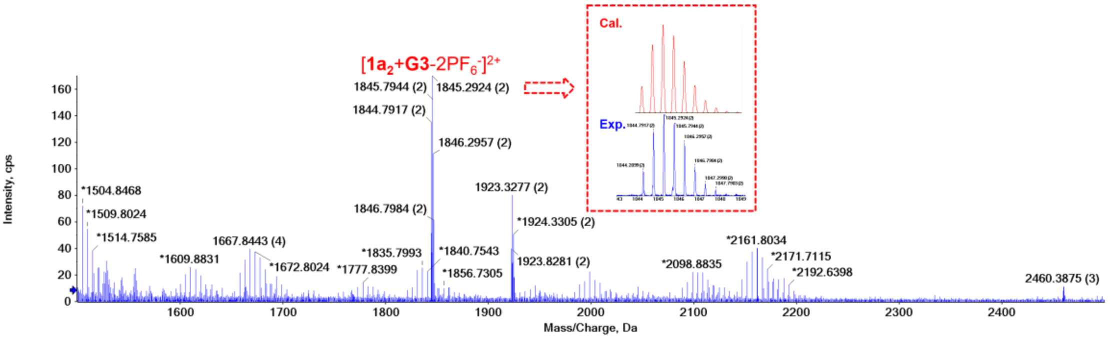

Figure S25 Partial HRMS spectrum of **G3**⊂**1a$_2$** complex in $CHCl_3$-CH_3CN (5:1, v/v). HRMS (ESI) calculated for [**1a$_2$**+**G3**-2PF_6^-]$^{2+}$, m/z, 1845.2919, found 1845.2924. Inserted images show the calculated (top, red) and experimented (bottom, blue) isotope patterns.

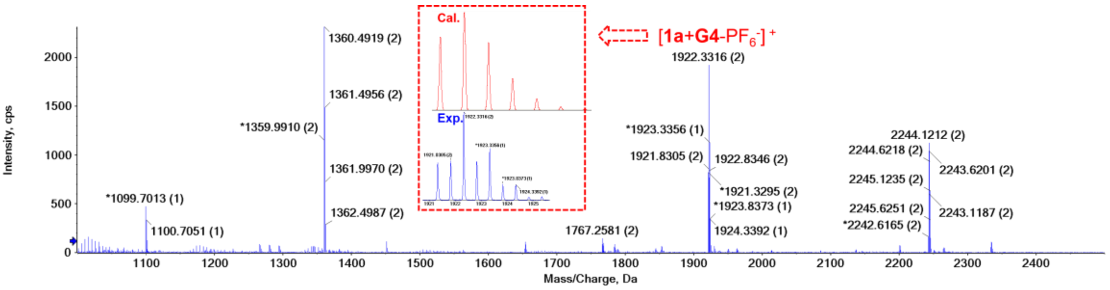

Figure S26 Partial HRMS spectrum of **G4**⊂**1a** complex in $CHCl_3$-CH_3CN (5:1, v/v). HRMS (ESI) calculated for [**1a**+**G4**-PF_6^-]$^+$, m/z, 1922.3310, found 1922.3316. Inserted images show the calculated (top, red) and experimented (bottom, blue) isotope patterns.

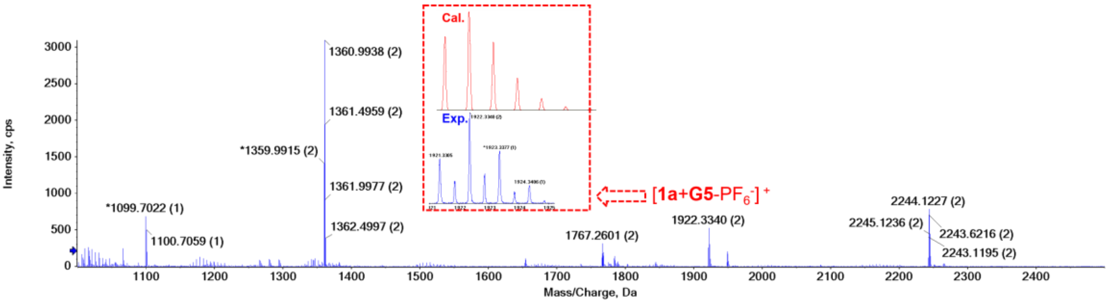

Figure S27 Partial HRMS spectrum of **G5**⊂**1a** complex in $CHCl_3$-CH_3CN (5:1, v/v). HRMS (ESI) calculated for [**1a**+**G5**-PF_6^-]$^+$, m/z, 1922.3310, found 1922.3340. Inserted images show the calculated (top, red) and experimented (bottom, blue) isotope patterns.

UV-vis titration experiments

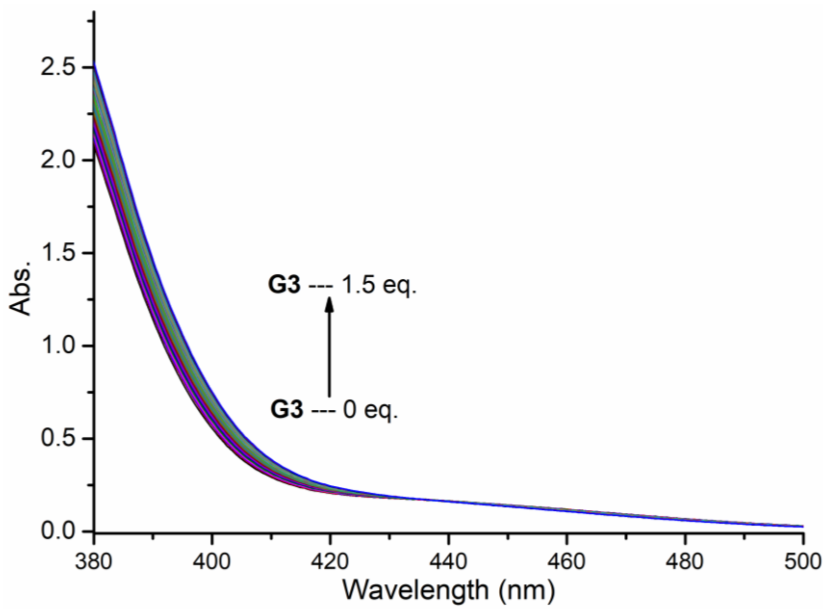

Figure S28 Stacked UV-vis spectra of **1a** (50 μM) titrated with **G3** from 0 equiv. to 1.5 equiv. in $CHCl_3$-CH_3CN (5:1, v/v 298 K).

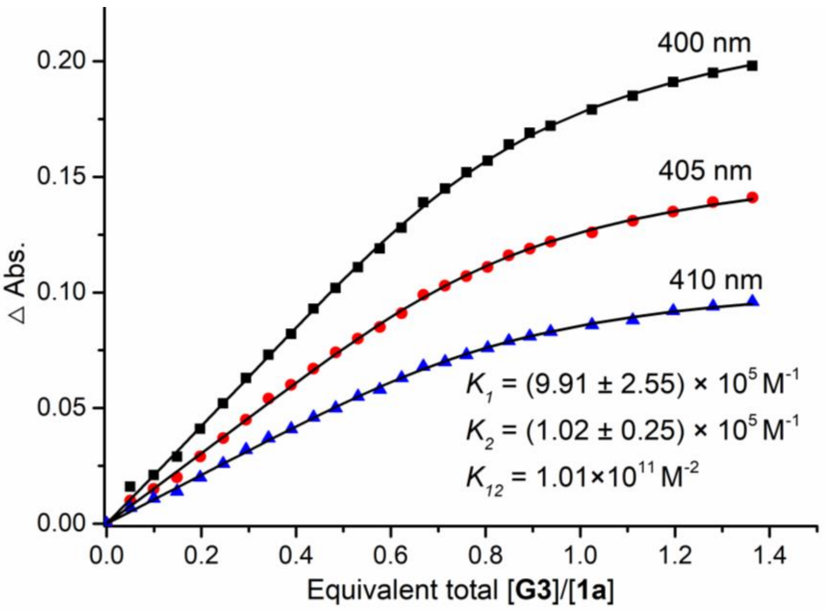

Figure S29 Curve fitting of the binding constant of **G3⊂1a₂** in $CHCl_3$-CH_3CN (5:1, v/v, 298 K). The reported binding constant is the average value based on fitting of the absorbance at 400 nm, 405 nm and 410 nm.

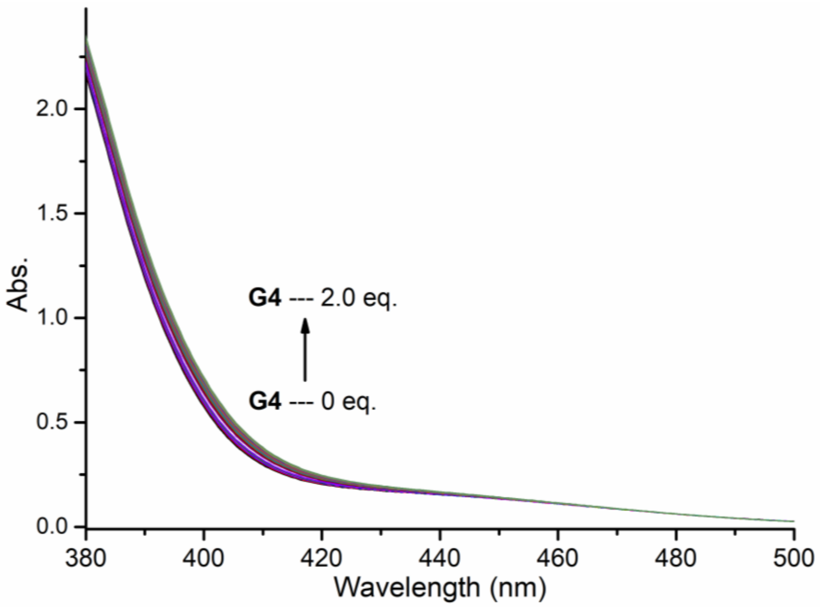

Figure S30 Stacked UV-vis spectra of **1a** (50 μM) titrated with **G4** from 0 equiv. to 2.0 equiv. in $CHCl_3$-CH_3CN (5:1, v/v 298 K)**.**

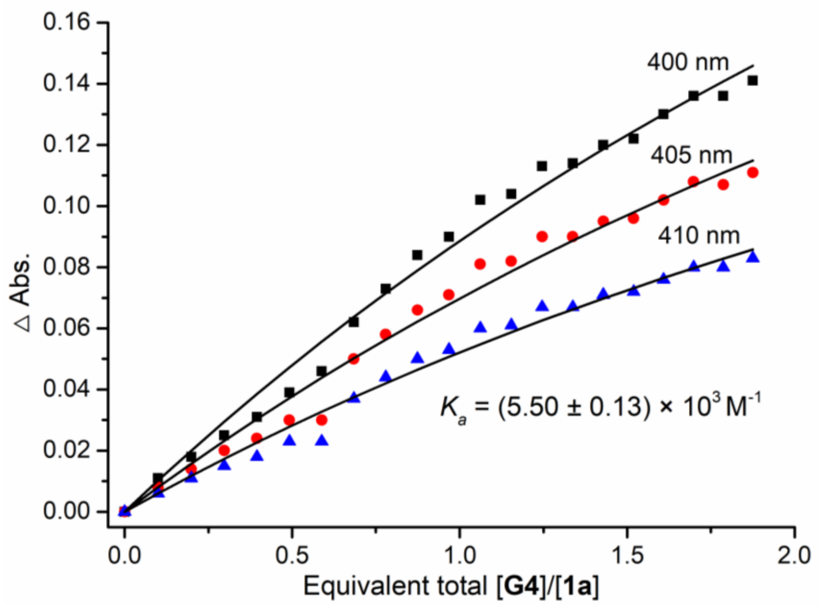

Figure S31 Curve fitting of the binding constant of **G4**⊂**1a** in $CHCl_3$-CH_3CN (5:1, v/v, 298 K). The reported binding constant is the average value based on fitting of the absorbance at 400 nm, 405 nm and 410 nm.

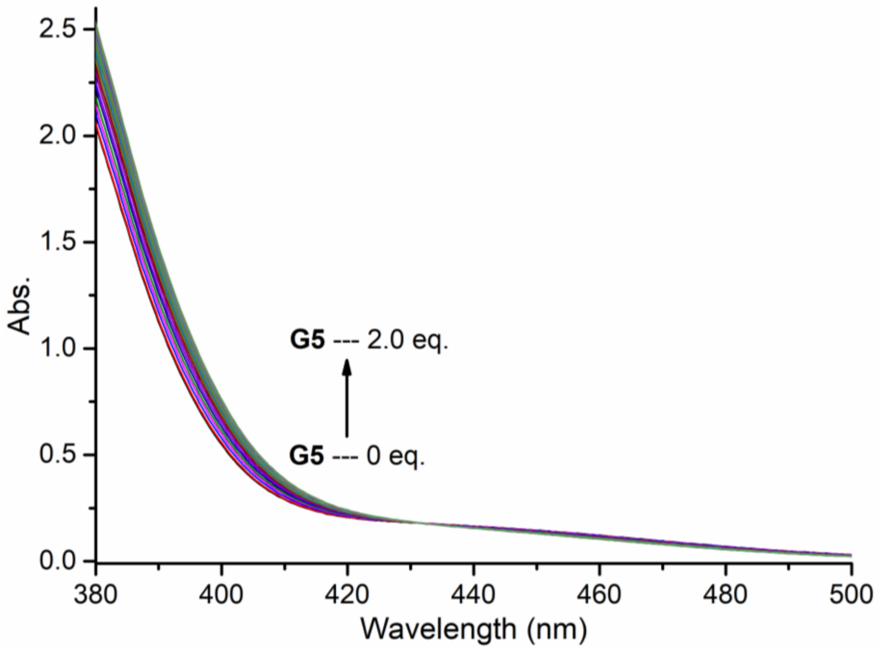

Figure S32 Stacked UV-vis spectra of **1a** (50 μM) titrated with **G5** from 0 equiv. to 2.0 equiv. in $CHCl_3$-CH_3CN (5:1, v/v 298 K).

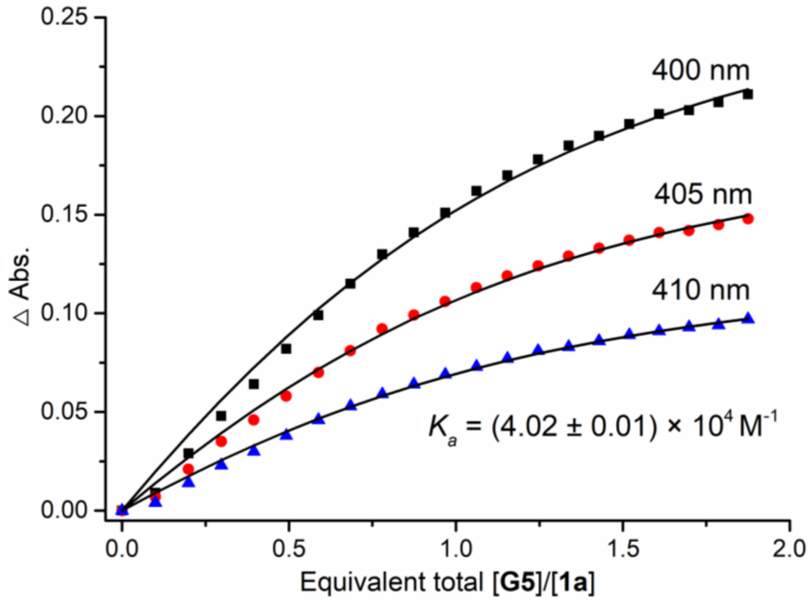

Figure S33 Curve fitting of the binding constant of **G5**⊂**1a** in $CHCl_3$-CH_3CN (5:1, v/v, 298 K). The reported binding constant is the average value based on fitting of the absorbance at 400 nm, 405 nm and 410 nm.

Photo-responsiveness of host 1a

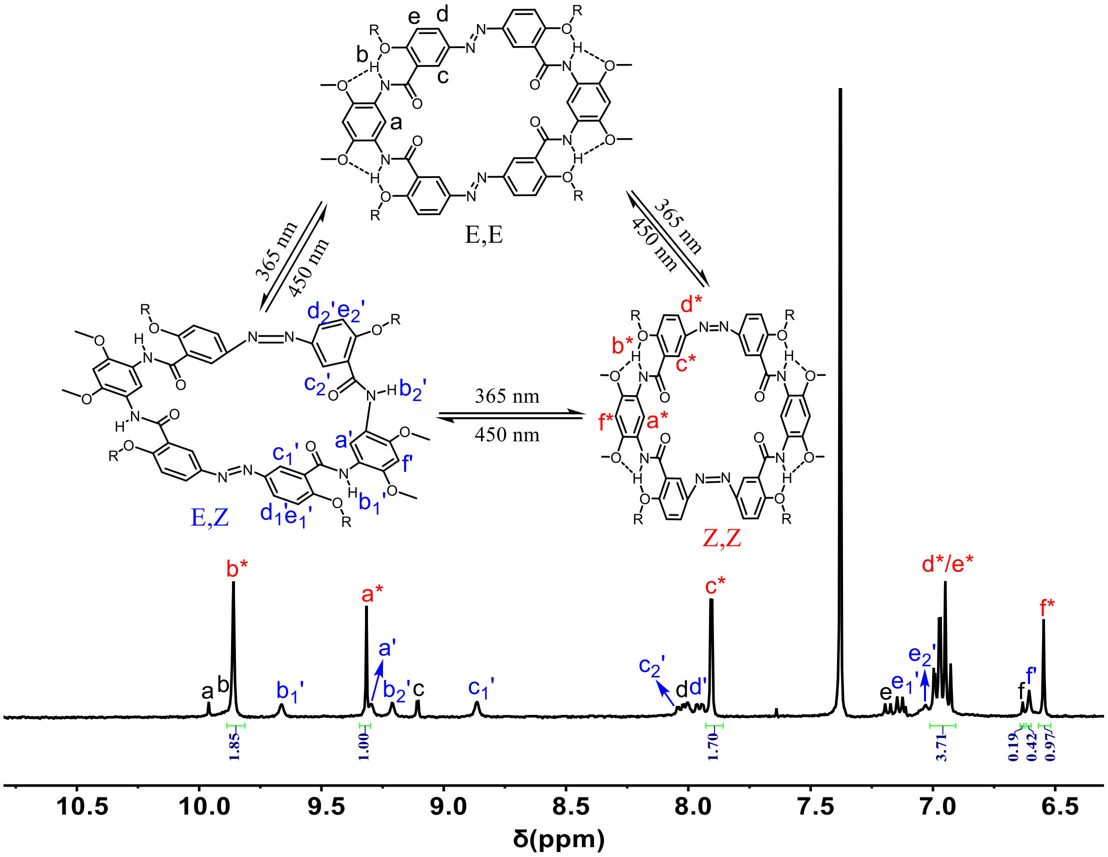

Figure S34 Partial ^{1}H-NMR spectrum (400 MHz, $CDCl_3$-CD_3CN, 5:1, v/v, 298 K) of **1a** under UV irradiation for 25 min.

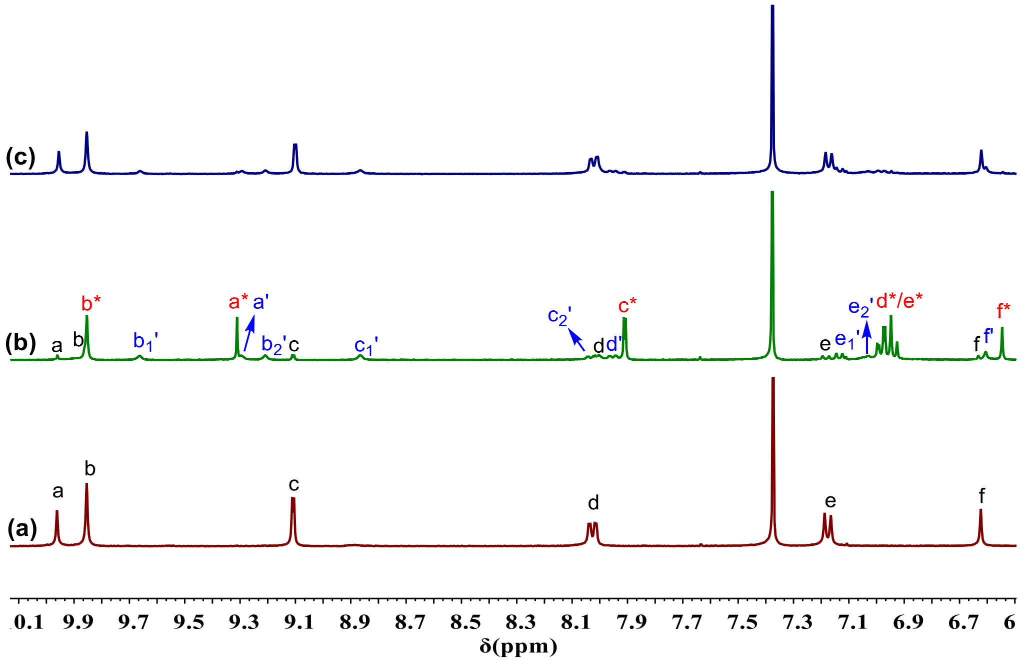

Figure S35 Stacked ^{1}H-NMR spectra (400 MHz, $CDCl_3$-CD_3CN, 5:1, v/v, 298 K) of **1a** (a) before and (b) after UV irradiation for 25 min, (c) then under blue light irradiation of (b).

Photo-responsive binding and release of the guests in G1⊂1a₂

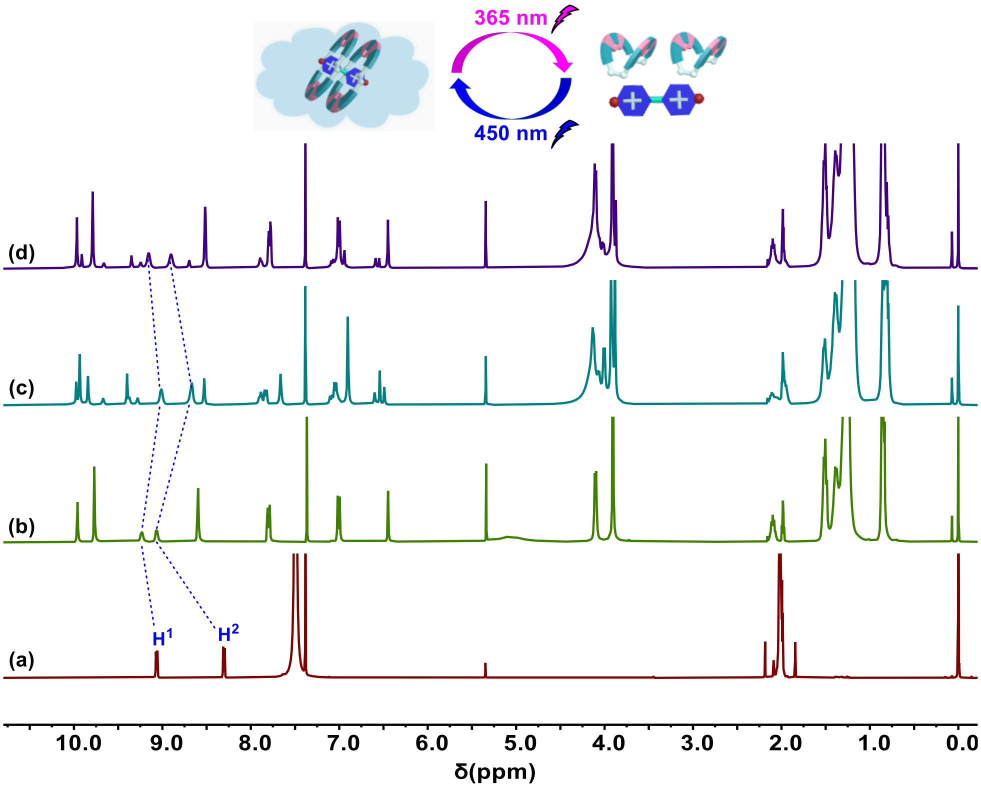

Figure S36 ^{1}H-NMR spectra (400 MHz, $CDCl_3$-CD_3CN, 5:1, v/v, 298 K) of (a) free **G1**, (b) complex **G1⊂1a₂**, (c) complex **G1⊂1a₂** after UV irradiation, (d) under blue light irradiation of (c).

Photo-responsive of host-gust complex G2⊂1a₂, G3⊂1a₂, G4⊂1a, and G5⊂1a

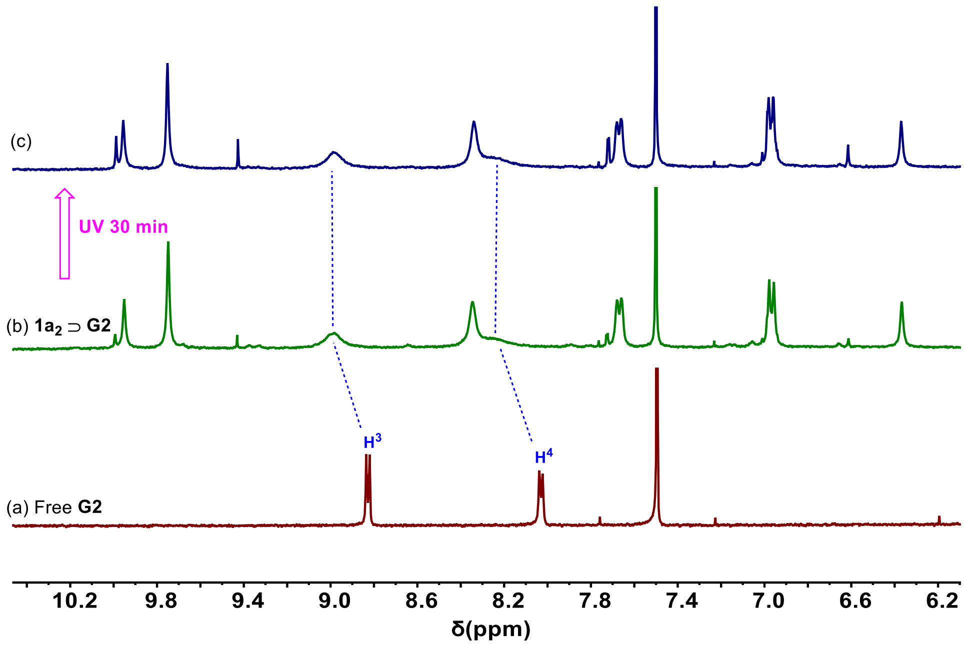

Figure S37 Partial ^{1}H NMR spectra (400 MHz, $CDCl_3$-CD_3CN, 1:1, v/v, 298 K) of (a) free **G2**, (b) **1a:G2**=2:1 before UV irradiation, and (c) **1a:G2**=2:1 after UV irradiation.

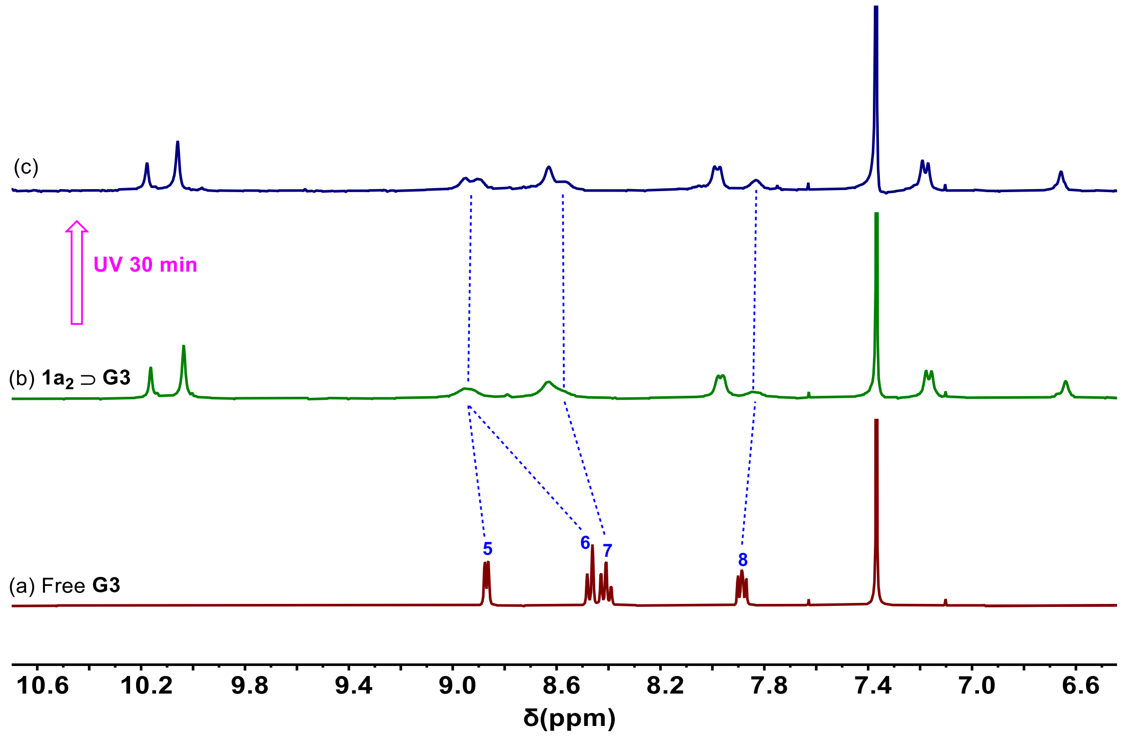

Figure S38 Partial ^{1}H NMR spectra (400 MHz, $CDCl_3$-CD_3CN, 5:1, v/v, 298 K) of (a) free **G3**, (b) **1a:G3**=2:1 before UV irradiation, and (c) **1a:G3**=2:1 after UV irradiation.

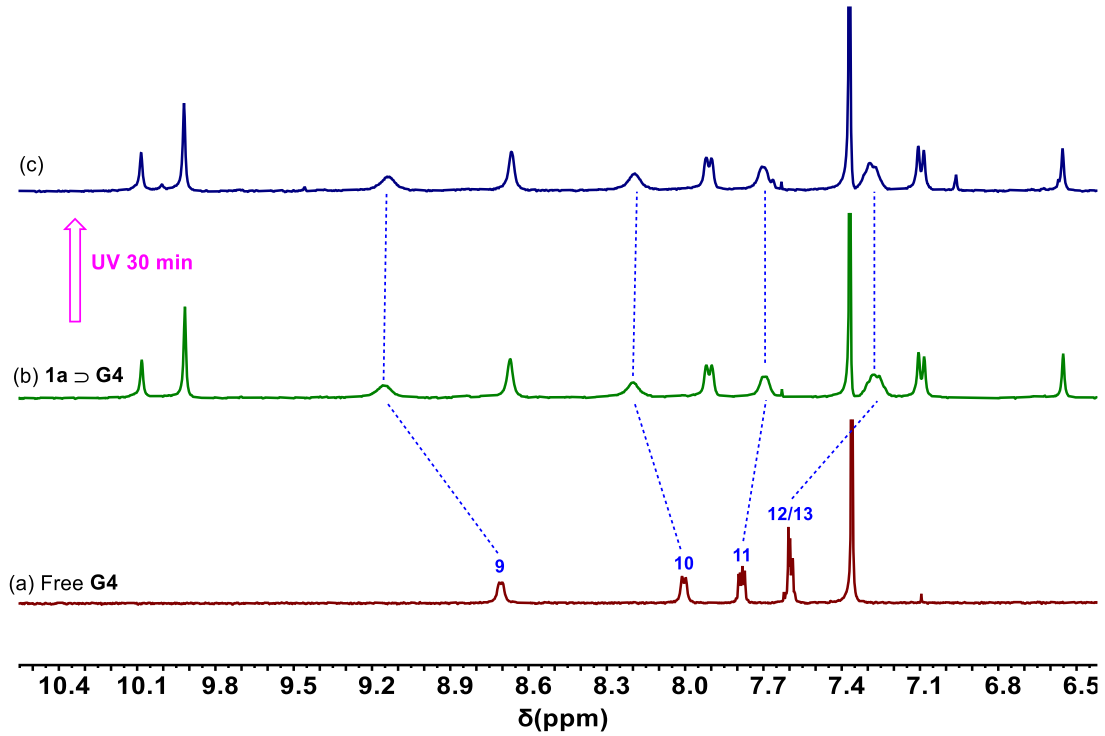

Figure S39 Partial ^{1}H NMR spectra (400 MHz, $CDCl_3$-CD_3CN, 5:1, v/v, 298 K) of (a) free **G4**, (b) **1a:G4**=2:1 before UV irradiation, and (c) **1a:G4**=2:1 after UV irradiation.

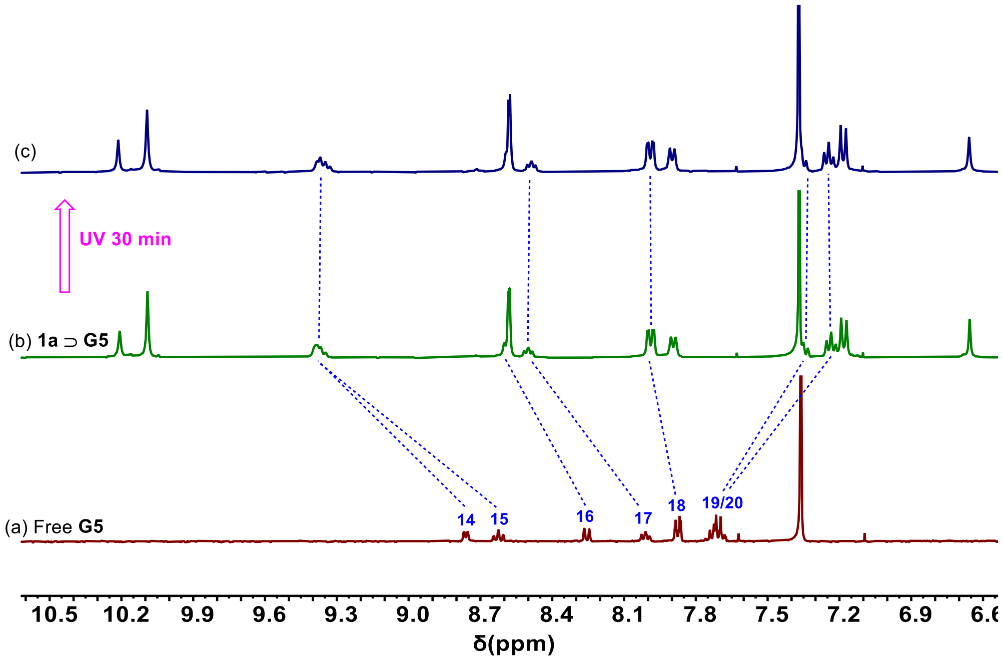

Figure S40 Partial ^{1}H NMR spectra (400 MHz, $CDCl_3$-CD_3CN, 5:1, v/v, 298 K) of (a) free **G5**, (b) **1a:G5**=2:1 before UV irradiation, and (c) **1a:G5**=2:1 after UV irradiation.

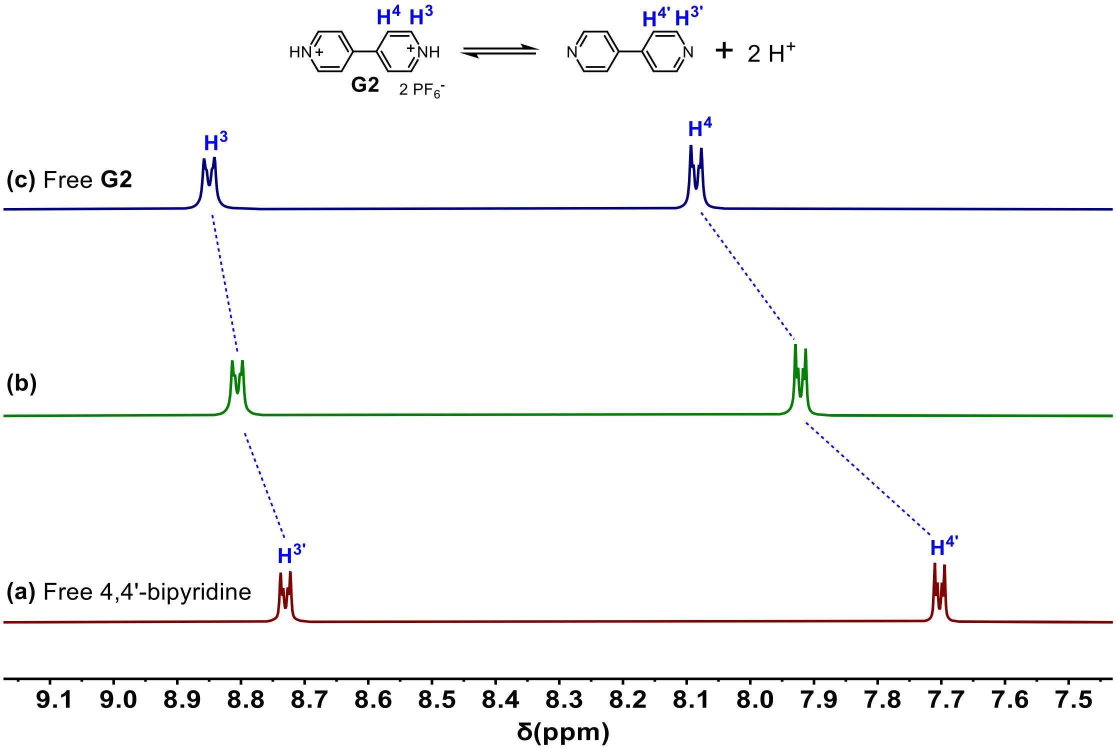

Figure S41 Partial ^{1}H NMR spectra (400 MHz, CD_3CN, 298 K) of (a) free 4,4'-bipyridine, (b) **G2** (1.0 mM) and 4,4'-bipyridine (1.0 mM), and (c) **G2** (1.0 mM).

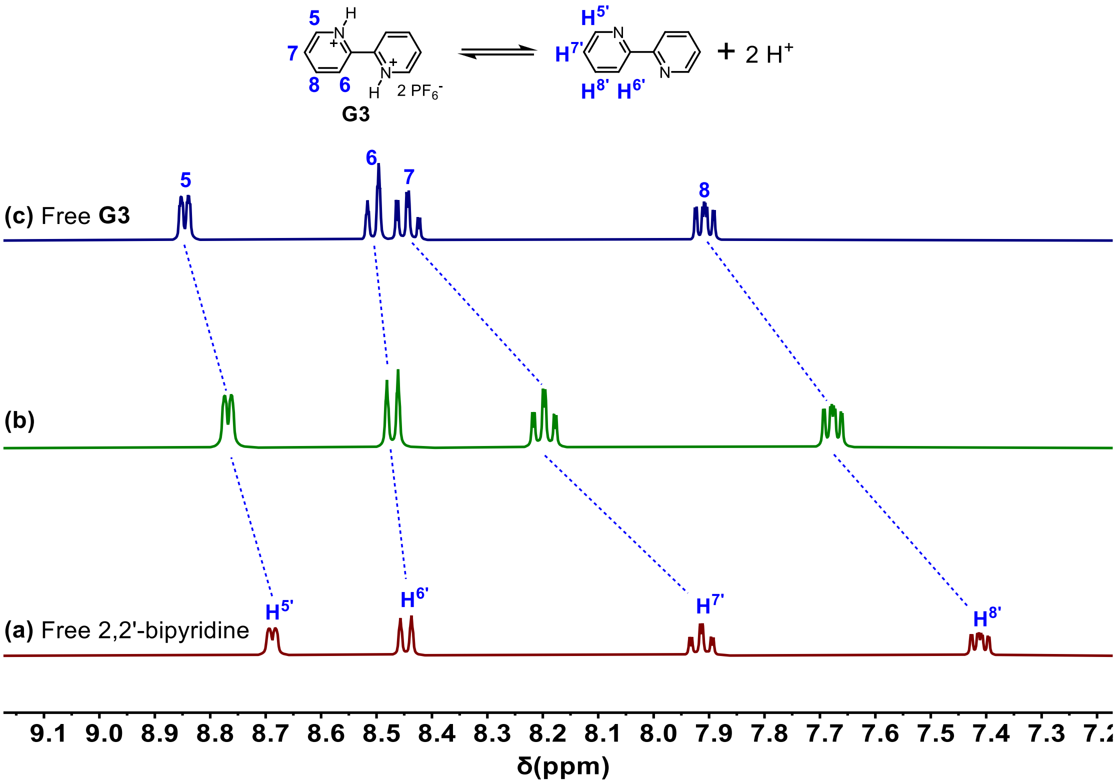

Figure S42 Partial ^{1}H NMR spectra (400 MHz, CD_3CN, 298 K) of (a) free 2,2'-bipyridine, (b) **G3** (1.0 mM) and 4,4'-bipyridine (1.0 mM), and (c) **G3** (1.0 mM).

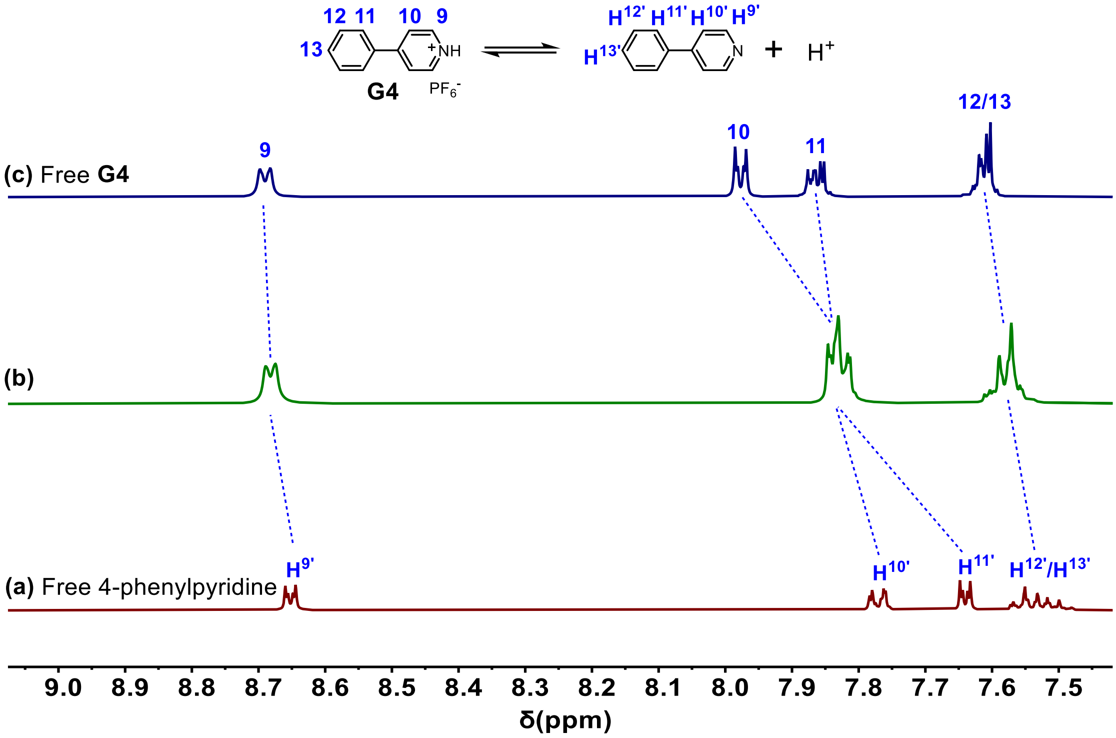

Figure S43 Partial ^{1}H NMR spectra (400 MHz, CD_3CN, 298 K) of (a) free 4-phenylpyridine, (b) **G4** (1.0 mM) and 4-phenylpyridine (1.0 mM), and (c) **G4** (1.0 mM).

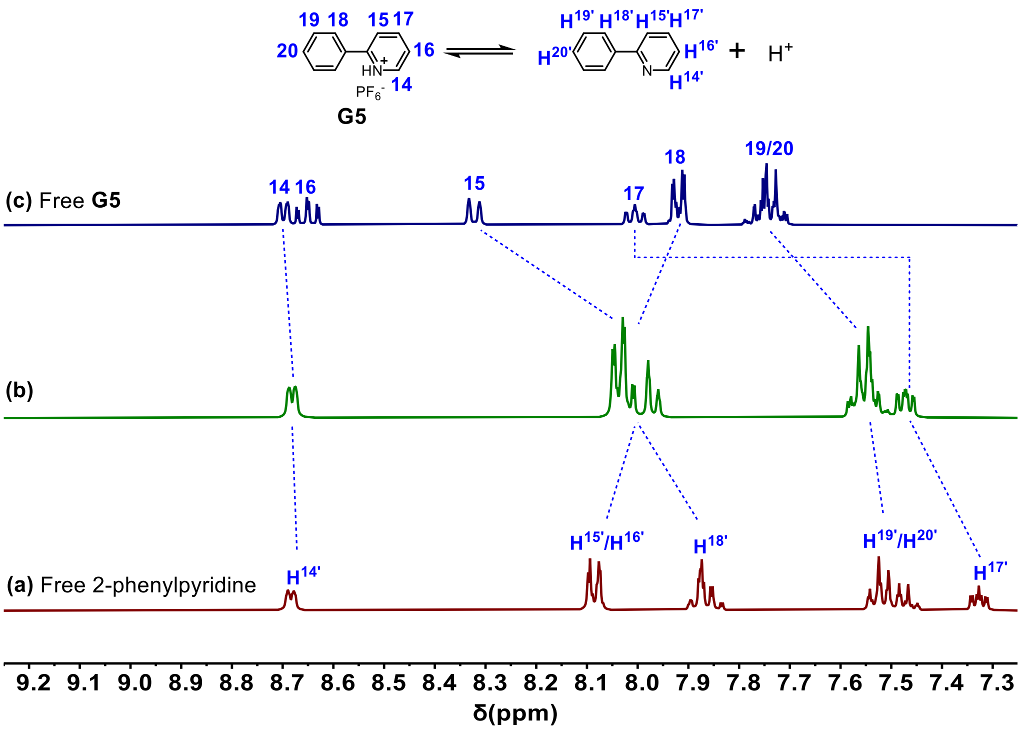

Figure S44 Partial ^{1}H NMR spectra (400 MHz, CD_3CN, 298 K) of (a) free 2-phenylpyridine, (b) **G5** (1.0 mM) and 2-phenylpyridine (1.0 mM), and (c) **G5** (1.0 mM).

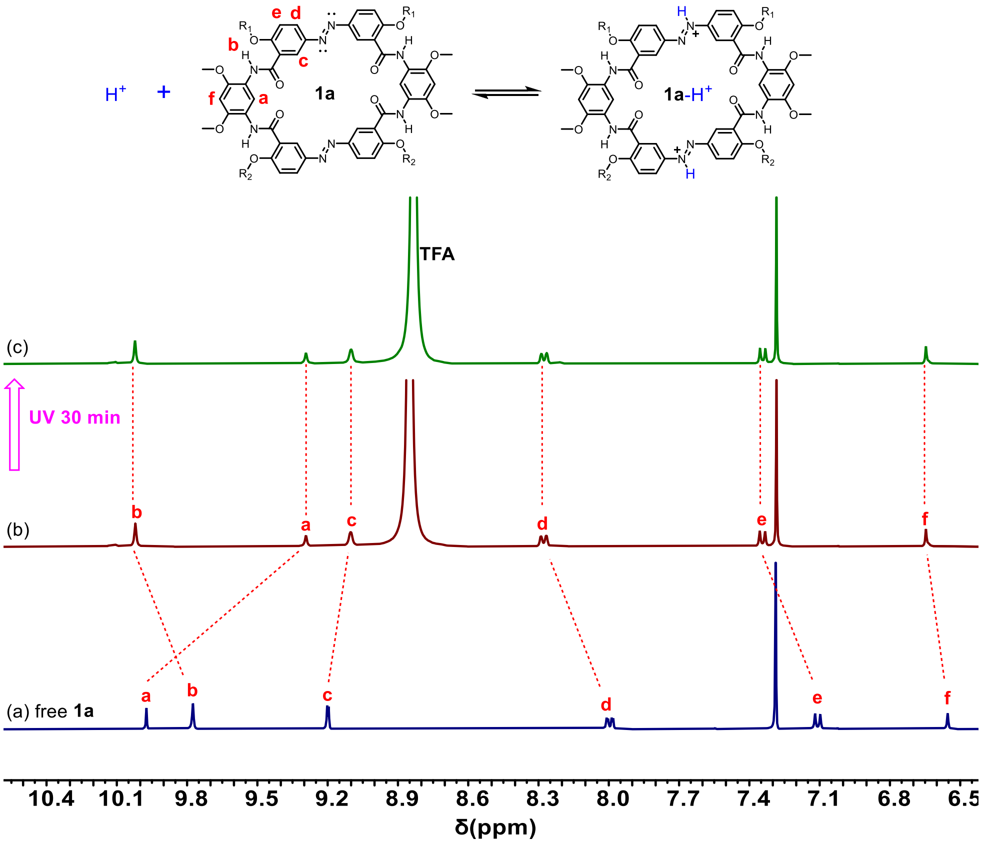

Figure S45 Partial ^{1}H NMR spectra (400 MHz, $CDCl_3$, 298 K) of (a) free **1a** (1.0 mM), (b) **1a** (1.0 mM) + TFA (4.0 mM) before UV irradiation, and (c) **1a** (1.0 mM) + TFA (4.0 mM) after UV irradiation.

Acid/base responsive of host-guest complexes

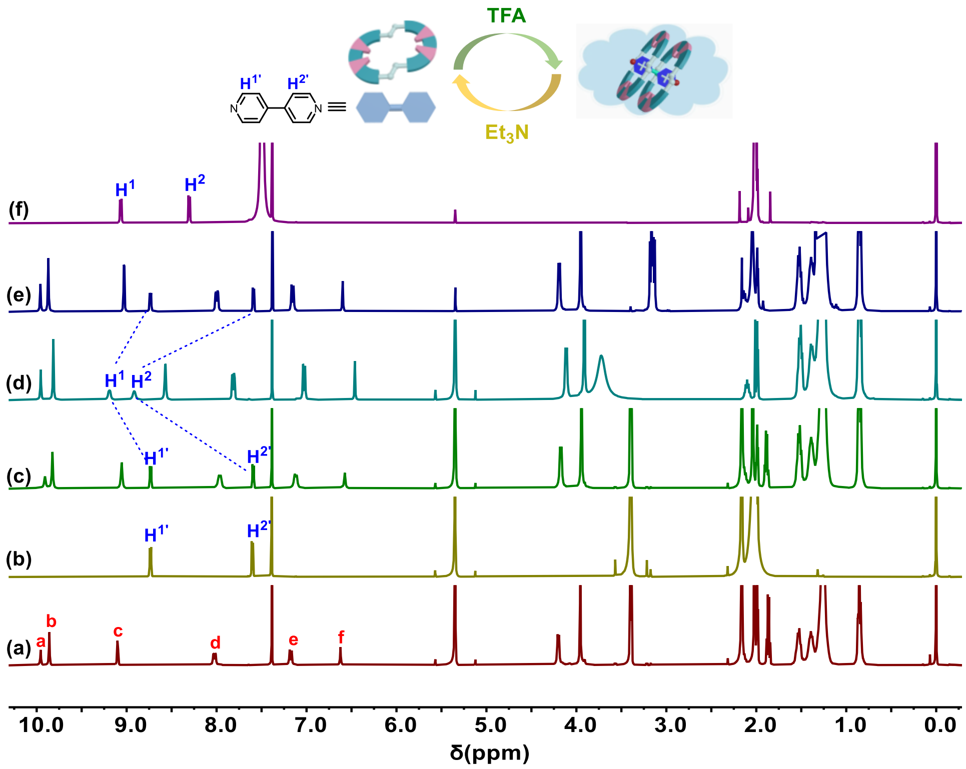

Figure S46 ^{1}H NMR spectra (400 MHz, $CDCl_3$-CD_3CN, 5:1, v/v, 298 K) of (a) free **1a**, (b) free 4,4'-bipyridine, (c) 4,4'-bipyridine and 2.0 equiv. of **1a**, (d) to the solution of c was added 4.0 equiv. of TFA, (e) to the solution of (d) was added 6.0 equiv. of Et_3N and (f) free **G1**. [**1a**]$_0$= 2.0 mM.

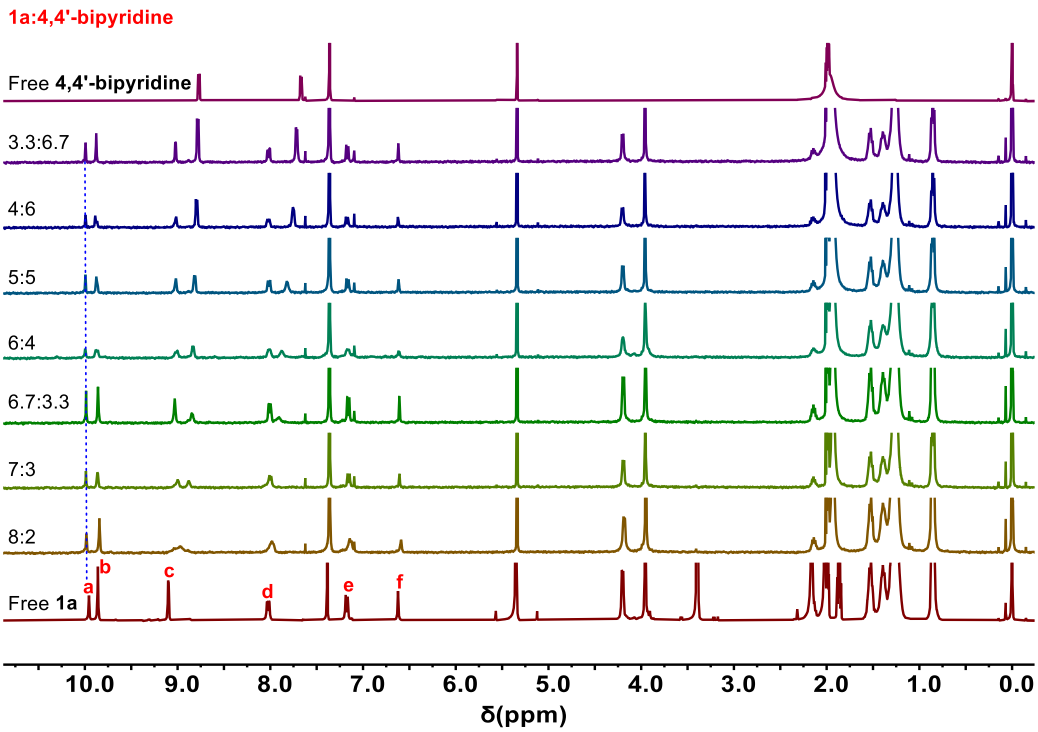

Figure S47 Stack ^{1}H NMR spectra ($CDCl_3$-CD_3CN, 5:1, v/v, 400 MHz, 298 K) utilized in the job plot between **1a** and 4,4'-bipyridine. [**1a**] + [4,4'-bipyridine] = 1.0 mM.

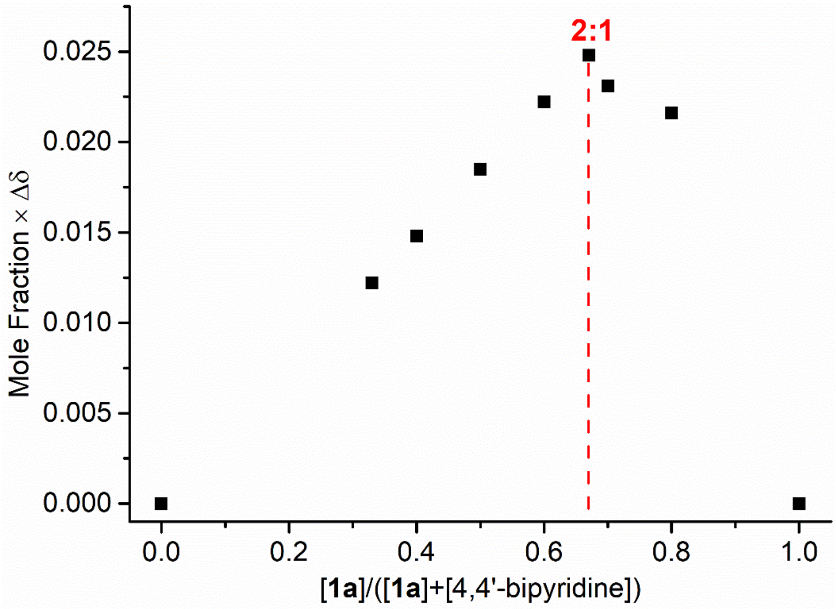

Figure S48 Job plot showing a peak maximum was reached around 0.67 corresponding to the formation of a 2:1 host-guest complex between **1a** and 4,4'-bipyridine.

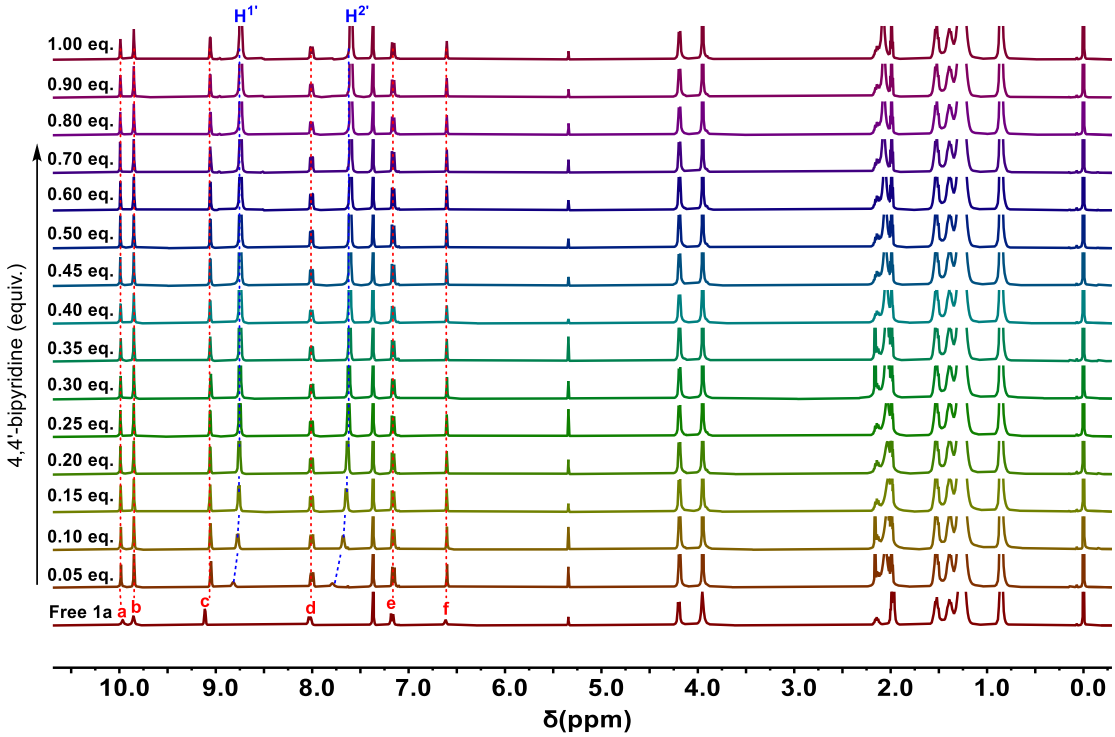

Figure S49 Stack ^{1}H NMR (400 MHz, 298 K, $CDCl_3$-CD_3CN, 5:1, v/v) titration spectra of **1a** at a concentration of 2.0 mM with different equiv of 4,4'-bipyridine. The binding constant fitting based on the data failed, indicating that the binding affinity between **1a** and 4,4'-bipyridine is too low to be determined.

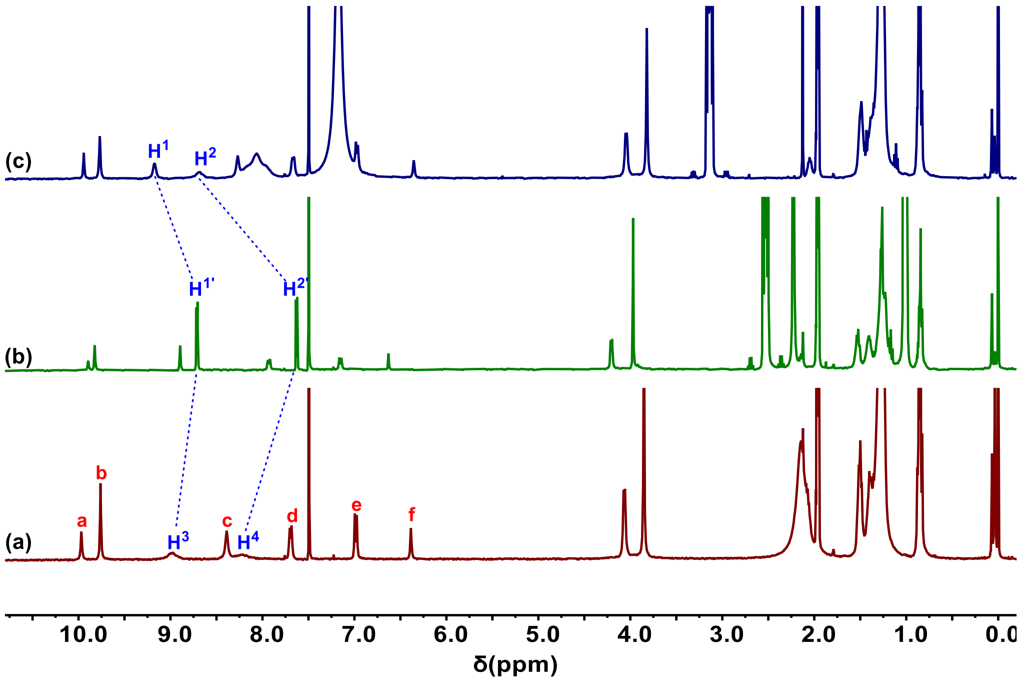

Figure S50 ^{1}H NMR spectra (400 MHz, $CDCl_3$-CD_3CN, 1:1, v/v, 298 K) of (a) 2.0 mM **1a** and 1.0 mM **G2**, (b) to the solution of (a) was added 4.0 equiv. of Et_3N, (c) to the solution of (b) was added 6.0 equiv. of TFA.

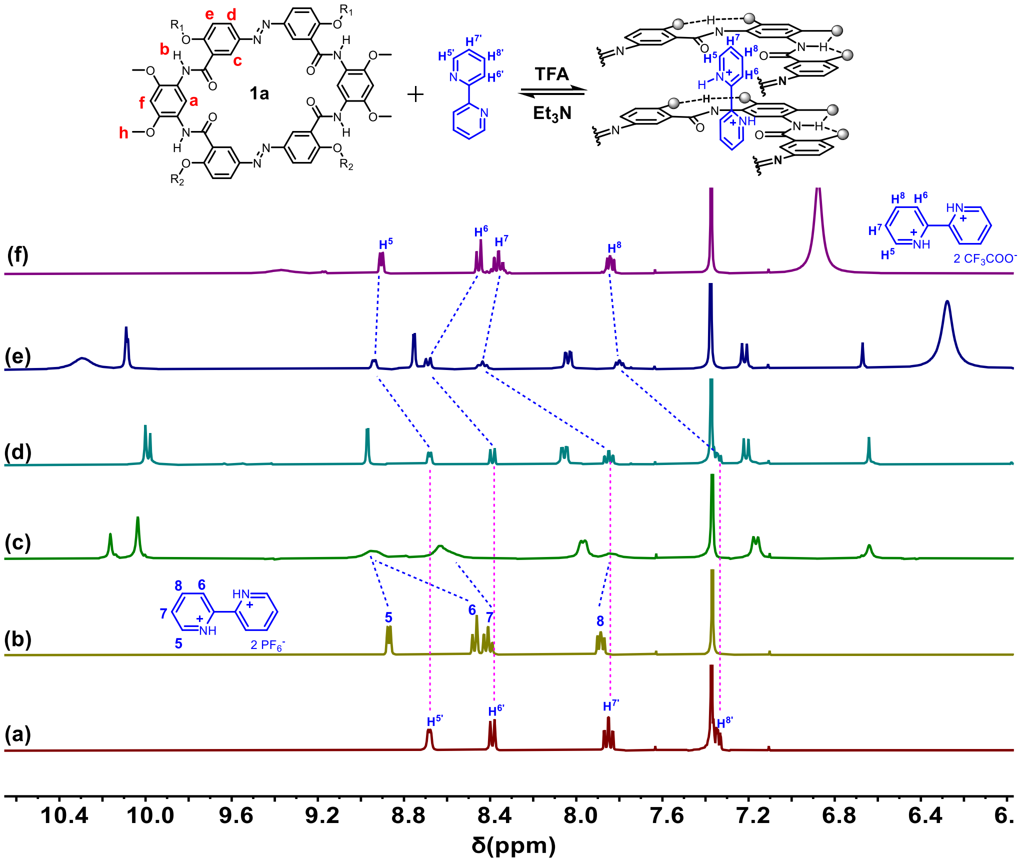

Figure S51 Partial ^{1}H NMR spectra (400 MHz, $CDCl_3$-CD_3CN, 5:1, v/v, 298 K) of (a) free 2,2'-bipyridine, (b) free **G3**, (c) **G3** (1 equiv.) and **1a** (2.0 equiv.), (d) the solution of (c) after adding 4.0 equiv. of Et_3N, (e) the solution of (d) after adding 8.0 equiv. of CF_3COOH, and (f) 2,2'-bipyridine (1 equiv.) and CF_3COOH (4 equiv.) $[\mathbf{G3}]_0$= 1.0 mM.

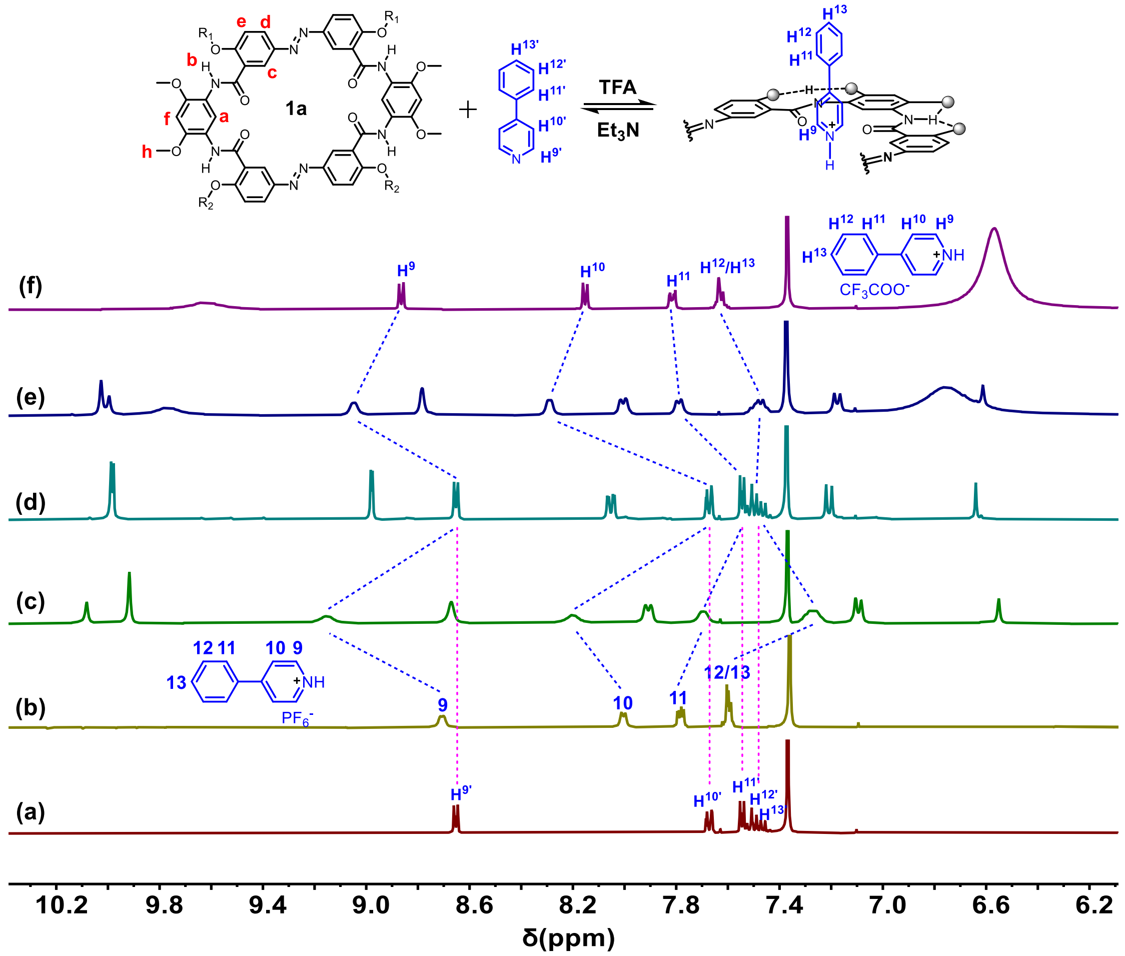

Figure S52 Partial ^{1}H NMR spectra (400 MHz, $CDCl_3$-CD_3CN, 5:1, v/v, 298 K) of (a) free 4-phenylpyridine, (b) free **G4**, (c) **G4** (1 equiv.) and **1a** (1.0 equiv.), (d) the solution of (c) after adding 2.0 equiv. of Et_3N, (e) the solution of (d) after adding 4.0 equiv. of CF_3COOH, and (f) 4-phenylbipyridine (1 equiv.) and CF_3COOH (4 equiv.) [**G4**]$_0$= 1.0 mM.

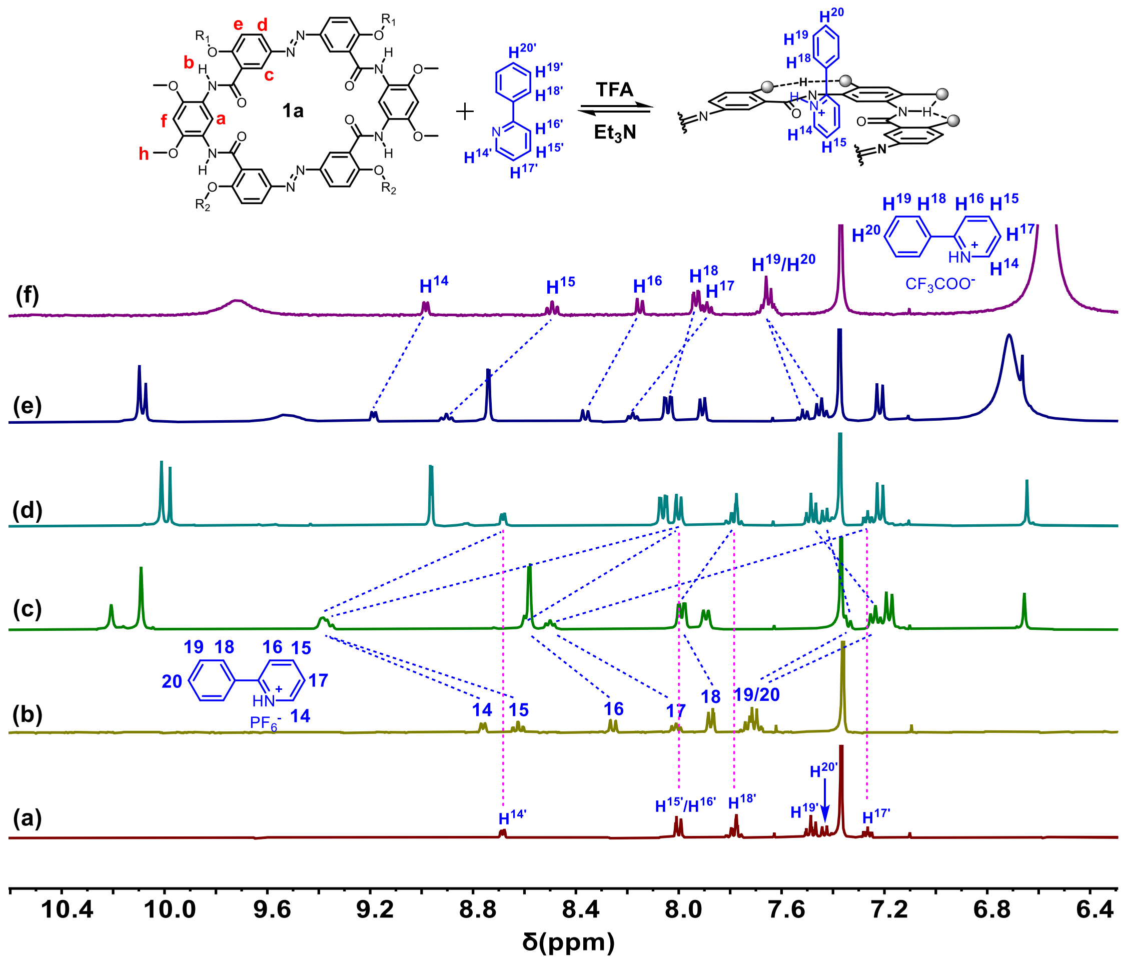

Figure S53 Partial ^{1}H NMR spectra (400 MHz, $CDCl_3$-CD_3CN, 5:1, v/v, 298 K) of (a) free 2-phenylpyridine, (b) free **G5**, (c) **G5** (1 equiv.) and **1a** (1.0 equiv.), (d) the solution of (c) after adding 2.0 equiv. of Et_3N, (e) the solution of (d) after adding 4.0 equiv. of CF_3COOH, and (f) 2-phenylbipyridine (1 equiv.) and CF_3COOH (4 equiv.) $[\textbf{G5}]_0$= 1.0 mM.

Cation-responsive release of the guest in G2⊂1a₂

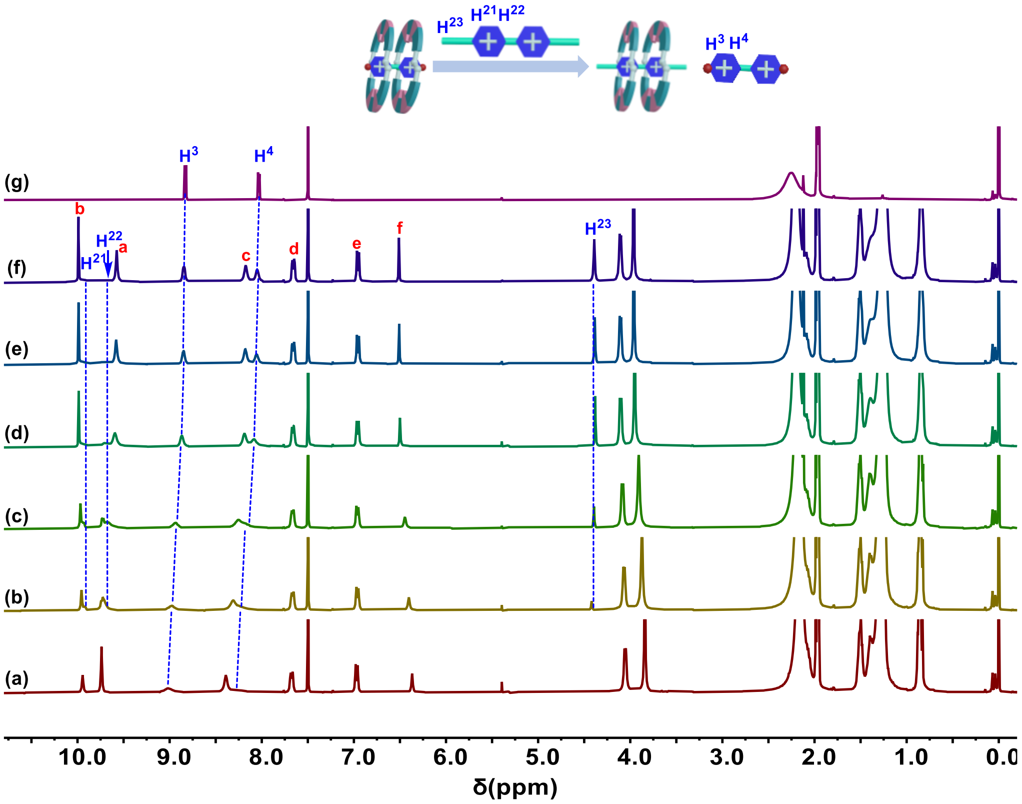

Figure S54 ^{1}H NMR spectra (400 MHz, $CDCl_3$-CD_3CN, 1:1, v/v, 298 K) of (a) 2 mM **1a** and 1 mM **G2**, and containing the following concentrations of **G6** (b) 0.2 mM, (c) 0.4 mM, (d) 0.8 mM, (e) 1.0 mM, (f) 1.2 mM, (g) **G2** alone.

DFT calculation studies

All side chains of **1** are replaced by CH_3 for simplicity. The geometries of **G1**⊂E,E-**1c₂**, **G1**⊂E,Z-**1c₂**, and **G1**⊂Z,Z-**1c₂** were fully optimized at the B3LYP(PCM, chloroform)/ 6-31G (d,p) level of theory with the keyword UAHF method. The contained structures were confirmed without any imaginary frequency. All calculations were performed in Gaussian 09 program package. [5]

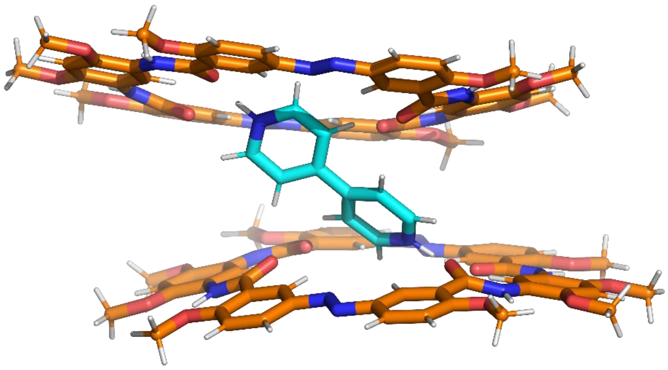

Figure S55 Optimized structure at the DFT/B3LYP(PCM, chloroform)/6-31G (d,p) level of complex **G1**⊂E,E-**1c₂**. All peripheral R^1 and R^2 group are replaced by CH_3 for simplicity.

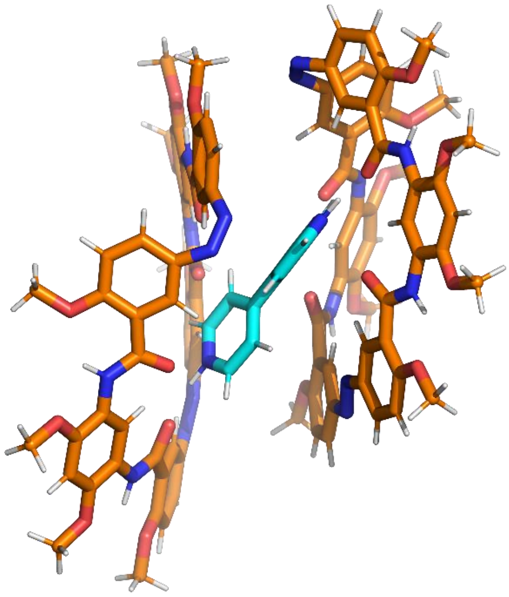

Figure S56 Optimized structure at the DFT/B3LYP(PCM, chloroform)/6-31G (d,p) level of complex **G1**⊂E,Z-**1c$_2$**. All peripheral R^1 and R^2 group are replaced by CH_3 for simplicity.

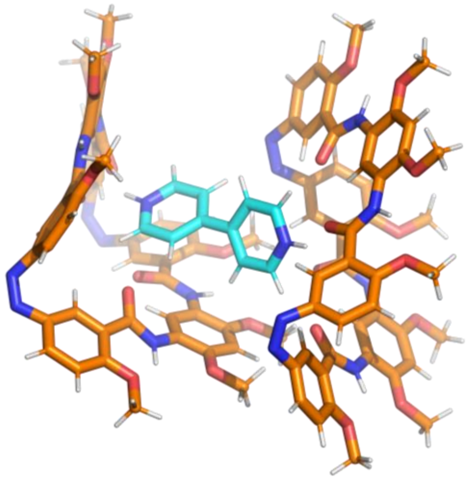

Figure S57 Optimized structure at the DFT/B3LYP(PCM, chloroform)/6-31G (d,p) level of complex **G1**⊂Z,Z-**1c$_2$**. All peripheral R^1 and R^2 group are replaced by CH_3 for simplicity.

Independent gradient model (IGM) analysis is an approach[6] based on promolecular density (an electron density model prior to molecule formation) to identify and isolate intermolecular interactions. DFT optimize structures are used as input files. Strong polar attractions, van der Waals contacts, and repulsive forces are visualized as an isosurface with blue, green, and red color, respectively. The binding surface was calculated by Multiwfn 3.8 program[7] and visualized using PyMOL.[8]

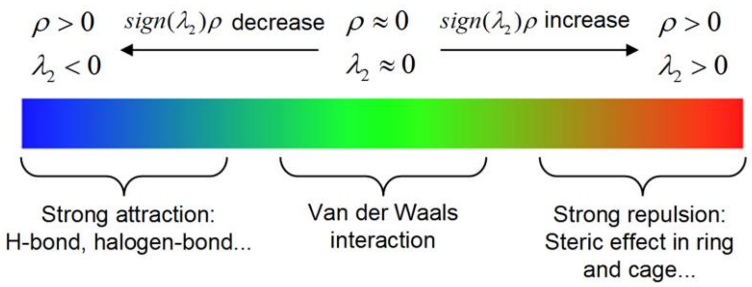

Figure S58 Color-coded sign $(\lambda_2)_\rho$ scale bar.

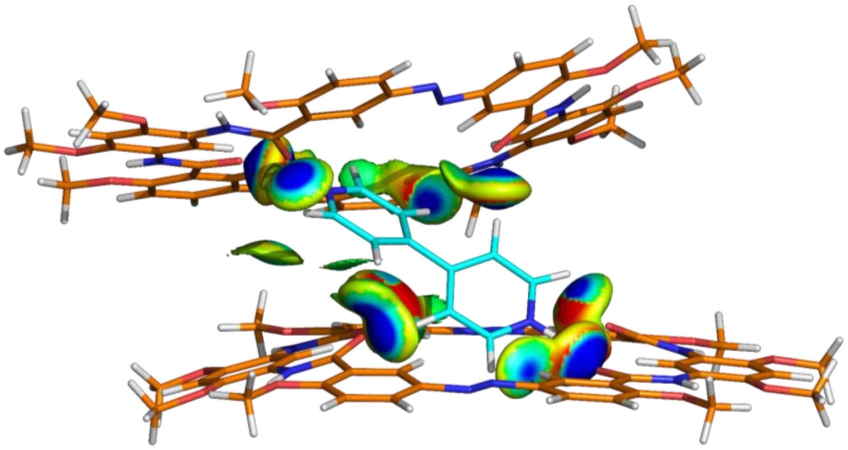

Figure S59 Visualization of noncovalent interactions in complex **G1 ⊂ 1c₂**. Independent gradient model (IGM) analysis revealed that the interfacial interaction in **G1 ⊂ 1c₂** is maintained by C-H···O interaction between macrocycle **1c** and **G1**.

Standard orientation for the optimized structure of E,E-**1₂⊃G**1

Center Number	Atomic Number	Atomic Type	Coordinates (Angstroms) X	Y	Z
1	8	0	-6.070184	2.115816	-2.321806
2	8	0	-3.719345	1.530176	-5.642602
3	8	0	-2.658096	4.440859	-0.688270
4	8	0	0.000225	7.624048	-1.116171
5	8	0	1.539225	5.301556	1.228443
6	8	0	0.083085	1.966253	4.487693
7	8	0	-4.338572	2.956816	2.718782
8	8	0	-2.745065	-0.307726	4.651876
9	8	0	-9.654400	-3.391415	1.082362
10	8	0	-6.955266	-0.218973	0.659458
11	8	0	-7.840656	-4.106183	-1.949612
12	8	0	-5.526495	-1.593837	-5.438671
13	7	0	-5.698969	0.618278	-4.016089
14	7	0	-4.873952	7.641273	-3.858037
15	7	0	-0.951630	5.553997	0.345792
16	7	0	-2.400303	2.193395	3.673842
17	7	0	-8.107122	1.093729	5.444334
18	7	0	-8.000023	-1.924747	-0.451830
19	6	0	-5.637558	1.860800	-3.451668
20	6	0	-5.040384	2.964743	-4.286020
21	6	0	-4.077958	2.807524	-5.312155

22	6	0	-3.530039	3.937354	-5.930352
23	6	0	-3.935446	5.216181	-5.555217
24	6	0	-4.913675	5.381751	-4.569808
25	6	0	-5.464492	4.256515	-3.958229
26	6	0	-3.579765	7.524581	-3.253469
27	6	0	-2.706483	8.607288	-3.412577
28	6	0	-1.493492	8.639582	-2.735000
29	6	0	-1.163557	7.626384	-1.822801
30	6	0	-2.062882	6.552372	-1.604487
31	6	0	-3.246254	6.515253	-2.345957
32	6	0	-1.904022	5.420838	-0.619670
33	6	0	-0.704483	4.634577	1.389816
34	6	0	0.618570	4.506903	1.851055
35	6	0	0.915497	3.615938	2.889656
36	6	0	-0.104449	2.861071	3.474645
37	6	0	-1.440141	3.000067	3.032773
38	6	0	-1.723451	3.896864	1.999749
39	6	0	-3.752471	2.189722	3.489822
40	6	0	-4.549394	1.196846	4.301726
41	6	0	-4.073356	-0.036632	4.810852
42	6	0	-4.960835	-0.919186	5.439493
43	6	0	-6.305803	-0.588660	5.581894
44	6	0	-6.779184	0.643056	5.117325
45	6	0	-5.893893	1.526264	4.499440
46	6	0	-9.148404	-0.546123	4.090074
47	6	0	-10.086051	-1.572324	4.255551
48	6	0	-10.246476	-2.544661	3.275201
49	6	0	-9.520518	-2.473522	2.077474
50	6	0	-8.607971	-1.406851	1.868495
51	6	0	-8.434147	-0.471505	2.892900
52	6	0	-7.786768	-1.150301	0.637272
53	6	0	-7.361265	-1.819472	-1.708893
54	6	0	-7.290545	-2.982575	-2.498169
55	6	0	-6.687986	-2.932549	-3.759392
56	6	0	-6.152975	-1.732652	-4.232556
57	6	0	-6.239816	-0.554709	-3.456705
58	6	0	-6.862972	-0.613353	-2.208029
59	6	0	-2.788367	1.320033	-6.705951
60	6	0	0.920815	8.710929	-1.261114
61	6	0	2.899378	5.221394	1.649033
62	6	0	1.411516	1.717710	4.951034
63	6	0	-2.215273	-1.542856	5.139891
64	6	0	-10.600626	-4.458923	1.217823
65	6	0	-7.917085	-5.287246	-2.745519
66	6	0	-5.494166	-2.707810	-6.328487
67	1	0	-5.262669	0.504762	-4.923916
68	1	0	-0.282242	6.307029	0.231802
69	1	0	-2.021216	1.502611	4.310898
70	1	0	-8.625815	-2.715490	-0.330172
71	1	0	-2.784967	3.831118	-6.708337
72	1	0	-3.510026	6.082754	-6.050739
73	1	0	-6.217875	4.367226	-3.186704
74	1	0	-2.982674	9.419941	-4.076701
75	1	0	-0.820165	9.471168	-2.897199
76	1	0	-3.917604	5.690167	-2.151873
77	1	0	1.932454	3.507803	3.237430
78	1	0	-2.741347	4.009018	1.668483
79	1	0	-4.610958	-1.869409	5.822284
80	1	0	-6.980300	-1.279083	6.077313
81	1	0	-6.246779	2.480292	4.124416
82	1	0	-10.683136	-1.606929	5.161077
83	1	0	-10.957738	-3.344068	3.436657

84	1	0	-7.745306	0.340092	2.705604
85	1	0	-6.629863	-3.825102	-4.365166
86	1	0	-6.964725	0.291656	-1.635240
87	1	0	-3.159857	1.737459	-7.647561
88	1	0	-2.690756	0.238787	-6.799840
89	1	0	1.285662	8.780645	-2.290529
90	1	0	0.460714	9.657967	-0.962636
91	1	0	3.008535	5.505888	2.702019
92	1	0	3.447284	5.927509	1.025011
93	1	0	1.870987	2.632692	5.341411
94	1	0	2.030177	1.287792	4.157554
95	1	0	-1.146011	-1.502092	4.935285
96	1	0	-2.651600	-2.394616	4.608793
97	1	0	-11.617359	-4.068875	1.321321
98	1	0	-10.353121	-5.094099	2.073554
99	1	0	-8.431472	-6.027531	-2.132883
100	1	0	-6.918932	-5.660501	-3.001372
101	1	0	-6.506128	-3.043876	-6.579341
102	1	0	-4.925917	-3.542119	-5.901493
103	1	0	1.750150	8.479595	-0.594017
104	1	0	3.298796	4.213984	1.494686
105	1	0	1.310057	0.996022	5.761952
106	1	0	-2.378161	-1.645815	6.217730
107	1	0	-1.812175	1.756281	-6.469030
108	1	0	-4.995226	-2.355283	-7.230954
109	1	0	-10.520157	-5.035512	0.297433
110	1	0	-8.488591	-5.111354	-3.663535
111	7	0	-9.142968	0.500499	5.070480
112	7	0	-5.504020	6.677193	-4.344377
113	7	0	-4.383501	0.233834	0.060835
114	6	0	-3.761947	-0.959684	0.110419
115	6	0	-2.405981	-1.046482	-0.134571
116	6	0	-1.683024	0.123940	-0.423926
117	6	0	-2.360988	1.349711	-0.437855
118	6	0	-3.728753	1.378885	-0.199084
119	6	0	-0.232147	0.034786	-0.739206
120	6	0	0.713464	0.886281	-0.143323
121	6	0	2.056253	0.748268	-0.461381
122	7	0	2.430707	-0.184219	-1.360440
123	6	0	1.558905	-1.019215	-1.953409
124	6	0	0.213527	-0.935850	-1.648887
125	1	0	-4.380921	-1.816221	0.343500
126	1	0	-1.923178	-2.013763	-0.079995
127	1	0	-1.871554	2.292209	-0.650158
128	1	0	-4.308670	2.290957	-0.231245
129	1	0	0.420966	1.631987	0.586333
130	1	0	2.846642	1.343468	-0.014327
131	1	0	1.979692	-1.745785	-2.632273
132	1	0	-0.477434	-1.612578	-2.135176
133	8	0	4.517281	-1.171819	-2.741725
134	8	0	8.621268	-1.122707	-2.969327
135	8	0	4.640764	1.956701	1.037496
136	8	0	7.480877	4.729718	2.312380
137	8	0	7.683035	2.304728	4.816725
138	8	0	5.383572	-1.707719	6.372039
139	8	0	2.352139	-0.800747	2.739331
140	8	0	3.543159	-4.315286	4.663319
141	8	0	1.091854	-7.865562	-2.170714
142	8	0	2.071698	-3.859205	-1.511469
143	8	0	4.291491	-7.609747	-3.660591
144	8	0	7.680454	-4.257241	-4.708598
145	7	0	6.301273	-2.412529	-3.446016

146	7	0	5.731606	4.630988	-3.003952
147	7	0	6.205956	2.367883	2.654820
148	7	0	3.827315	-1.684058	4.252915
149	7	0	-1.464679	-3.926382	2.195275
150	7	0	2.876350	-5.829485	-2.346227
151	6	0	5.711615	-1.246562	-3.096886
152	6	0	6.542572	0.000706	-3.198683
153	6	0	7.955725	0.057926	-3.097113
154	6	0	8.604772	1.298586	-3.129780
155	6	0	7.875952	2.475164	-3.264725
156	6	0	6.483370	2.430022	-3.397984
157	6	0	5.837752	1.194911	-3.388498
158	6	0	6.251426	4.608504	-1.665920
159	6	0	7.047734	5.670234	-1.227119
160	6	0	7.485523	5.712614	0.093331
161	6	0	7.084079	4.730271	1.011046
162	6	0	6.205801	3.695856	0.598437
163	6	0	5.819618	3.655912	-0.742031
164	6	0	5.612676	2.605752	1.457200
165	6	0	5.926971	1.344663	3.584537
166	6	0	6.748968	1.310132	4.731876
167	6	0	6.587785	0.302147	5.683622
168	6	0	5.604056	-0.673300	5.506367
169	6	0	4.762698	-0.636487	4.374844
170	6	0	4.935315	0.371799	3.421605
171	6	0	2.724420	-1.738687	3.455525
172	6	0	1.927773	-3.024968	3.481682
173	6	0	2.341102	-4.268985	4.025483
174	6	0	1.517373	-5.396150	3.899457
175	6	0	0.298031	-5.313595	3.238411
176	6	0	-0.133723	-4.088580	2.716053
177	6	0	0.671147	-2.959795	2.869906
178	6	0	-1.092113	-5.509456	0.483564
179	6	0	-1.558005	-6.787095	0.156931
180	6	0	-0.825670	-7.597124	-0.706052
181	6	0	0.348118	-7.120468	-1.309796
182	6	0	0.787406	-5.798335	-1.048775
183	6	0	0.059743	-5.027861	-0.140446
184	6	0	1.964439	-5.083560	-1.667046
185	6	0	4.071742	-5.384077	-2.946528
186	6	0	4.828451	-6.354168	-3.639191
187	6	0	6.040856	-6.007176	-4.238738
188	6	0	6.506781	-4.692203	-4.162932
189	6	0	5.751688	-3.713324	-3.484587
190	6	0	4.548038	-4.071637	-2.874259
191	6	0	10.046577	-1.117832	-2.837251
192	6	0	8.381844	5.737831	2.783843
193	6	0	8.559204	2.330617	5.941609
194	6	0	6.229081	-1.838489	7.512868
195	6	0	3.997007	-5.543736	5.240594
196	6	0	0.691550	-9.203457	-2.486448
197	6	0	5.008828	-8.654952	-4.314336
198	6	0	8.520794	-5.197224	-5.376046
199	1	0	7.275193	-2.358453	-3.723957
200	1	0	6.964491	2.989250	2.917612
201	1	0	4.010612	-2.493795	4.834754
202	1	0	2.675727	-6.818167	-2.454773
203	1	0	9.682624	1.354722	-3.050256
204	1	0	8.394974	3.427047	-3.293629
205	1	0	4.761539	1.148229	-3.508027
206	1	0	7.343372	6.446743	-1.925174
207	1	0	8.129995	6.523068	0.408166

208	1	0	5.145471	2.864421	-1.039261
209	1	0	7.224803	0.275108	6.555484
210	1	0	4.302907	0.396535	2.551652
211	1	0	1.825525	-6.346615	4.315193
212	1	0	-0.326716	-6.195667	3.152313
213	1	0	0.352811	-2.001224	2.476526
214	1	0	-2.480197	-7.153207	0.596667
215	1	0	-1.183929	-8.595389	-0.921976
216	1	0	0.409454	-4.019644	0.039360
217	1	0	6.617393	-6.755074	-4.763180
218	1	0	3.982287	-3.334559	-2.335927
219	1	0	10.524126	-0.682974	-3.720948
220	1	0	10.333439	-2.164479	-2.744145
221	1	0	9.327270	5.708825	2.233406
222	1	0	7.933430	6.732648	2.703747
223	1	0	8.000934	2.447926	6.877052
224	1	0	9.206523	3.195391	5.796778
225	1	0	6.144251	-0.968045	8.172960
226	1	0	7.275927	-1.974472	7.219091
227	1	0	4.963455	-5.314321	5.687392
228	1	0	4.120471	-6.314918	4.474255
229	1	0	-0.288972	-9.214410	-2.972204
230	1	0	0.671079	-9.830032	-1.589429
231	1	0	4.402583	-9.552710	-4.194727
232	1	0	5.989218	-8.813222	-3.851600
233	1	0	8.018471	-5.628891	-6.248769
234	1	0	8.835887	-5.998710	-4.698683
235	1	0	8.559195	5.499661	3.831646
236	1	0	9.170337	1.422549	5.990213
237	1	0	5.882134	-2.725955	8.041864
238	1	0	3.307032	-5.893637	6.014896
239	1	0	10.354000	-0.571198	-1.940428
240	1	0	9.394475	-4.634977	-5.704846
241	1	0	1.446511	-9.576035	-3.177428
242	1	0	5.136469	-8.441636	-5.381291
243	7	0	-1.922825	-4.645658	1.275284
244	7	0	5.737221	3.615604	-3.736436
245	1	0	-5.417838	0.218867	0.224743
246	1	0	3.419720	-0.306354	-1.652251

Standard orientation for the optimized structure of E,Z-**1**$_2$⊃**G**1

Center Number	Atomic Number	Atomic Type	Coordinates (Angstroms) X	Y	Z
1	8	0	5.808305	-1.858702	-0.633240
2	8	0	9.845038	-2.699271	0.107395
3	8	0	2.641706	2.903550	-0.015122
4	8	0	5.924159	4.985663	-1.180328
5	8	0	3.076156	7.574491	-1.321870
6	8	0	-1.381533	6.906195	-3.212987
7	8	0	-0.704212	2.322891	-1.666438
8	8	0	-3.823129	4.071313	-3.720079
9	8	0	1.938973	-5.894687	-5.707238
10	8	0	3.031005	-3.021820	-2.848726
11	8	0	5.386212	-6.708566	-4.954401
12	8	0	9.031870	-5.210640	-2.074757
13	7	0	7.548653	-3.242171	-1.150796
14	7	0	6.218063	1.618316	3.259612

15	7	0	3.215698	5.027113	-0.661155
16	7	0	-1.277334	4.327559	-2.614657
17	7	0	-2.230180	-1.132556	-4.990094
18	7	0	3.846928	-4.752242	-4.094617
19	6	0	6.981607	-2.235255	-0.443930
20	6	0	7.811164	-1.580854	0.631905
21	6	0	9.175593	-1.843525	0.925819
22	6	0	9.783027	-1.238946	2.035609
23	6	0	9.057354	-0.393429	2.865362
24	6	0	7.733876	-0.067210	2.551334
25	6	0	7.130239	-0.664149	1.442727
26	6	0	6.219024	2.314513	2.006521
27	6	0	7.389411	2.805491	1.411700
28	6	0	7.310741	3.699122	0.348068
29	6	0	6.065453	4.098941	-0.158553
30	6	0	4.876466	3.572228	0.402237
31	6	0	4.979189	2.714437	1.499350
32	6	0	3.481251	3.805268	-0.122869
33	6	0	2.017583	5.476282	-1.259282
34	6	0	1.967929	6.833273	-1.637851
35	6	0	0.838897	7.338516	-2.286457
36	6	0	-0.242341	6.498506	-2.566631
37	6	0	-0.203122	5.139978	-2.193256
38	6	0	0.924567	4.649415	-1.526264
39	6	0	-1.400864	2.977525	-2.448443
40	6	0	-2.351272	2.254488	-3.373376
41	6	0	-3.508260	2.779789	-4.004718
42	6	0	-4.255694	1.971108	-4.873789
43	6	0	-3.837727	0.672853	-5.165895
44	6	0	-2.681510	0.152848	-4.576614
45	6	0	-1.975439	0.949872	-3.665262
46	6	0	-0.525058	-2.657640	-4.799380
47	6	0	-0.963098	-3.610423	-5.734475
48	6	0	-0.152450	-4.698630	-6.034743
49	6	0	1.110470	-4.855437	-5.428095
50	6	0	1.561680	-3.905640	-4.479667
51	6	0	0.716923	-2.837640	-4.187657
52	6	0	2.873414	-3.870734	-3.736037
53	6	0	5.148979	-4.836223	-3.558125
54	6	0	5.964173	-5.886835	-4.029016
55	6	0	7.269611	-6.031573	-3.553553
56	6	0	7.773168	-5.140286	-2.602751
57	6	0	6.966773	-4.086000	-2.123812
58	6	0	5.667979	-3.941358	-2.616699
59	6	0	11.209459	-3.035993	0.383280
60	6	0	7.092525	5.525651	-1.805219
61	6	0	3.140993	8.928384	-1.762248
62	6	0	-1.353192	8.144974	-3.920818
63	6	0	-4.978108	4.659118	-4.328396
64	6	0	1.554097	-6.875796	-6.677693
65	6	0	6.156054	-7.775203	-5.504552
66	6	0	9.900262	-6.254420	-2.510580
67	1	0	8.516675	-3.456942	-0.928330
68	1	0	3.990951	5.674605	-0.731246
69	1	0	-1.961670	4.784095	-3.207047
70	1	0	3.616716	-5.435703	-4.807883
71	1	0	10.817223	-1.450288	2.273929
72	1	0	9.518502	0.026081	3.753743
73	1	0	6.091481	-0.463604	1.215095
74	1	0	8.361628	2.527440	1.802522
75	1	0	8.226189	4.087979	-0.079282
76	1	0	4.069624	2.350452	1.962493

77	1	0	0.802905	8.378078	-2.579342
78	1	0	0.958851	3.617113	-1.230721
79	1	0	-5.152379	2.355502	-5.342745
80	1	0	-4.396443	0.062454	-5.868320
81	1	0	-1.082657	0.555615	-3.201728
82	1	0	-1.923447	-3.488689	-6.223254
83	1	0	-0.497734	-5.429279	-6.755272
84	1	0	1.056766	-2.099092	-3.473737
85	1	0	7.889602	-6.836702	-3.919873
86	1	0	5.056269	-3.131147	-2.269981
87	1	0	11.846509	-2.147419	0.343114
88	1	0	11.500008	-3.730286	-0.403587
89	1	0	7.696783	6.094325	-1.090889
90	1	0	7.698419	4.733042	-2.255058
91	1	0	3.071185	8.996166	-2.853734
92	1	0	4.111037	9.303969	-1.436446
93	1	0	-0.516675	8.177179	-4.627364
94	1	0	-1.284020	8.997960	-3.236053
95	1	0	-4.996158	5.690857	-3.980689
96	1	0	-5.890416	4.148471	-4.004405
97	1	0	1.411607	-6.418346	-7.661363
98	1	0	0.640587	-7.393133	-6.369772
99	1	0	5.503283	-8.276089	-6.219325
100	1	0	6.460182	-8.487052	-4.729179
101	1	0	10.101978	-6.180252	-3.584972
102	1	0	9.480741	-7.240597	-2.282844
103	1	0	6.725390	6.191574	-2.585187
104	1	0	2.348056	9.533132	-1.307474
105	1	0	-2.295497	8.201571	-4.466089
106	1	0	-4.902714	4.638011	-5.420228
107	1	0	11.304053	-3.521887	1.359099
108	1	0	10.830109	-6.121400	-1.958030
109	1	0	2.380574	-7.584177	-6.717499
110	1	0	7.043272	-7.397024	-6.024486
111	7	0	-1.157743	-1.464757	-4.411553
112	7	0	6.965210	0.652067	3.528305
113	7	0	-3.803715	-0.903518	0.006025
114	6	0	-3.243034	-1.940233	-0.643008
115	6	0	-1.866369	-2.033471	-0.731557
116	6	0	-1.066981	-1.050362	-0.128150
117	6	0	-1.690916	0.016681	0.537360
118	6	0	-3.071413	0.064395	0.592117
119	6	0	0.412526	-1.146334	-0.176662
120	6	0	1.047670	-2.393515	-0.073642
121	6	0	2.427610	-2.463783	-0.125744
122	7	0	3.150120	-1.338016	-0.262016
123	6	0	2.580374	-0.120100	-0.354580
124	6	0	1.203027	0.006289	-0.313990
125	1	0	-3.923443	-2.667906	-1.061977
126	1	0	-1.429534	-2.857137	-1.281899
127	1	0	-1.123300	0.784241	1.045252
128	1	0	-3.595085	0.860519	1.103331
129	1	0	0.484396	-3.306455	0.071705
130	1	0	2.978856	-3.390422	-0.058777
131	1	0	3.228046	0.742786	-0.443538
132	1	0	0.762408	0.990019	-0.420144
133	8	0	0.120516	0.458238	3.084085
134	8	0	3.480273	0.463702	5.584488
135	8	0	-3.921494	-4.047729	1.417938
136	8	0	-4.020499	-4.361577	5.553795
137	8	0	-7.197879	-6.311584	4.006128
138	8	0	-10.314852	-4.224098	0.876865

139	8	0	-6.466272	-1.438287	-0.136003
140	8	0	-10.337869	-1.555013	-1.656743
141	8	0	-2.683109	6.990825	0.520093
142	8	0	-2.477816	2.981935	1.805734
143	8	0	0.360890	6.906313	2.276364
144	8	0	3.371100	3.770280	4.498283
145	7	0	1.706656	1.793537	4.060137
146	7	0	0.781843	-4.692589	2.729484
147	7	0	-5.247907	-4.699799	3.162375
148	7	0	-8.446304	-2.540411	0.084306
149	7	0	-7.478732	3.255053	-1.450467
150	7	0	-1.377350	4.987035	1.835997
151	6	0	1.161107	0.586169	3.740904
152	6	0	1.929056	-0.647918	4.167407
153	6	0	3.078549	-0.695056	4.998221
154	6	0	3.760808	-1.907921	5.179108
155	6	0	3.337926	-3.060192	4.527816
156	6	0	2.170102	-3.047028	3.755239
157	6	0	1.477315	-1.842826	3.597427
158	6	0	-0.383942	-4.418935	3.512034
159	6	0	-0.390113	-4.339043	4.912750
160	6	0	-1.596057	-4.323804	5.603173
161	6	0	-2.819669	-4.362606	4.915997
162	6	0	-2.831595	-4.412362	3.498776
163	6	0	-1.605519	-4.472944	2.834593
164	6	0	-4.048416	-4.367197	2.605542
165	6	0	-6.514312	-4.610629	2.541447
166	6	0	-7.545160	-5.446986	3.009012
167	6	0	-8.828483	-5.347026	2.458579
168	6	0	-9.093596	-4.404529	1.462271
169	6	0	-8.072013	-3.541322	1.010537
170	6	0	-6.791222	-3.668961	1.550741
171	6	0	-7.690858	-1.506557	-0.357336
172	6	0	-8.383229	-0.365561	-1.048913
173	6	0	-9.666719	-0.372822	-1.657210
174	6	0	-10.170460	0.804795	-2.228846
175	6	0	-9.439357	1.992778	-2.175353
176	6	0	-8.191468	2.021707	-1.547834
177	6	0	-7.679193	0.830597	-1.023577
178	6	0	-5.653795	4.236106	-0.434762
179	6	0	-5.772803	5.555559	-0.899581
180	6	0	-4.785986	6.479373	-0.578192
181	6	0	-3.664613	6.112578	0.194690
182	6	0	-3.533877	4.785440	0.675173
183	6	0	-4.552184	3.896635	0.353176
184	6	0	-2.412773	4.178028	1.489342
185	6	0	-0.202112	4.636929	2.531475
186	6	0	0.727450	5.674836	2.749672
187	6	0	1.927315	5.418285	3.416286
188	6	0	2.218637	4.124445	3.852020
189	6	0	1.304643	3.073950	3.624880
190	6	0	0.092572	3.346489	2.985451
191	6	0	4.711467	0.505003	6.319363
192	6	0	-4.063714	-4.275934	6.982541
193	6	0	-8.202478	-7.160036	4.557472
194	6	0	-11.406545	-5.036819	1.302864
195	6	0	-11.627595	-1.634106	-2.274781
196	6	0	-2.733693	8.335997	0.034951
197	6	0	1.327523	7.952212	2.273656
198	6	0	4.361572	4.765496	4.734843
199	1	0	2.564571	1.773681	4.602609
200	1	0	-5.243265	-5.007491	4.126718

201	1	0	-9.424185	-2.542144	-0.188377
202	1	0	-1.419874	5.949287	1.517897
203	1	0	4.646926	-1.946685	5.799169
204	1	0	3.908920	-3.978691	4.619343
205	1	0	0.595805	-1.786574	2.971631
206	1	0	0.540436	-4.327045	5.468112
207	1	0	-1.579826	-4.288912	6.684783
208	1	0	-1.617815	-4.553531	1.753719
209	1	0	-9.618786	-5.994100	2.810465
210	1	0	-6.004500	-3.025790	1.205619
211	1	0	-11.140832	0.804069	-2.708148
212	1	0	-9.846030	2.904487	-2.601564
213	1	0	-6.706422	0.848774	-0.557454
214	1	0	-6.629884	5.849212	-1.495637
215	1	0	-4.884777	7.498344	-0.930397
216	1	0	-4.481298	2.883589	0.721170
217	1	0	2.630971	6.219227	3.590802
218	1	0	-0.616533	2.554328	2.830315
219	1	0	4.661390	-0.148503	7.195906
220	1	0	4.815450	1.539922	6.642278
221	1	0	-3.578673	-5.141379	7.444926
222	1	0	-3.593044	-3.352394	7.333186
223	1	0	-9.018615	-6.577193	4.999188
224	1	0	-7.708362	-7.740673	5.336371
225	1	0	-11.620233	-4.888010	2.367144
226	1	0	-11.208069	-6.097536	1.113067
227	1	0	-11.946769	-2.668249	-2.153088
228	1	0	-11.563781	-1.391728	-3.339902
229	1	0	-3.624639	8.853388	0.403344
230	1	0	-2.712278	8.351598	-1.058408
231	1	0	0.840061	8.809120	1.807850
232	1	0	2.210846	7.672755	1.687954
233	1	0	3.980121	5.559292	5.387414
234	1	0	4.717024	5.202479	3.794849
235	1	0	-5.121641	-4.268215	7.241867
236	1	0	-8.607155	-7.840803	3.800176
237	1	0	-12.265434	-4.717828	0.712760
238	1	0	-12.341258	-0.966502	-1.782152
239	1	0	5.556535	0.232746	5.680647
240	1	0	5.186864	4.251975	5.227141
241	1	0	-1.841455	8.819541	0.429942
242	1	0	1.629046	8.223286	3.291867
243	7	0	-6.509570	3.148592	-0.648167
244	7	0	1.911177	-4.184489	2.920649
245	1	0	-4.839816	-0.885600	0.037671
246	1	0	4.181023	-1.435604	-0.337133

Standard orientation for the optimized structure of Z,Z-**1**$_2$**⊃G**1

Center Number	Atomic Number	Atomic type	Coordinates (Angstroms) X	Y	Z
1	8	0	-6.070184	2.115816	-2.321806
2	8	0	-3.719345	1.530176	-5.642602
3	8	0	-2.658096	4.440859	-0.688270
4	8	0	0.000225	7.624048	-1.116171
5	8	0	1.539225	5.301556	1.228443
6	8	0	0.083085	1.966253	4.487693
7	8	0	-4.338572	2.956816	2.718782

8	8	0	-2.745065	-0.307726	4.651876
9	8	0	-9.654400	-3.391415	1.082362
10	8	0	-6.955266	-0.218973	0.659458
11	8	0	-7.840656	-4.106183	-1.949612
12	8	0	-5.526495	-1.593837	-5.438671
13	7	0	-5.698969	0.618278	-4.016089
14	7	0	-4.873952	7.641273	-3.858037
15	7	0	-0.951630	5.553997	0.345792
16	7	0	-2.400303	2.193395	3.673842
17	7	0	-8.107122	1.093729	5.444334
18	7	0	-8.000023	-1.924747	-0.451830
19	6	0	-5.637558	1.860800	-3.451668
20	6	0	-5.040384	2.964743	-4.286020
21	6	0	-4.077958	2.807524	-5.312155
22	6	0	-3.530039	3.937354	-5.930352
23	6	0	-3.935446	5.216181	-5.555217
24	6	0	-4.913675	5.381751	-4.569808
25	6	0	-5.464492	4.256515	-3.958229
26	6	0	-3.579765	7.524581	-3.253469
27	6	0	-2.706483	8.607288	-3.412577
28	6	0	-1.493492	8.639582	-2.735000
29	6	0	-1.163557	7.626384	-1.822801
30	6	0	-2.062882	6.552372	-1.604487
31	6	0	-3.246254	6.515253	-2.345957
32	6	0	-1.904022	5.420838	-0.619670
33	6	0	-0.704483	4.634577	1.389816
34	6	0	0.618570	4.506903	1.851055
35	6	0	0.915497	3.615938	2.889656
36	6	0	-0.104449	2.861071	3.474645
37	6	0	-1.440141	3.000067	3.032773
38	6	0	-1.723451	3.896864	1.999749
39	6	0	-3.752471	2.189722	3.489822
40	6	0	-4.549394	1.196846	4.301726
41	6	0	-4.073356	-0.036632	4.810852
42	6	0	-4.960835	-0.919186	5.439493
43	6	0	-6.305803	-0.588660	5.581894
44	6	0	-6.779184	0.643056	5.117325
45	6	0	-5.893893	1.526264	4.499440
46	6	0	-9.148404	-0.546123	4.090074
47	6	0	-10.086051	-1.572324	4.255551
48	6	0	-10.246476	-2.544661	3.275201
49	6	0	-9.520518	-2.473522	2.077474
50	6	0	-8.607971	-1.406851	1.868495
51	6	0	-8.434147	-0.471505	2.892900
52	6	0	-7.786768	-1.150301	0.637272
53	6	0	-7.361265	-1.819472	-1.708893
54	6	0	-7.290545	-2.982575	-2.498169
55	6	0	-6.687986	-2.932549	-3.759392
56	6	0	-6.152975	-1.732652	-4.232556
57	6	0	-6.239816	-0.554709	-3.456705
58	6	0	-6.862972	-0.613353	-2.208029
59	6	0	-2.788367	1.320033	-6.705951
60	6	0	0.920815	8.710929	-1.261114
61	6	0	2.899378	5.221394	1.649033
62	6	0	1.411516	1.717710	4.951034
63	6	0	-2.215273	-1.542856	5.139891
64	6	0	-10.600626	-4.458923	1.217823
65	6	0	-7.917085	-5.287246	-2.745519
66	6	0	-5.494166	-2.707810	-6.328487
67	1	0	-5.262669	0.504762	-4.923916
68	1	0	-0.282242	6.307029	0.231802
69	1	0	-2.021216	1.502611	4.310898

70	1	0	-8.625815	-2.715490	-0.330172
71	1	0	-2.784967	3.831118	-6.708337
72	1	0	-3.510026	6.082754	-6.050739
73	1	0	-6.217875	4.367226	-3.186704
74	1	0	-2.982674	9.419941	-4.076701
75	1	0	-0.820165	9.471168	-2.897199
76	1	0	-3.917604	5.690167	-2.151873
77	1	0	1.932454	3.507803	3.237430
78	1	0	-2.741347	4.009018	1.668483
79	1	0	-4.610958	-1.869409	5.822284
80	1	0	-6.980300	-1.279083	6.077313
81	1	0	-6.246779	2.480292	4.124416
82	1	0	-10.683136	-1.606929	5.161077
83	1	0	-10.957738	-3.344068	3.436657
84	1	0	-7.745306	0.340092	2.705604
85	1	0	-6.629863	-3.825102	-4.365166
86	1	0	-6.964725	0.291656	-1.635240
87	1	0	-3.159857	1.737459	-7.647561
88	1	0	-2.690756	0.238787	-6.799840
89	1	0	1.285662	8.780645	-2.290529
90	1	0	0.460714	9.657967	-0.962636
91	1	0	3.008535	5.505888	2.702019
92	1	0	3.447284	5.927509	1.025011
93	1	0	1.870987	2.632692	5.341411
94	1	0	2.030177	1.287792	4.157554
95	1	0	-1.146011	-1.502092	4.935285
96	1	0	-2.651600	-2.394616	4.608793
97	1	0	-11.617359	-4.068875	1.321321
98	1	0	-10.353121	-5.094099	2.073554
99	1	0	-8.431472	-6.027531	-2.132883
100	1	0	-6.918932	-5.660501	-3.001372
101	1	0	-6.506128	-3.043876	-6.579341
102	1	0	-4.925917	-3.542119	-5.901493
103	1	0	1.750150	8.479595	-0.594017
104	1	0	3.298796	4.213984	1.494686
105	1	0	1.310057	0.996022	5.761952
106	1	0	-2.378161	-1.645815	6.217730
107	1	0	-1.812175	1.756281	-6.469030
108	1	0	-4.995226	-2.355283	-7.230954
109	1	0	-10.520157	-5.035512	0.297433
110	1	0	-8.488591	-5.111354	-3.663535
111	7	0	-9.142968	0.500499	5.070480
112	7	0	-5.504020	6.677193	-4.344377
113	7	0	-4.383501	0.233834	0.060835
114	6	0	-3.761947	-0.959684	0.110419
115	6	0	-2.405981	-1.046482	-0.134571
116	6	0	-1.683024	0.123940	-0.423926
117	6	0	-2.360988	1.349711	-0.437855
118	6	0	-3.728753	1.378885	-0.199084
119	6	0	-0.232147	0.034786	-0.739206
120	6	0	0.713464	0.886281	-0.143323
121	6	0	2.056253	0.748268	-0.461381
122	7	0	2.430707	-0.184219	-1.360440
123	6	0	1.558905	-1.019215	-1.953409
124	6	0	0.213527	-0.935850	-1.648887
125	1	0	-4.380921	-1.816221	0.343500
126	1	0	-1.923178	-2.013763	-0.079995
127	1	0	-1.871554	2.292209	-0.650158
128	1	0	-4.308670	2.290957	-0.231245
129	1	0	0.420966	1.631987	0.586333
130	1	0	2.846642	1.343468	-0.014327
131	1	0	1.979692	-1.745785	-2.632273

132	1	0	-0.477434	-1.612578	-2.135176
133	8	0	4.517281	-1.171819	-2.741725
134	8	0	8.621268	-1.122707	-2.969327
135	8	0	4.640764	1.956701	1.037496
136	8	0	7.480877	4.729718	2.312380
137	8	0	7.683035	2.304728	4.816725
138	8	0	5.383572	-1.707719	6.372039
139	8	0	2.352139	-0.800747	2.739331
140	8	0	3.543159	-4.315286	4.663319
141	8	0	1.091854	-7.865562	-2.170714
142	8	0	2.071698	-3.859205	-1.511469
143	8	0	4.291491	-7.609747	-3.660591
144	8	0	7.680454	-4.257241	-4.708598
145	7	0	6.301273	-2.412529	-3.446016
146	7	0	5.731606	4.630988	-3.003952
147	7	0	6.205956	2.367883	2.654820
148	7	0	3.827315	-1.684058	4.252915
149	7	0	-1.464679	-3.926382	2.195275
150	7	0	2.876350	-5.829485	-2.346227
151	6	0	5.711615	-1.246562	-3.096886
152	6	0	6.542572	0.000706	-3.198683
153	6	0	7.955725	0.057926	-3.097113
154	6	0	8.604772	1.298586	-3.129780
155	6	0	7.875952	2.475164	-3.264725
156	6	0	6.483370	2.430022	-3.397984
157	6	0	5.837752	1.194911	-3.388498
158	6	0	6.251426	4.608504	-1.665920
159	6	0	7.047734	5.670234	-1.227119
160	6	0	7.485523	5.712614	0.093331
161	6	0	7.084079	4.730271	1.011046
162	6	0	6.205801	3.695856	0.598437
163	6	0	5.819618	3.655912	-0.742031
164	6	0	5.612676	2.605752	1.457200
165	6	0	5.926971	1.344663	3.584537
166	6	0	6.748968	1.310132	4.731876
167	6	0	6.587785	0.302147	5.683622
168	6	0	5.604056	-0.673300	5.506367
169	6	0	4.762698	-0.636487	4.374844
170	6	0	4.935315	0.371799	3.421605
171	6	0	2.724420	-1.738687	3.455525
172	6	0	1.927773	-3.024968	3.481682
173	6	0	2.341102	-4.268985	4.025483
174	6	0	1.517373	-5.396150	3.899457
175	6	0	0.298031	-5.313595	3.238411
176	6	0	-0.133723	-4.088580	2.716053
177	6	0	0.671147	-2.959795	2.869906
178	6	0	-1.092113	-5.509456	0.483564
179	6	0	-1.558005	-6.787095	0.156931
180	6	0	-0.825670	-7.597124	-0.706052
181	6	0	0.348118	-7.120468	-1.309796
182	6	0	0.787406	-5.798335	-1.048775
183	6	0	0.059743	-5.027861	-0.140446
184	6	0	1.964439	-5.083560	-1.667046
185	6	0	4.071742	-5.384077	-2.946528
186	6	0	4.828451	-6.354168	-3.639191
187	6	0	6.040856	-6.007176	-4.238738
188	6	0	6.506781	-4.692203	-4.162932
189	6	0	5.751688	-3.713324	-3.484587
190	6	0	4.548038	-4.071637	-2.874259
191	6	0	10.046577	-1.117832	-2.837251
192	6	0	8.381844	5.737831	2.783843
193	6	0	8.559204	2.330617	5.941609

194	6	0	6.229081	-1.838489	7.512868
195	6	0	3.997007	-5.543736	5.240594
196	6	0	0.691550	-9.203457	-2.486448
197	6	0	5.008828	-8.654952	-4.314336
198	6	0	8.520794	-5.197224	-5.376046
199	1	0	7.275193	-2.358453	-3.723957
200	1	0	6.964491	2.989250	2.917612
201	1	0	4.010612	-2.493795	4.834754
202	1	0	2.675727	-6.818167	-2.454773
203	1	0	9.682624	1.354722	-3.050256
204	1	0	8.394974	3.427047	-3.293629
205	1	0	4.761539	1.148229	-3.508027
206	1	0	7.343372	6.446743	-1.925174
207	1	0	8.129995	6.523068	0.408166
208	1	0	5.145471	2.864421	-1.039261
209	1	0	7.224803	0.275108	6.555484
210	1	0	4.302907	0.396535	2.551652
211	1	0	1.825525	-6.346615	4.315193
212	1	0	-0.326716	-6.195667	3.152313
213	1	0	0.352811	-2.001224	2.476526
214	1	0	-2.480197	-7.153207	0.596667
215	1	0	-1.183929	-8.595389	-0.921976
216	1	0	0.409454	-4.019644	0.039360
217	1	0	6.617393	-6.755074	-4.763180
218	1	0	3.982287	-3.334559	-2.335927
219	1	0	10.524126	-0.682974	-3.720948
220	1	0	10.333439	-2.164479	-2.744145
221	1	0	9.327270	5.708825	2.233406
222	1	0	7.933430	6.732648	2.703747
223	1	0	8.000934	2.447926	6.877052
224	1	0	9.206523	3.195391	5.796778
225	1	0	6.144251	-0.968045	8.172960
226	1	0	7.275927	-1.974472	7.219091
227	1	0	4.963455	-5.314321	5.687392
228	1	0	4.120471	-6.314918	4.474255
229	1	0	-0.288972	-9.214410	-2.972204
230	1	0	0.671079	-9.830032	-1.589429
231	1	0	4.402583	-9.552710	-4.194727
232	1	0	5.989218	-8.813222	-3.851600
233	1	0	8.018471	-5.628891	-6.248769
234	1	0	8.835887	-5.998710	-4.698683
235	1	0	8.559195	5.499661	3.831646
236	1	0	9.170337	1.422549	5.990213
237	1	0	5.882134	-2.725955	8.041864
238	1	0	3.307032	-5.893637	6.014896
239	1	0	10.354000	-0.571198	-1.940428
240	1	0	9.394475	-4.634977	-5.704846
241	1	0	1.446511	-9.576035	-3.177428
242	1	0	5.136469	-8.441636	-5.381291
243	7	0	-1.922825	-4.645658	1.275284
244	7	0	5.737221	3.615604	-3.736436
245	1	0	-5.417838	0.218867	0.224743
246	1	0	3.419720	-0.306354	-1.652251

References

[1] Ye, Z.; Yang, Z.; Wang, L.; Chen, L.; Cai, Y.; Deng, P.; Feng, W.; Li, X.; Yuan, L., A Dynamic Hydrogen-Bonded Azo-Macrocycle for Precisely Photo-Controlled Molecular Encapsulation and Release. *Angew. Chem. Int. Ed.* **2019**, *131*, 12649-12653.

[2] Cheng, M.; Zhang, J.; Ren, X.; Guo, S.; Xiao, T.; Hu, X.-Y.; Jiang, J.; Wang, L., Acid/base-Controllable Fluorescent Molecular Switches Based on Cryptands and Basic N-heteroaromatics. *Chem. Commun.* **2017**, *53*, 11838-11841.

[3] Han, Y.; Meng, Z.; Chen, C.-F., Acid/Base Controllable Complexation of a Triptycene-Derived Macrotricyclic Host and Protonated 4,4′-Bipyridinium/Pyridinium Salts. *Chem. Commun.* **2016**, *52*, 590-593.

[4] Xiao, Y.; Chu L.; Sanakis, Y.; Liu, P., Revisiting the IspH Catalytic System in the Deoxyxylulose Phosphate Pathway: Achieving High Activity. *J. Am. Chem. Soc.* **2009**, *131*, 9931.

[5] Gaussian 09, Revision B.01, M. J. Frisch, G. W. Trucks, H. B. Schlegel, G. E. Scuseria, M. A. Robb, J. R. Cheeseman, G. Scalmani, V. Barone, B. Mennucci, G. A. Petersson, H. Nakatsuji, M. Caricato, X. Li, H. P. Hratchian, A. F. Izmaylov, J. Bloino, G. Zheng, J. L. Sonnenberg, M. Hada, M. Ehara, K. Toyota, R. Fukuda, J. Hasegawa, M. Ishida, T. Nakajima, Y. Honda, O. Kitao, H. Nakai, T. Vreven, J. A. Montgomery, Jr., J. E. Peralta, F. Ogliaro, M. Bearpark, J. J. Heyd, E. Brothers, K. N. Kudin, V. N. Staroverov, T. Keith, R. Kobayashi, J. Normand, K. Raghavachari, A. Rendell, J. C. Burant, S. S. Iyengar, J. Tomasi, M. Cossi, N. Rega, J. M. Millam, M. Klene, J. E. Knox, J. B. Cross, V. Bakken, C. Adamo, J. Jaramillo, R. Gomperts, R. E. Stratmann,O. Yazyev, A. J. Austin, R. Cammi, C. Pomelli, J. W. Ochterski, R. L. Martin, K. Morokuma, V. G. Zakrzewski, G. A. Voth, P. Salvador, J. J. annenberg, S. Dapprich, A. D. Daniels, O. Farkas, J. B. Foresman, J. V. Ortiz, J. Cioslowski, and D. J. Fox, Gaussian, Inc., Wallingford CT, USA, **2013**.

[6] Lefebvre, C.; Rubez, G.; Khartabil, H.; Boisson, J.-C.; Contreras-García, J.; Hénon, E. Accurately Extracting the Signature of Intermolecular Interactions Present in the NCI Plot of the Reduced Density Gradient Versus Electron Density. *Phys. Chem. Chem. Phys.* **2017**, *19*, 17928-17936.

[7] Lu, T.; Chen, F. Multiwfn: A Multifunctional Wavefunction Analyzer. *J. Comput. Chem.* **2012**, *33*, 580-592.

[8] The PyMOL Molecular Graphics System, Version 2.3.0 Schrödinger, LLC: New York, NY, USA, **2019**.
